# The oncogenome of the domestic cat

**DOI:** 10.1126/science.ady6651

**Published:** 2026-02-19

**Authors:** Bailey A. Francis, Latasha Ludwig, Chang He, Melanie Dobromylskyj, Christof A. Bertram, Heike Aupperle-Lellbach, Hannah Wong, Aiden P. Foster, Mark J. Arends, Alejandro Suárez-Bonnet, Simon L. Priestnall, Laetitia Tatiersky, Fernanda Castillo-Alcala, Angie Rupp, Arlene Khachadoorian, Eda Parlak, Marine Inglebert, Shevaniee Umamaheswaran, Saamin Cheema, Martin Del Castillo Velasco-Herrera, Kim Wong, Ian C. Vermes, Jamie Billington, Sven Rottenberg, Geoffrey A. Wood, David J. Adams, Louise van der Weyden

**Affiliations:** 1 Wellcome Sanger Institute, Wellcome Genome Campus, Hinxton, Cambridge, Cambridgeshire, CB10 1SA, UK; 2 Department of Pathobiology, Ontario Veterinary College, University of Guelph, Guelph, Ontario, NIG 2W1, Canada; 3 Department of Population Medicine and Diagnostic Sciences, Cornell University, College of Veterinary Medicine, Ithaca, NY, 14853, USA; 4 Institute of Animal Pathology, Vetsuisse Faculty, University of Bern, 3012 Bern, Switzerland; 5 Graduate School for Cellular and Biomedical Sciences, University of Bern, 3012 Bern, Switzerland; 6 Finn Pathologists, One-Eyed Lane, Weybread, Diss, Norfolk, IP21 5TT, UK; 7 Institute of Pathology, University of Veterinary Medicine Vienna, Wien, 1210, Austria; 8 Laboklin GmbH & Co. KG, Laboratory for Clinical Diagnostics, 97688 Bad Kissingen, Germany; 9 Institute of Pathology, School of Medicine, Technical University of Munich, Trogerstrasse 18, 81675 Munich, Germany; 10 Department of Veterinary Medicine, University of Cambridge, Cambridge, Cambridgeshire, CB3 0ES, UK; 11 Bristol Veterinary School, University of Bristol, Langford House, Langford, North Somerset, BS40 5DU, UK; 12 University of Edinburgh, Edinburgh Pathology, Centre for Comparative Pathology, CRUK Scotland Centre, Institute of Genetics and Cancer, Crewe Road South, Edinburgh, EH4 2XR, Scotland; 13 Department of Pathobiology and Population Sciences, Royal Veterinary College, Hawkshead Lane, North Mymms, Hatfield AL9 7TA, UK; 14 VETPATH Canada, Guelph, Ontario, NIG 2W1, Canada; 15 Tāwharau Ora-School of Veterinary Science, Massey University, Palmerston North 4474, New Zealand; 16 School of Biodiversity, One Health & Veterinary Medicine, University of Glasgow, Glasgow, G61 1QH, Scotland; 17 Bern Center for Precision Medicine and Cancer Therapy Resistance Cluster, Department for BioMedical Research, University of Bern, 3008 Bern, Switzerland

## Abstract

Cancer is a common cause of morbidity and mortality in domestic cats. As the mutational landscape of domestic cat tumors remains uncharacterized, we performed targeted sequencing of 493 feline tumor-normal tissue pairs from 13 tumor types, focusing on the feline orthologs of ~1,000 human cancer genes. *TP53* was the most frequently mutated gene, and the most recurrent copy number alterations were loss of *PTEN* or *FAS*, or gain of *MYC*. By identifying 31 driver genes, mutational signatures, viral sequences, and tumor-predisposing germline variants, our study provides insight into the domestic cat oncogenome. We demonstrate key similarities with the human oncogenome, confirming the cat as a valuable model for comparative studies, and identify potentially actionable mutations, aligning with a ‘One Medicine’ approach.

## Introduction

Neoplastic disease is the leading cause of morbidity and mortality in companion animals, in particular pet cats and dogs (1), and their tumors share marked clinicopathologic similarities with human tumors (2). Notably, studying companion animal tumors offers several key advantages over using rodent models: Pets are exposed to the same environmental conditions as their owners, develop similar non-neoplastic related co-morbidities, such as diabetes (3, 4) and cardiovascular disease (5), and most relevantly, tumors arise spontaneously in a naturally heterogeneous population (2, 6, 7). Thus, cross-species comparisons have a key role in advancing precision medicine to improve the survival of both humans and their animal companions. This is in line with a ‘One Medicine’ approach (6, 8, 9), which promotes the two-way flow of data and knowledge between medical and veterinary disciplines to benefit both human and animal health (10).

Over the past decade, molecular characterization of canine tumors has grown exponentially, and extensive investment has been made in molecular biomarker discovery efforts (11). In addition, there is preliminary evidence that molecular analysis of tumors using targeted next-generation sequencing (NGS) panels may aid in the clinical management of canine cancer patients by providing information on prognosis and/or potential therapeutic options (12–14), with a vision to follow the trajectory of how genomics-based management has transformed human oncology. In contrast, less than a handful of NGS investigations have been published on feline neoplastic disease, all using small sample numbers and single tumor types (15–17). Thus, understanding the feline oncogenome could facilitate the development of diagnostic and prognostic biomarkers, targeted therapies, and nominate agents for repurposing in feline patients. Ultimately, this has the potential to improve the clinical management of feline cancer patients, and in a comparative oncology setting, this could also benefit human patients.

In this work, we performed targeted sequencing of 493 feline tumor-normal tissue pairs from 13 tumor types, assessing the mutational status of the feline orthologs of ~1,000 human cancer genes and comparing our findings to human counterparts. This study provides insights into the oncogenome of the domestic cat. In addition, we identified strong similarities to the human oncogenome and revealed several potentially actionable mutations.

## Results

### Demographics of the feline pan-cancer mutational landscape study

We selected tumor types that align with broad human histopathological classifications, encompassing both benign and highly aggressive malignant tumors, as well as common and rare tumor entities. The inclusion of uncommon or rare tumors was intentional: if cross-species comparisons reveal strong genetic similarities to their well-characterized human counterparts, such findings could identify potential therapeutic avenues for affected cats.

**Fig. 1:**
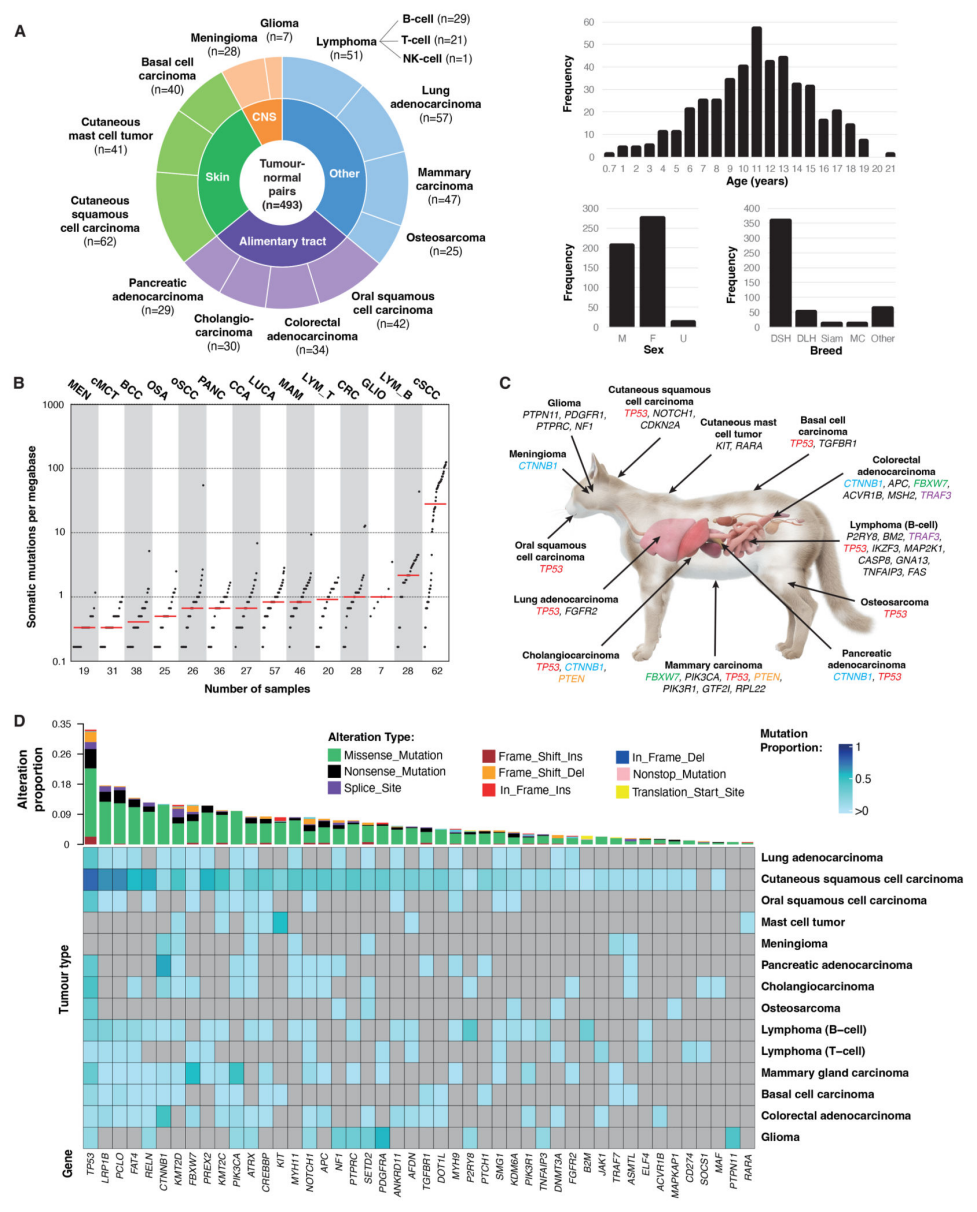
Overview of somatic mutational landscape of 13 feline tumor types. (**A**) Distribution of the tumor-normal samples based on tumor type (pie chart), and frequency of the age, sex, and breed of cats in the study cohort (bar plots). CNS, central nervous system; F, female; MC, Maine Coon; M, male; NK cell, natural killer cell; Siam, Siamese; U, unknown. (**B**) Distribution of the tumor mutational burden (TMB) in each tumor type. Each dot represents a sample, and the black horizontal line indicates the median TMB of the respective tumor type (only samples with >0 mutations that passed variant filtering are shown). LYM_Bcell, B-cell lymphoma; LYM_T-cell, T-cell lymphoma. (**C**) Genes identified as being under positive selection pressure in each tumor type (driver genes). Within each tumor type, the driver genes are listed in order of decreasing significance (q-global < 0.1) and those shown in color are present in more than one tumor type. The driver genes shown for LYM are for the B-cell subtype, because no driver genes were identified for the T-cell subtype. (**D**) Oncoplot of the top 5 most frequently mutated genes from each tumor type. Mutations include SNVs, multinucleotide variants (MNVs) and small insertions/deletions (indels; <100bp).

The study group consisted of primary tumors from 13 histologically defined tumor types; basal cell carcinoma (BCC, n=40 cases), cholangiocarcinoma (CCA; intrahepatic, n=30), colorectal adenocarcinoma (CRC, n=34), cutaneous mast cell tumor (cMCT, n=41), cutaneous squamous cell carcinoma (cSCC, n=62), glioma (GLIO, n=7), lung adenocarcinoma (LUCA, n=57), lymphoma (LYM, n=51), mammary carcinoma (MAM, n=47), meningioma (MEN, n=28), osteosarcoma (OSA, n=25), oral squamous cell carcinoma (oSCC, n=42), and pancreatic adenocarcinoma (PANC, n=29; Fig. 1A, Table S1(18)). Some cohorts could be subdivided by phenotype or location, specifically LYM (T- or B-cell), MAM (ER+ or ER-), and OSA (appendicular or axial).

The study population included more females than males (56% vs. 42%, respectively Fig. 1A), primarily due to the MAM cohort being entirely female. The tumor distribution was primarily towards older cats (with a median age of 11 years, range: 0.7 – 21 years; Fig. 1A, Table S1), consistent with cancer registries recording 8-11 years as the mean age of tumour presentation (depending on the country) (19–22). The most common breed was Domestic Shorthair (DSH; a general term for a non-pedigreed cat with a short coat; 73.1%), followed by Domestic Longhair (DLH; 10.4%), Siamese (2.2%), and Maine Coon (1.8%), with the “Others” category composed of 24 different breeds (n=1 to 10 cats each; Fig. 1A). All sample details, including signalment data, are provided in Table S2 (18).

### The somatic mutational landscape across cancer types

To provide an investigation of cancer-associated genes that may be altered in feline tumors, we compiled a list of 1,039 human cancer-associated genes (see materials and methods) and identified their feline orthologs; ultimately, 978 feline genes were included in our targeted panel. Tissue samples were from the original diagnostic formalin-fixed, paraffin-embedded (FFPE) blocks, with the diagnosis independently verified by a board-certified veterinary pathologist, who then selected the tumor and normal areas for sampling. Genomic DNA extracted from the 493 tumor-normal tissue pairs was hybridized with the targeted panel, and the captured DNA was subsequently analyzed by NGS. To gain an overview of the feline somatic mutational landscape, we first identified the somatic mutations in each tumor, including somatic single nucleotide variants (SNVs), multi-nucleotide variants, and insertions/deletions, relative to the FelCat9 reference assembly (Table S3 (18) lists the protein-altering variants in each sample). We have also provided the variant locations relative to the Fca126 reference assembly (Table S3 (18)

The tumor mutational burden varied across the tumor types, with a median of ~0.5 to ~20 mutations/megabase (Fig. 1B). Mutational signature analysis identified COSMIC Signature SBS7 in 34/62 (52%) of the cSCC samples (Table S4 (18)). The proposed etiology of SBS7 is ultraviolet (UV) light exposure (23), with this signature predominantly found in UV-associated human skin cancers.

Across the study, we identified 31 driver genes (Fig. 1C, Table S5 (18)). Five genes, *TP53, CTNNB1, PTEN, TRAF3*, and *FBXW7*, were drivers in multiple tumor types, whereas most were only drivers of a specific tumor type, such as *KIT* for cMCT and *PIK3CA* for MAM. To gain insights into the comprehensiveness of our targeted sequencing approach for driver gene identification, we performed whole-exome sequencing (WES) of freshly collected feline MAM (n=18; Fig. S1, Table S3 (18)), finding *FBXW7* and *PIK3CA* as drivers, in keeping with results from targeted sequencing of our MAM cohort (Table S5 (18)). Across the entire pan-cancer cohort, *TP53* was the most recurrently mutated gene (Fig. 1D), and 14 mutation hotspots in 10 genes were identified (Table S6 (18)). The most frequently observed were *PIK3CA* p.H1047R, found in 19/493 (4%) tumors, most commonly occurring in MAM (30% of the cohort). To rule out the possibility that recurrent mutations were artifacts, somatic hotspot mutations in the MAM and cMCT cohorts were orthogonally validated with capillary sequencing (Fig. S2).

**Fig. 2:**
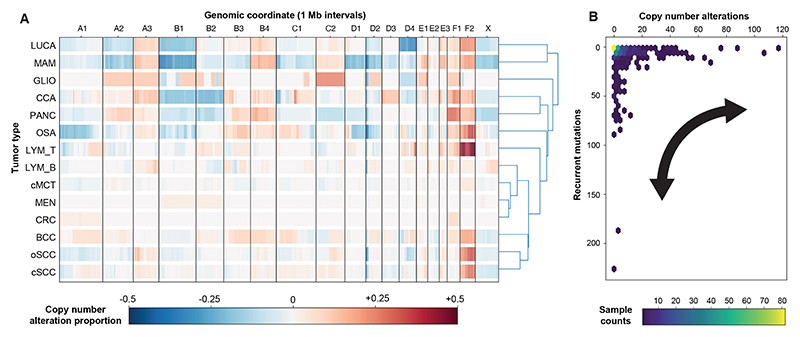
Overview of somatic CNA landscape of 13 feline tumor types. (**A**) Somatic CNAs across the 13 tumor types. CN gains/amplifications [log_2_ (fold change) ≥ + 0.32] are shown in red and losses/deletions [log_2_(fold change) ≤ -0.4] are in blue. The color shown is the proportion of tumors (0-40% of the cohort) showing an overall CN gain or loss at 1 Mb intervals across the feline genome (chromosomes indicated on the x-axis). Tumor types were hierarchically clustered using average linkage and Euclidean distance on their net gain/loss profiles to define the dendrogram structure. (**B**) Hexagon-binned density plot showing the approximate inverse relationship between focal CNA burden (x-axis) and recurrent mutation burden (y-axis). CNAs were counted as focal events ≤ 10 Mb with log_2_ (fold change) ≥ +0.32 (gain) or ≤ −0.40 (loss), summed per sample. Recurrent mutations were counted per sample and restricted to genes mutated in ≥ 2 samples across the cohort. Truncating variants (nonsense, frameshift indel, splice-site, start-loss, nonstop) were always included. Missense and in-frame indels were included for non-hypermutators; for hypermutator tumors (considered as those with an SBS7 signature) they were counted only when occurring in hotspot events, i.e. the same amino-acid position mutated in ≥ 2 tumors with a ±1-residue tolerance. Hexagon colour encodes the number of samples in each bin; only samples with both mutation and CNV data were included.

As somatic copy number alterations (CNA) drive many human tumor types, it was important to characterize the CNA landscape of the feline tumors. On average, MAM showed the most CNAs (mean genome fraction altered: 16.94%, range: 0.00-66.72%, and CRC showed the least (mean: 0.59%, range: 0.00 to 9.88%; Fig. 2A). Recurrent whole chromosome gains and losses were seen, with eleven tumor types showing gain of chromosomes F1 or F2, and nine showing loss of chromosome X (Table S7 (18)). Considering both broad (≥10 Mb up to a whole chromosome) and focal (<10 Mb) CNA across the feline tumor types, copy number (CN) gains were consistently seen on chromosomes A3, B4, F1, and F2. The gene most altered by CN gain was *MYC* (20% all tumors), particularly noticeable in T-cell LYM (57% of cases). CN loss at the start of chromosome D2 was seen in all tumor types except CRC. The genes most affected by CN loss were *PTEN, FAS* (~20% all tumors), and *CDKN2A* (15% all tumors). Genes within each of the broad and focal gains/losses are shown in Fig. S3. When considering both the CNAs and somatic mutations within each sample, we observed an inverse relationship, particularly in the highly altered tumors (Fig. 2B); such tumors were replete with either CNAs or mutations, but rarely both, which is consistent with the ‘cancer genome hyperbola’ of human tumors (24). The samples with the highest recurrent mutations were predominantly found in the cSCC cohort, whereas the samples with the highest CNAs came from various tumor types (Table S8 (18)).

Next, we examined the feline somatic mutational landscape on a per-tumor-type basis, to define the genetic landscape of each tumor type, which may shed light on their biological behavior. Complete descriptions of the somatic mutations and CNAs identified in each tumor type are included in the Supplementary Text, Figs. S4-S17 and Table S9 (18).

### Presence of papillomavirus in cSCC and BCC

Some feline cancers have been associated with viral infection, with the oncogenic potential of feline leukemia virus (FeLV), feline immunodeficiency virus (FIV) and papillomavirus (PV) having been well-established in cats (25, 26). Thus, to look for a possible viral etiology of the tumors in this study, we searched the off-target sequencing reads for the presence of viral DNA sequences (filtering out any reads for FIV or FeLV; see materials and methods). This approach identified strong evidence of two different genera of PV, specifically *Dyothetapapillomavirus* (*DyoPV*) and *Taupapillomavirus* (*TauPV*), in the cSCC and BCC samples, with only one oSCC sample showing the presence of *TauPV* sequences (Fig. S18).

### Germline predisposition variants

Sequencing of matched normal tissue for each tumor enabled the identification of putative pathogenic germline variants that may have predisposed these cats to cancer. We considered loss-of-function variants and known human cancer predisposition genes (see materials and methods). With this approach we identified variants in 14 genes (Table S10A (18)). Of the 493 cats in this study, 20 had at least one putative cancer-predisposition germline variant, with only one cat having more than one variant. The median age at diagnosis for these 20 cats was 11 ± 3.7 years (SD), which was not significantly different from cats without one of these variants (*P*=0.5671; two-sided Wilcoxon rank-sum test). *CHEK2* was the only gene to have recurrent variants in >2 samples. One of the *CHEK2* variants (c.546C>A; p.Y182*), found in 4 cats, mapped to an orthologous site in humans, which is annotated in the ClinVar database as a pathogenic allele in humans (Table S10B (18)). Similarly, additional orthologous variants in *CHEK2* and *BRIP1* (n=1 each) have been reported in the ClinVar database as pathogenic (Table S10B (18)).

### Cross-species comparison with mutations found in human cancers

**Fig. 3:**
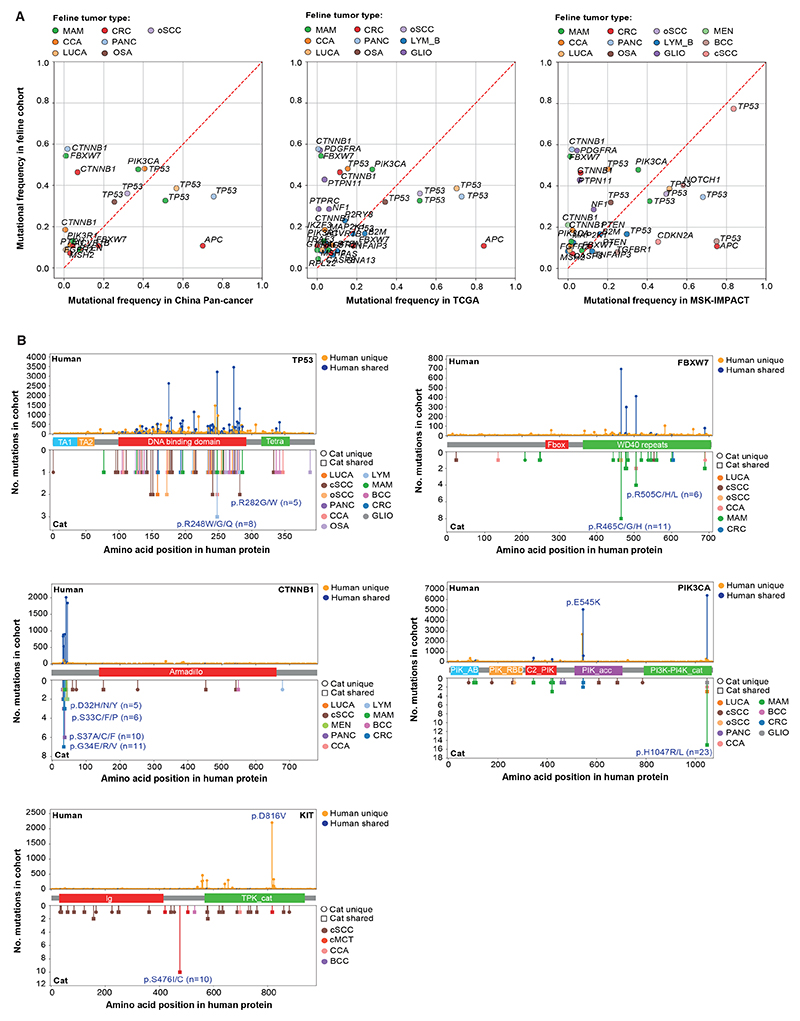
Cross-species comparison of the somatic oncogenomic mutational landscape between humans and felines. (**A**) Mutation frequencies of the feline driver genes in the feline cohort (y-axis) versus the human cohort (‘China Pan-cancer’, TCGA’ or ‘MSK-IMPACT’; x-axis) per tumor type. The red dotted line indicates the null hypothesis (no difference in mutational frequency between the species). (**B**) Comparison of the feline and human hotspots. Lollipop plots depict the mutational distribution in *TP53, FBXW7, CTNNB1, PIK3CA*, and *KIT* in human tumors (upper panel; obtained from the COSMIC database) and cat tumors from this study (lower panel). Between panels the human protein with relevant domains (based on the canonical transcript as defined by Ensembl v104) is indicated. Domain information obtained from the SMART or Pfam databases. C2_PIK, C2 phosphatidylinositol 3-kinase-type domain; Ig, immunoglobulin subtype; PIK_AB, phosphatidylinositol 3-kinase, adaptor-binding domain; PI3K-PI4K_cat, phosphatidylinositol 3-/4-kinase, catalytic domain; PIK_acc; phosphoinositide 3-kinase, accessory (PIK) domain; PIK_RBD, phosphatidylinositol 3-kinase Ras-binding (PI3K RBD) domain; PTK_cat, tyrosine-protein kinase, catalytic domain; TA, transactivation domain; Tetra, tetramerization domain.

We first compared the mutational frequency of feline driver genes within tumors of the same type from human pan-cancer datasets (Table S11 (18)). Only genes analyzed in both datasets were considered (Fig. 3A). In some cases, the genes showed similar tumor type mutational frequencies between the species, such as *PIK3CA* in MAM, and *TP53* in OSA, oSCC and MAM. Conversely, in some cases, the mutational frequencies were very different, such as *APC* in CRC, *CTNNB1* in PANC and *FBXW7* in MAM. A cross-species comparison of the mutational landscape of each tumor type, including somatic mutations and CNAs, is included in the Supplementary Text.

We next compared the frequency and position of mutations in five key driver genes identified in this study with those in human cancers by ‘humanizing’ cat mutations (see materials and methods). Shared recurrent mutations were seen in *TP53*, predominately in the DNA binding domain; *FBXW7*, in the WD40 repeats; and *CTNNB1*, in the N-terminus (amino acids 32-37) - a region critical for stabilization of the protein (27) (Fig. 3B). However, while p.H1047R/L in the catalytic domain of *PIK3CA* was highly recurrent in both species, p.E545K in the accessory domain was far more recurrent in human cancers than feline cancers. In addition, *KIT* p.D816V was only present in human cancers. Additional shared recurrent mutations may be identified as other cat tumor types are sequenced.

### Actionability of feline driver gene mutations

We next sought to use our catalog of feline driver mutations for the benefit of cats by leveraging human tractability and druggability databases. Although this knowledge is absent for cat proteins, candidate treatments may be identified for proteins with a high level of homology with the human counterpart. This reasoning is supported by data from dogs that have tumors with specific genomic alterations and show improved outcomes when treated with human-targeted treatments (13, 14), and evidence of biological activity of the canine-approved tyrosine kinase inhibitor (TKI) toceranib, in cats with *KIT*-mutated MCTs (28, 29).

**Fig. 4:**
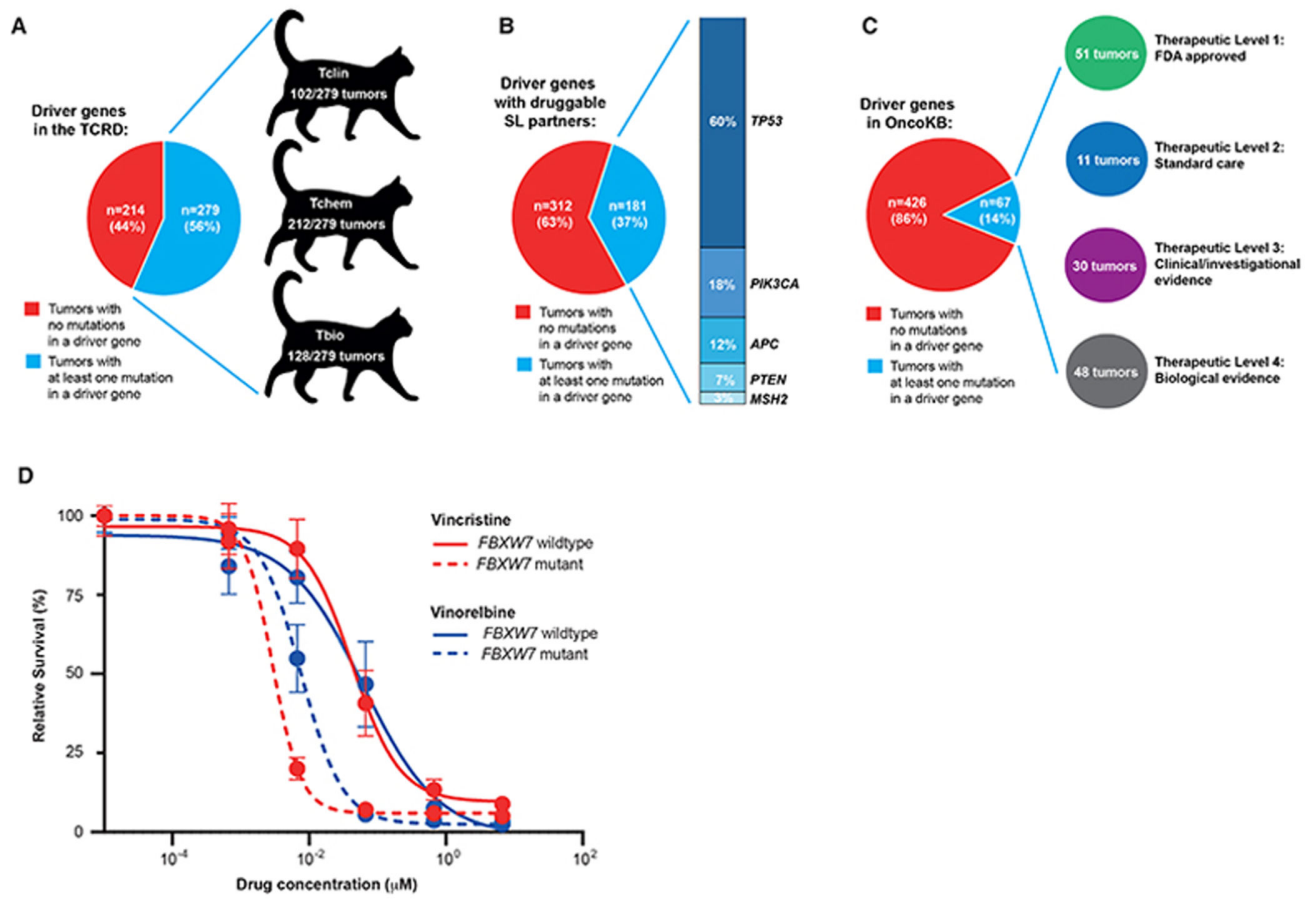
Actionability of feline driver gene mutations. (**A**) The proportion of feline tumors that had a mutation in at least one of the feline orthologs of driver genes present in the Target Central Resource Database (TCRD) and the number of feline tumors with a mutated driver gene where the human ortholog has a Pharos Target Development Ranking (Tclin, Tchem, or Tbio). ‘Tclin’ encode proteins for which an approved drug exists, ‘Tchem’ encode proteins not in Tclin but known to bind small molecules with high potency, and ‘Tbio’ encode proteins with a moderate amount of available data (including literature references, GeneRIF annotations, experimental data). (**B**) The proportion of feline tumors that have a mutation in at least one driver gene with a SL partner that is druggable, and the frequency of the mutation of these driver genes (*TP53, PIK3CA, APC, PTEN*, and *MSH2*). (**C**) The proportion of feline tumors with an actionable mutation in at least one of the driver genes present in the OncoKB database, and the number of tumors with an actionable mutated driver gene within each therapeutic category (Level 1-4). ‘Level 1’ therapeutic biomarkers represent US Food and Drug Administration (FDA)-recognized biomarkers predicted to respond to FDA-approved drugs, ‘Level 2’ therapeutic biomarkers represent standard care biomarkers that are recommended by professional guidelines to be predictive of response to FDA-approved drugs, ‘Level 3’ therapeutic biomarkers show compelling clinical evidence, standard care or investigational evidence of a response to an FDA-approved or investigational drug, and ‘Level 4’ therapeutic biomarkers show compelling biological evidence of being predictive of response to a drug. **(D)** Dose–response curves comparing vincristine and vinorelbine sensitivity between *FBXW7*-mutant and wildtype tumoroids are shown, with error bars representing 95% confidence intervals (CI). Experiments were performed using three independent tumoroid lines (per genotype) representing biological replicates. For each line, two independently cultured experiments were performed, and each condition was assayed in technical triplicate. For vincristine, the estimated IC_50_ was 0.04226 μM in wildtypes, compared with 0.002895 μM in mutants. For vinorelbine, the IC_50_ was 0.06163 μM and 0.007452 μM in wildtypes and mutants, respectively. An extra sum-of-squares *F* test indicated that the dose–response curves differed significantly between wildtype and mutant groups for both drugs (p < 0.0001).

We first searched a database of protein druggability predictions (30) and found 6/31 (19%) driver genes encoded proteins for which an approved drug exists (‘Tclin’), with 102/493 (21%) tumors having mutations in these driver genes (Fig. 4A, Table S12A (18)). Next, we searched a database of validated cancer synthetic lethal (SL) targets (31) and found 5/31 driver genes had druggable SL partners, with 181/493 (37%) tumors having mutations in these driver genes (Fig. 4B, Table S12B (18)). Finally, we searched the OncoKB actionability database (32), specifically using ‘humanized’ feline mutations within the driver genes (SNVs only; see materials and methods), and found 67/493 (14%) tumors had an oncogenic/likely oncogenic mutation in at least one of the actionable driver genes (specifically, *PIK3CA, MAP2K1, KIT, FBXW7, FGFR2, PTEN*, and *NF1*; Fig. 4C, Table S12C (18)). As a further proof-of-concept we used patient-derived three dimensional (3D) feline MAM tumoroids to investigate whether *FBXW7* mutations create a genotype-specific vulnerability (tumoroids details are in Table S2). After WES of tumoroids, six lines were selected; three *FBXW7* wildtype lines and three *FBXW7* mutant lines (Table S3 (18), Fig. S19). *FBXW7*-mutant lines were significantly more sensitive to the vinca alkaloids, vincristine and vinorelbine (Fig. 4D, Table S13 (18)), consistent with a previous *in vitro* study using human HAP1 cells (33); a result recommending further investigation in larger cohorts.

## Discussion

Despite cats being the second most frequent companion animal in households across many countries, and neoplastic disease being one of their leading causes of morbidity and mortality, there is a dearth of studies investigating the genetics of feline tumors. Thus, cancer gene mutational profiling of 493 feline tumor-normal pairs from 13 tumor types offers important insights into the feline oncogenome.

*TP53* was the most frequently mutated gene in this feline pan-cancer study (33% of all tumors), mirroring reports in human pan-cancer studies (34% of all tumors) (34). Similarly, the most recurrent CNAs identified in the feline tumors were gain of *MYC* and loss of *PTEN/FAS* (each observed in 20% tumors). Consistently, human pan-cancer analyses have reported frequent *MYC* amplification (28% of cancers; (35)) and *PTEN* hemizygous deletion (25% of cancers; (36)). By contrast, *RAS* gain-of-function mutations are found in ~25% of human cancers (37), whereas *RAS* was not a driver in any feline tumor types in our study, and feline hotspot mutation studies have found *RAS* mutations to be uncommon (38–42).

Consistent with previous accounts of FcaPV2 (*DyoPV*) and FcaPV3/4/6 (*TauPV*) DNA being present in feline cSCC (43), and FcaPV3/5 DNA being present in feline BCC (44, 45), we observed *DyoPV* and *TauPV* DNA sequence in some cSCC and BCC samples. However, we also observed these sequences in some of the normal samples, and most feline PV infections do not result in neoplasia (43), consistent with human PV infections (46). It is currently poorly understood why PV infections only result in neoplasia in some cats, with additional co-factors proposed to be required to induce transformation, such as UV exposure (43); notably, 25% of our cSCC with PV DNA sequences present also showed a UV mutational signature.

To-date, only one cancer predisposition allele has been reported in cats (a single nucleotide polymorphism in *TP53* intron 7 in feline injection-site sarcoma) (47), although this was not replicated in a second study (48). We identified 14 putative pathogenic germline variants that may predispose these cats to cancer. Notably, the limited catalog of feline germline variants that have been collected (49), and the genetic distance between the cats we sequenced and the Abyssinian reference genome (50), will require further validation of these candidate predisposing alleles.

Because several tumor types harbored recurrent, potentially actionable mutations, we considered the potential clinical utility of genomic profiling in feline cancers. *FBXW7* is a driver gene of feline MAM (mutated in 53 to 72% of cases) and the combination therapy of lunresertib with camonsertib is in human clinical trials for cancers with specific genomic alterations, including *FBXW7* mutations (MYTHIC Study, NCT04855656). *PIK3CA* is also a feline MAM driver gene (mutated in ~45% of cases) and there are PI3K inhibitors available, such as combinations of alpelisib with fulvestrant or capivasertib with fulvestrant, approved for treating *PIK3CA*-mutated breast cancer in humans. These drug combinations may hold translational promise in feline oncology pending further evaluation. Finally, because *KIT* is a driver gene of feline cMCT (mutated in ~40% of cases), screening feline cMCT cases for the presence of oncogenic *KIT* mutations may be of clinical value, particularly because the canine-approved TKI toceranib, with a wide variety of molecular targets, including KIT, is well tolerated in cats and clinical responses have been reported in feline cMCT (28, 29). Overall, our findings support the value of genomic characterization in feline cancers, particularly MAM and cMCT, both for identifying druggable alterations and for predicting therapeutic responses, as demonstrated through our functional validation using tumoroids.

By sequencing orthologs of ~1000 known human cancer genes, we limited the discovery of driver genes in feline cancer to those in human cancers. Nevertheless, this study offers insight into the feline oncogenome, identifying strong similarities to the driver events in human cancers and providing several potentially actionable mutations for further investigation. This should serve as a valuable resource for veterinarians and comparative oncologists, providing a step towards precision oncology for our feline companions.

## Supplementary Material

Supplementary Text, Materials & Methods, Figures S1-S17 and legends for Supplementary Tables S1-S18

## Data Availability

The raw sequencing data for each cohort are available for download from the European Nucleotide Archive (ENA; https://www.ebi.ac.uk/ena/browser/home) under study accessions ERP143204 (feline BCC), ERP145946 (feline CCA), ERP141603 (feline CRC), ERP133248 (feline cMCT), ERP133244 (feline cSCC), ERP141601 (feline GLIO), ERP165333 (feline LUCA), ERP141159 (feline LYM), ERP141158 (feline MAM), ERP136468 (feline MEN), ERP133245 (feline oSCC), ERP143495 (feline osteosarcoma), ERP137019 (feline PANC), and ERP162973 (WES of feline MAM and tumoroids). All code is archived within Zenodo (as detailed in the Materials and Methods), and links to individual analyses are collated in GitHub (51). Requests for the feline mammary carcinoma tumoroids can be addressed to Prof. Sven Rottenberg.

## References

[1] Sarver AL, Makielski KM, DePauw TA, Schulte AJ, Modiano JF (2022). Increased risk of cancer in dogs and humans: a consequence of
recent extension of lifespan beyond evolutionarily-determined
limitations?. Aging Cancer.

[2] Cannon CM (2015). Cats, Cancer and Comparative Oncology. Veterinary Sciences.

[3] Henson MS, O’Brien TD (2006). Feline models of type 2 diabetes mellitus. Ilar j.

[4] Osto M, Zini E, Reusch CE, Lutz TA (2013). Diabetes from humans to cats. Gen Comp Endocrinol.

[5] Liu SK, Roberts WC, Maron BJ (1993). Comparison of morphologic findings in spontaneously occurring
hypertrophic cardiomyopathy in humans, cats and dogs. Am J Cardiol.

[6] Oh JH, Cho J-Y (2023). Comparative oncology: overcoming human cancer through companion
animal studies. Experimental & Molecular Medicine.

[7] Garden OA, Volk SW, Mason NJ, Perry JA (2018). Companion animals in comparative oncology: One Medicine in
action. Vet J.

[8] Cardiff RD, Ward JM, Barthold SW (2008). ‘One medicine---one pathology’: are veterinary and
human pathology prepared?. Lab Invest.

[9] Nance RL, Sajib AM, Smith BF, Tao Y-X (2022). Progress in Molecular Biology and Translational Science.

[10] King TA (2021). The One Medicine concept: its emergence from history as a
systematic approach to re-integrate human and veterinary
medicine. Emerging Topics in Life Sciences.

[11] Aupperle-Lellbach H, Kehl A, de Brot S, van der Weyden L (2024). Clinical Use of Molecular Biomarkers in Canine and Feline
Oncology: Current and Future. Vet Sci.

[12] Chon E (2023). Novel genomic prognostic biomarkers for dogs with
cancer. J Vet Intern Med.

[13] Chon E (2023). Genomic tumor analysis provides clinical guidance for the
management of diagnostically challenging cancers in dogs. J Am Vet Med Assoc.

[14] Wu K (2023). Analyses of canine cancer mutations and treatment outcomes using
real-world clinico-genomics data of 2119 dogs. NPJ Precis Oncol.

[15] Wong K (2021). Comparison of the oncogenomic landscape of canine and feline
hemangiosarcoma shows novel parallels with human
angiosarcoma. Dis Model Mech.

[16] Wong K (2023). Cross-species oncogenomics offers insight into human
muscle-invasive bladder cancer. Genome Biol.

[17] Rodney AR (2023). Genomic landscape and gene expression profiles of feline oral
squamous cell carcinoma. Front Vet Sci.

[18] van der Weyden L, Adams DJ (2025).

[19] Graf R (2015). Swiss Feline Cancer Registry: A Retrospective Study of the
Occurrence of Tumours in Cats in Switzerland from 1965 to
2008. J Comp Pathol.

[20] Pérez Enriquez J, Romero-Romero L, Alonso Morales R, Fuentes-Pananá E (2020). Tumor prevalence in cats: experience from a reference diagnostic
center in Mexico City (2006-2018). Veterinaria México OA.

[21] Pinello K (2022). Vet-OncoNet: Malignancy Analysis of Neoplasms in Dogs and
Cats. Veterinary Sciences.

[22] Seung BJ, Bae MK, Sur JH (2024). Regional Variations in and Key Predictors of Feline Tumor
Malignancy: A Decade-Long Retrospective Study in Korea. Animals (Basel).

[23] Alexandrov LB (2020). The repertoire of mutational signatures in human
cancer. Nature.

[24] Ciriello G (2013). Emerging landscape of oncogenic signatures across human
cancers. Nat Genet.

[25] Parisi F (2023). Exploring the link between viruses and cancer in companion
animals: a comprehensive and comparative analysis. Infect Agent Cancer.

[26] Munday JS, Knight CG (2024). Papillomaviruses and Papillomaviral Disease in Dogs and Cats: A
Comprehensive Review. Pathogens.

[27] Shah K, Kazi JU (2022). Phosphorylation-Dependent Regulation of WNT/Beta-Catenin
Signaling. Front Oncol.

[28] Berger EP (2018). Retrospective evaluation of toceranib phosphate (Palladia) use in
cats with mast cell neoplasia. Journal of Feline Medicine and Surgery.

[29] Harper A, Blackwood L (2017). Toxicity and response in cats with neoplasia treated with
toceranib phosphate. J Feline Med Surg.

[30] Kelleher KJ (2023). Pharos 2023: an integrated resource for the understudied human
proteome. Nucleic Acids Res.

[31] Schäffer AA, Chung Y, Kammula AV, Ruppin E, Lee JS (2024). A systematic analysis of the landscape of synthetic
lethality-driven precision oncology. Med.

[32] Suehnholz SP (2024). Quantifying the Expanding Landscape of Clinical Actionability for
Patients with Cancer. Cancer Discovery.

[33] Gerhards NM (2018). Haploid genetic screens identify genetic vulnerabilities to
microtubule-targeting agents. Mol Oncol.

[34] (2020). Pan-cancer analysis of whole genomes. Nature.

[35] Schaub FX (2018). Pan-cancer alterations of the MYC oncogene and its proximal
network across the cancer genome atlas. Cell systems.

[36] Vidotto T (2023). Pan-cancer genomic analysis shows hemizygous PTEN loss tumors are
associated with immune evasion and poor outcome. Sci Rep.

[37] Hobbs GA, Der CJ, Rossman KL (2016). RAS isoforms and mutations in cancer at a glance. J Cell Sci.

[38] Watzinger F, Mayr B, Gamerith R, Vetter C, Lion T (2001). Comparative analysis of ras proto-oncogene mutations in selected
mammalian tumors. Mol Carcinog.

[39] Mayr B, Winkler G, Schaffner G, Reifinger M, Brem G (2002). N-ras mutation in a feline lymphoma. Low frequency of N-ras
mutations in a series of feline, canine and bovine lymphomas. Vet J.

[40] Mayr B, Schaffner G, Reifinger M, Loupal G (2003). K-ras protooncogene mutations in feline pancreatic
adenocarcinomas. Vet Rec.

[41] Crozier C (2016). KRAS Mutations in Canine and Feline Pancreatic Acinar Cell
Carcinoma. J Comp Pathol.

[42] Kuroki K (2024). Hotspot Exon 15 Mutations in BRAF Are Uncommon in Feline
Tumours. Vet Comp Oncol.

[43] Munday JS, Thomson NA (2021). Papillomaviruses in Domestic Cats. Viruses.

[44] Munday JS, French A, Thomson N (2017). Detection of DNA sequences from a novel papillomavirus in a
feline basal cell carcinoma. Vet Dermatol.

[45] Munday JS, Gedye K, Knox MA, Pfeffer H, Lin X (2023). Genetic characterisation of Felis catus papillomavirus type 7, a
rare infection of cats that may be associated with skin
cancer. Vet Microbiol.

[46] Plummer M (2016). Global burden of cancers attributable to infections in 2012: a
synthetic analysis. The Lancet Global Health.

[47] Banerji N, Kapur V, Kanjilal S (2007). Association of germ-line polymorphisms in the feline p53 gene
with genetic predisposition to vaccine-associated feline
sarcoma. J Hered.

[48] Mucha D, Laberke S, Meyer S, Hirschberger J (2014). Lack of association between p53 SNP and FISS in a cat population
from Germany. Vet Comp Oncol.

[49] Buckley RM (2020). A new domestic cat genome assembly based on long sequence reads
empowers feline genomic medicine and identifies a novel gene for
dwarfism. PLoS Genet.

[50] Pontius JU (2007). Initial sequence and comparative analysis of the cat
genome. Genome Res.

[51] Billington JA (2025). GitHub.

[52] Kamstock DA (2011). Recommended guidelines for submission, trimming, margin
evaluation, and reporting of tumor biopsy specimens in veterinary surgical
pathology. Vet Pathol.

[53] Klang A (2025). Feline eosinophilic sclerosing fibroplasia associated with
T-/natural killer-cell lymphoma. Vet Pathol.

[54] Makhlouf S (2023). The Clinical and Biological Significance of Estrogen Receptor-Low
Positive Breast Cancer. Modern Pathology.

[55] Zehir A (2017). Mutational landscape of metastatic cancer revealed from
prospective clinical sequencing of 10,000 patients. Nat Med.

[56] Sondka Z (2018). The COSMIC Cancer Gene Census: describing genetic dysfunction
across all human cancers. Nature Reviews Cancer.

[57] Vogelstein B (2013). Cancer genome landscapes. Science.

[58] Li H (2013). Aligning sequence reads, clone sequences and assembly contigs
with BWA-MEM. arXiv Preprint.

[59] Tischler G, Leonard S (2014). biobambam: tools for read pair collation based algorithms on BAM
files. Source Code for Biology and Medicine.

[60] Danecek P (2021). Twelve years of SAMtools and BCFtools. Gigascience.

[61] Jones D (2016). cgpCaVEManWrapper: Simple Execution of CaVEMan in Order to Detect
Somatic Single Nucleotide Variants in NGS Data. Curr Protoc Bioinformatics.

[62] Raine KM (2015). cgpPindel: Identifying Somatically Acquired Insertion and
Deletion Events from Paired End Sequencing. Curr Protoc Bioinformatics.

[63] Adams DJ (2025). FUR Caveman and Pindel References.

[64] Billington JA, Francis BA (2025). Justaphase (v020).

[65] Martin M (2016). WhatsHap: fast and accurate read-based phasing. bioRxiv.

[66] CASM_Cancer_IT (2021). CASM-Smart-Phase.

[67] Quinlan AR, Hall IM (2010). BEDTools: a flexible suite of utilities for comparing genomic
features. Bioinformatics.

[68] Billington JA, Vermes I, Francis BA (2025). FUR-phaser (v092).

[69] McLaren W (2016). The Ensembl Variant Effect Predictor. Genome Biol.

[70] Wong K, Billington JA, Vermes I (2025). QC.

[71] Broad_Institute (2019). Picard Toolkit.

[72] Wong K, Billington JA, Vermes I (2025). MAF.

[73] Kandoth C (2020). vcf2maf (v16).

[74] Mayakonda A, Lin DC, Assenov Y, Plass C, Koeffler HP (2018). Maftools: efficient and comprehensive analysis of somatic
variants in cancer. Genome Res.

[75] Alexandrov_Lab (2020). TMB_plotter.

[76] Francis BA, Billington JA, Vermes I (2025). MAF_Updater.

[77] Diesh C (2023). JBrowse 2: a modular genome browser with views of synteny and
structural variation. Genome Biology.

[78] Francis BA, Vermes I, Billington JA (2025). fur_cnvkit.

[79] Talevich E, Shain AH, Botton T, Bastian BC (2016). CNVkit: Genome-Wide Copy Number Detection and Visualization from
Targeted DNA Sequencing. PLoS Comput Biol.

[80] Chandramohan R (2022). A Validation Framework for Somatic Copy Number Detection in
Targeted Sequencing Panels. J Mol Diagn.

[81] Gori K, Baez-Ortega A (2020). sigfit: flexible Bayesian inference of mutational
signatures. bioRxiv.

[82] Martincorena I (2017). Universal Patterns of Selection in Cancer and Somatic
Tissues. Cell.

[83] Martincorena I (2017). Using dNdScv in a different species or assembly.

[84] Francis BA, Billington JA (2025). Domestic Cat Oncogenome SMG Analysis.

[85] Billington JA, Del Castillo Velasco-Herrera M, Wong K (2025). dermatlas_germlinepost_nf.

[86] Billington JA, Francis BA (2025). pathogen_identification.

[87] Langmead B (2023). Kraken 2, KrakenUniq and Bracken indexes.

[88] Francis BA, Vermes I (2025). project_fur_felis_catus_publication_plots.

[89] Wellcome_Sanger_Institute COSMIC.

[90] cBioPortal Mutation Mapper (v641).

[91] Cerami E (2012). The cBio cancer genomics portal: an open platform for exploring
multidimensional cancer genomics data. Cancer Discov.

[92] cBioPortal (2020). Pan-cancer analysis of whole genomes (ICGC/TCGA. Nature.

[93] cBioPortal (2017). MSK-IMPACT Clinical Sequencing Cohort MSK. Nat Med.

[94] Wu L (2022). Landscape of somatic alterations in large-scale solid tumors from
an Asian population. Nat Commun.

[95] cBioPortal (2022). China Pan-cancer OrigiMed. Nature.

[96] Pharos Pharos.

[97] Sheils TK (2020). TCRD and Pharos 2021: mining the human proteome for disease
biology. Nucleic Acids Research.

[98] Chakravarty D (2017). OncoKB: A Precision Oncology Knowledge Base. JCO Precision Oncology.

[99] MSKCC OncoKB.

[100] Duarte AA (2018). BRCA-deficient mouse mammary tumor organoids to study cancer-drug
resistance. Nat Methods.

[101] Inglebert M (2022). A living biobank of canine mammary tumor organoids as a
comparative model for human breast cancer. Sci Rep.

[102] Goldschmidt MH, Pena L, Zappulli V (2016). Tumors in Domestic Animals.

[103] Mayr B (2000). Presence of p53 mutations in feline neoplasms. Res Vet Sci.

[104] Saif R (2016). Hspb1 and Tp53 Mutation and Expression Analysis in Cat Mammary
Tumors. Iran J Biotechnol.

[105] Ferreira D (2019). Gene expression association study in feline mammary
carcinomas. PLoS One.

[106] Borge KS (2015). Canine Mammary Tumours Are Affected by Frequent Copy Number
Aberrations, including Amplification of MYC and Loss of PTEN. PLoS One.

[107] Shahbandi A, Nguyen HD, Jackson JG (2020). TP53 Mutations and Outcomes in Breast Cancer: Reading beyond the
Headlines. Trends in Cancer.

[108] Thorpe LM, Yuzugullu H, Zhao JJ (2015). PI3K in cancer: divergent roles of isoforms, modes of activation
and therapeutic targeting. Nature Reviews Cancer.

[109] Lebok P (2015). Partial PTEN deletion is linked to poor prognosis in breast
cancer. BMC Cancer.

[110] Barrs VR, Beatty JA (2012). Feline alimentary lymphoma: 1. Classification, risk factors,
clinical signs and non-invasive diagnostics. J Feline Med Surg.

[111] Vezzali E, Parodi AL, Marcato PS, Bettini G (2010). Histopathologic classification of 171 cases of canine and feline
non-Hodgkin lymphoma according to the WHO. Vet Comp Oncol.

[112] Moore PF, Rodriguez-Bertos A, Kass PH (2012). Feline gastrointestinal lymphoma: mucosal architecture,
immunophenotype, and molecular clonality. Vet Pathol.

[113] Valli VE, Bienzle D, Meuten DJ (2016). Tumors in Domestic Animals.

[114] Valli V, Kiupel M, Bienzle D, Wood R, Maxie G (2015). Jubb, Kennedy and Palmer’s Pathology of Domestic Animals.

[115] Chino J (2013). Cytomorphological and immunological classification of feline
lymphomas: clinicopathological features of 76 cases. J Vet Med Sci.

[116] Eraghi V (2025). Feline Lymphoma in Focus: Examining the Patterns and Types in
Croatia’s Pathological Records. Vet Sci.

[117] Santagostino SF (2015). Feline upper respiratory tract lymphoma: site, cyto-histology,
phenotype, FeLV expression, and prognosis. Vet Pathol.

[118] Ponce F (2010). A morphological study of 608 cases of canine malignant lymphoma
in France with a focus on comparative similarities between canine and human
lymphoma morphology. Vet Pathol.

[119] Challa-Malladi M (2011). Combined genetic inactivation of β2-Microglobulin and CD58
reveals frequent escape from immune recognition in diffuse large B cell
lymphoma. Cancer Cell.

[120] Wang X (2018). Clinical Significance of PTEN Deletion, Mutation, and Loss of
PTEN Expression in De Novo Diffuse Large B-Cell Lymphoma. Neoplasia.

[121] Razzaghi R (2021). Compromised counterselection by FAS creates an aggressive subtype
of germinal center lymphoma. J Exp Med.

[122] Li P (2021). Genomic Mutation Profile of Primary Gastrointestinal Diffuse
Large B-Cell Lymphoma. Front Oncol.

[123] Xu-Monette ZY (2024). DNA mismatch repair defect and intratumor heterogeneous
deficiency differently impact immune responses in diffuse large B-cell
lymphoma. Oncoimmunology.

[124] Nicolae A (2016). Mutations in the JAK/STAT and RAS signaling pathways are common
in intestinal T-cell lymphomas. Leukemia.

[125] Afzal A, Esmaeili A, Ibrahimi S, Farooque U, Gehrs B (2020). Monomorphic Epitheliotropic Intestinal T-Cell Lymphoma With
Extraintestinal Areas of Peripheral T-Cell Lymphoma
Involvement. Cureus.

[126] Nakano Y (2021). Outcome of appendicular or scapular osteosarcoma treated by limb
amputation in cats: 67 cases (1997-2018). J Am Vet Med Assoc.

[127] Marconato L (2024). A retrospective Italian Society of Veterinary Oncology (SIONCOV)
study of 56 cats with appendicular osteosarcoma. Vet Comp Oncol.

[128] Marinoff AE (2023). Clinical Targeted Next-Generation Panel Sequencing Reveals MYC
Amplification Is a Poor Prognostic Factor in Osteosarcoma. JCO Precis Oncol.

[129] Xu H (2018). Genetic and clonal dissection of osteosarcoma progression and
lung metastasis. International Journal of Cancer.

[130] Sarver AL (2023). Distinct mechanisms of PTEN inactivation in dogs and humans
highlight convergent molecular events that drive cell division in the
pathogenesis of osteosarcoma. Cancer Genet.

[131] Worth LL, Lafleur EA, Jia SF, Kleinerman ES (2002). Fas expression inversely correlates with metastatic potential in
osteosarcoma cells. Oncol Rep.

[132] Wilson DW (2016). Tumors in Domestic Animals.

[133] Takahashi T (1989). p53: a frequent target for genetic abnormalities in lung
cancer. Science.

[134] Liao RG (2013). Inhibitor-sensitive FGFR2 and FGFR3 mutations in lung squamous
cell carcinoma. Cancer Res.

[135] Liao Y (2019). Targeted deep sequencing from multiple sources demonstrates
increased NOTCH1 alterations in lung cancer patient plasma. Cancer Med.

[136] Chen Y (2023). An antioxidant feedforward cycle coordinated by linker histone
variant H1.2 and NRF2 that drives nonsmall cell lung cancer
progression. Proc Natl Acad Sci U S A.

[137] Wallbillich NJ, Lu H (2023). Role of c-Myc in lung cancer: Progress, challenges, and
prospects. Chin Med J Pulm Crit Care Med.

[138] Subramanian J, Govindan R (2007). Lung cancer in never smokers: a review. Journal of clinical oncology.

[139] Goldschmidt MH, Goldschmidt KH (2016). Tumors in Domestic Animals.

[140] Murphy S (2013). Cutaneous squamous cell carcinoma in the cat: current
understanding and treatment approaches. J Feline Med Surg.

[141] Dotto GP, Rustgi AK (2016). Squamous Cell Cancers: A Unified Perspective on Biology and
Genetics. Cancer Cell.

[142] Chang D, Shain AH (2021). The landscape of driver mutations in cutaneous squamous cell
carcinoma. NPJ Genom Med.

[143] Jacobs MS, Persons DL, Fraga GR (2013). EGFR and MYC gene copy number aberrations are more common in
squamous cell carcinoma than keratoacanthoma: a FISH study. J Cutan Pathol.

[144] Trieu KG (2022). Basal cell carcinomas acquire secondary mutations to overcome
dormancy and progress from microscopic to macroscopic
disease. Cell Reports.

[145] Kilgour JM, Jia JL, Sarin KY (2021). Review of the Molecular Genetics of Basal Cell Carcinoma;
Inherited Susceptibility, Somatic Mutations, and Targeted
Therapeutics. Cancers (Basel).

[146] Kiupel M (2016). Tumors in Domestic Animals.

[147] Isotani M (2010). Mutations in the fifth immunoglobulin-like domain of kit are
common and potentially sensitive to imatinib mesylate in feline mast cell
tumours. Br J Haematol.

[148] Sabattini S (2013). Prognostic significance of Kit receptor tyrosine kinase
dysregulations in feline cutaneous mast cell tumors. Vet Pathol.

[149] Lanternier F (2008). Phenotypic and genotypic characteristics of mastocytosis
according to the age of onset. PLoS One.

[150] Hoffmann KM (2008). Successful treatment of progressive cutaneous mastocytosis with
imatinib in a 2-year-old boy carrying a somatic KIT mutation. Blood.

[151] Isotani M (2006). Identification of a c-kit exon 8 internal tandem duplication in a
feline mast cell tumor case and its favorable response to the tyrosine
kinase inhibitor imatinib mesylate. Vet Immunol Immunopathol.

[152] Matsumura T (2022). A Myb enhancer-guided analysis of basophil and mast cell
differentiation. Nat Commun.

[153] Munday JS, Kiupel M, Lohr CV (2016). Tumors in Domestic Animals.

[154] Renzi A (2019). Prevalence of p53 dysregulations in feline oral squamous cell
carcinoma and non-neoplastic oral mucosa. PLoS One.

[155] Wang YY (2021). Excision repair cross-complementing group 2 upregulation is a
potential predictive biomarker for oral squamous cell carcinoma
recurrence. Oncol Lett.

[156] Guberina M (2021). ERCC2 gene single-nucleotide polymorphism as a prognostic factor
for locally advanced head and neck carcinomas after definitive
cisplatin-based radiochemotherapy. Pharmacogenomics J.

[157] Vail DM, Thamm DH, Liptak JM, Vail DM, Thamm DH, Liptak JM (2020). Withrow and MacEwen’s Small Animal Clinical Oncology.

[158] Groll T (2021). Bridging the Species Gap: Morphological and Molecular Comparison
of Feline and Human Intestinal Carcinomas. Cancers (Basel).

[159] Cercek A (2021). A Comprehensive Comparison of Early-Onset and Average-Onset
Colorectal Cancers. J Natl Cancer Inst.

[160] Rowan AJ (2000). APC mutations in sporadic colorectal tumors: A mutational
“hotspot” and interdependence of the “two
hits”. Proc Natl Acad Sci U S A.

[161] Dörsam B (2018). PARP-1 protects against colorectal tumor induction, but promotes
inflammation-driven colorectal tumor progression. Proceedings of the National Academy of Sciences.

[162] Jin Z, Sinicrope FA (2022). Mismatch Repair-Deficient Colorectal Cancer: Building on
Checkpoint Blockade. J Clin Oncol.

[163] Cullen JM (2016). Tumors in Domestic Animals.

[164] Peng J (2023). Genetic alterations of KRAS and TP53 in intrahepatic
cholangiocarcinoma associated with poor prognosis. Open Life Sci.

[165] Luo G (2017). c-Myc promotes cholangiocarcinoma cells to overcome contact
inhibition via the mTOR pathway. Oncol Rep.

[166] Sasaki M, Nitta T, Sato Y, Nakanuma Y (2016). Loss of ARID1A Expression Presents a Novel Pathway of
Carcinogenesis in Biliary Carcinomas. Am J Clin Pathol.

[167] Linderman MJ, Brodsky EM, de Lorimier LP, Clifford CA, Post GS (2013). Feline exocrine pancreatic carcinoma: a retrospective study of 34
cases. Vet Comp Oncol.

[168] Zhang F (2021). TP53 mutational status-based genomic signature for prognosis and
predicting therapeutic response in pancreatic cancer. Frontiers in Cell and Developmental Biology.

[169] Oketch DJA, Giulietti M, Piva F (2023). Copy Number Variations in Pancreatic Cancer: From Biological
Significance to Clinical Utility. Int J Mol Sci.

[170] Zhang Z, Zhang H, Liao X, Tsai HI (2023). KRAS mutation: The booster of pancreatic ductal adenocarcinoma
transformation and progression. Front Cell Dev Biol.

[171] Luo J (2021). KRAS mutation in pancreatic cancer. Semin Oncol.

[172] Cony FG (2023). Pathological and immunohistochemical characterization of
pancreatic carcinoma in cats. Journal of Comparative Pathology.

[173] Abraham SC (2002). Genetic and immunohistochemical analysis of pancreatic acinar
cell carcinoma: frequent allelic loss on chromosome 11p and alterations in
the APC/beta-catenin pathway. Am J Pathol.

[174] Tichenor M, Hearon K, Selmic LE (2024). Characteristics and outcomes for 61 cats that underwent either
surgery or stereotactic radiotherapy as treatment for intracranial
meningioma (2005–2017). Journal of the American Veterinary Medical Association.

[175] Taher MM (2024). Next-Generation DNA Sequencing of Grade 1 Meningioma Tumours: A
Case Report of Angiomatous and Psammomatous Meningiomas. Cureus.

[176] Nassiri F (2021). A clinically applicable integrative molecular classification of
meningiomas. Nature.

[177] Rissi DR, Miller AD (2017). Feline glioma: a retrospective study and review of the
literature. J Feline Med Surg.

[178] Martinelli S (2006). Activating PTPN11 mutations play a minor role in pediatric and
adult solid tumors. Cancer Genet Cytogenet.

[179] Mayr L (2025). Effective targeting of PDGFRA-altered high-grade glioma with
avapritinib. Cancer Cell.

[180] Philpott C, Tovell H, Frayling IM, Cooper DN, Upadhyaya M (2017). The NF1 somatic mutational landscape in sporadic human
cancers. Hum Genomics.

