# Supplementary material for: The oncogenome of the domestic cat
Source: Science. Author manuscript; Available in PMC 2026 Mar 18. (PMC7618901; doi:10.1126/science.ady6651)
Supplement: Supplementary Text, Materials & Methods, Figures S1-S17 and legends for Supplementary Tables S1-S18 [file EMS212566-supplement-Supplementary_Text__Materials___Methods__Figures_S1_S17_and_legends_for_Supplementary_Tables_S1_S18.pdf]

Supplementary Materials for

The oncogenome of the domestic cat

Bailey A. Francis, Latasha Ludwig, Chang He, Melanie Dobromylskyj, Christof A. Bertram, Heike Aupperle-Lellbach, Hannah Wong, Aiden P. Foster, Mark J. Arends, Alejandro Suárez-Bonnet, Simon L. Priestnall, Laetitia Tatiersky, Fernanda Castillo-Alcala, Angie Rupp, Arlene Khachadoorian, Eda Parlak, Marine Inglebert, Shevaniee Umamaheswaran, Saamin Cheema, Martin Del Castillo Velasco-Herrera, Kim Wong, Ian C. Vermes, Jamie Billington, Sven Rottenberg, Geoffrey A. Wood, David J. Adams, Louise van der Weyden

Corresponding author: Louise van der Weyden, lvdw@sanger.ac.uk

The PDF file includes:

Materials and Methods
Supplementary Text
Supplementary Figures S1 to S19
Legends for Supplementary Tables S1 to S18

Other Supplementary Materials for this manuscript include the following:

Supplementary Tables S1-S18 can be accessed from Figshare (*18*)

Materials and Methods

Sample collection and DNA isolation

The tissue samples were formalin-fixed, paraffin-embedded (FFPE) feline tissues that had been collected as part of routine diagnostic procedures (biopsy or necropsy), with the owner's consent. The use of the samples adhered to Nagoya Protocol guidelines. The cases were ascertained from ten institutions, a mixture of commercial veterinary diagnostic companies and academic veterinary pathology departments, across six countries (Canada, England, Scotland, Germany, Austria and New Zealand). We used this design to reduce ascertainment bias and capture potential geographic variations. Original and/or re-cut hematoxylin and eosin sections (whole-slide images or glass slides) of each case were examined by board-certified, experienced veterinary anatomic pathologists, to confirm the diagnosis and annotate the areas to be sampled. The cases were selected based on the tumor size and the availability of FFPE normal (healthy) tissue from the same animal, either peri-lesional tissue or a different tissue altogether. Normal tissues were required to be histologically free from neoplastic and/or inflammatory lesions. Samples ('cores') were taken from the FFPE blocks using a 0.6 or 1.0 mm diameter punch/biopsy needle. Genomic DNA was extracted from the FFPE cores using the QIAamp DNA FFPE Tissue Kit (Qiagen, Manchester, UK), according to the manufacturer's instructions.

In addition to the FFPE cohort, a subset of fresh surplus mammary tissue was also obtained from domestic cats undergoing mastectomy for suspected mammary carcinoma in collaboration with practicing veterinarians and animal clinics in Switzerland between 2023 and 2024. In accordance with relevant Swiss guidelines and regulations, informed consent was obtained from the authorized welfare advocate of each participating cat, allowing for standard-of-care veterinary diagnostics and treatment and use of surplus tissues and/or EDTA-blood for research purposes. All animals in this study were handled according to the Swiss ethical standards and the ARRIVE guidelines where applicable, and sample collection was approved by the "Cantonal Committee for Animal Experiments" (Canton of Bern; permit BE82/2024). The mammary carcinoma samples were trimmed according to recommended guidelines for veterinary surgical pathology (*52*) and processed following the same histopathologic workflow described above. Unfixed leftover tissue was minced and either directly processed for primary culture generation or cryopreserved in freezing medium (45% Dulbecco's Modified Eagle Medium (Gibco, Thermo Fisher Scientific, Waltham, USA), 45% Fetal Calf Serum (ThermoFisher, Massachusetts, USA) and 10% dimethyl sulfoxide (DMSO; Signal-Aldrich, St. Louis, USA)) or frozen without additives for DNA isolation using the DNeasy kit (Qiagen, Hilden, Germany) following manufacturer's instructions.

Immunohistochemistry

For the lymphoma group, T- or B-cell phenotype was determined using immunohistochemistry (IHC) performed either on full face sections or on a tissue microarray created from the case materials. For the microarray cases, at least one core had to be composed of mostly neoplastic cells, and this population was positive for either CD3 (T-cell marker) or CD20 (B-cell marker). For the one case that was negative for both CD3 and CD20, Granzyme B and CD56 (NK-cell markers) were then used. The IHC was performed at two institutions, depending on where the FFPE blocks originated (see Table S2 for details). IHC on blocks from Austria was performed as previously described (*53*), and the primary antibodies included rabbit polyclonal CD3 at 1:1000 dilution (Agilent Cat# A0452, RRID:AB_2335677), rabbit polyclonal CD20 at

1:1000 dilution (Abcam Cat# ab27093, RRID:AB_470894), rabbit polyclonal Granzyme B at 1:400 (Abcam Cat# ab4059, RRID:AB_304251), and mouse monoclonal CD56 at 1:150 (LS Bio Cat#LS-B12970, clone IG4). IHC on blocks from Canada were performed as follows: antigen retrieval was carried out 110°C for 15 min in a citrate buffer (pH 6) solution and the primary antibodies included rabbit polyclonal CD3 at 1:100 dilution (Agilent Cat# A0452, RRID:AB_2335677), and rabbit polyclonal CD20 at 1:200 dilution (Lab Vision Cat# RB-9013-P1, RRID:AB_149767). Immunolabelling was performed on automated platforms, either Lab Vision AS 360 (Lab Vision, Thermo-Fisher Scientific, Fremont, California, USA) or Dako Autostainer (Dako/Agilent Technologies Canada Inc, Ontario, Canada), using standard protocols validated by an AAVLD-accredited laboratory (Animal Health Laboratory, Guelph, Ontario, Canada). Normal tissue from feline spleen, lymph node and/or small intestine were used as a positive control, whereas the omission of the primary antibody was used as a negative control.

For the mammary carcinoma cohort from Canada, estrogen receptor (ER) status was determined using IHC for ER which was performed manually on tissue microarrays and evaluated by a board-certified veterinary anatomic pathologist. Briefly, slides were baked for 4-5 hr at 37°C and then deparaffinized and dehydrated in xylene and graded alcohols. Endogenous peroxidases were quenched with 3% hydrogen peroxide for 5 min at room temperature (RT). Antigen retrieval was performed at 110°C for 15 min in a citrate buffer solution (Invitrogen eBioscience IHC Antigen Retrieval Solution – low pH). Slides were blocked with normal horse serum (VECTASTAIN ABC-HRP Kit, Peroxidase, mouse Ig; BioLynx, Ontario, Canada) for 20 min, followed by avidin/biotin for 15 min (Vector Avidin/Biotin Blocking Kit) at RT. Slides were incubated with a mouse monoclonal ERα antibody at 1:400 dilution (Thermo Fisher Scientific Cat# MA5-13191, RRID:AB_10986080) overnight at 4°C. The secondary antibody (biotinylated horse anti-mouse IgG diluted as per the manufacturer's protocol; VECTASTAIN ABC-HRP Kit) was incubated at RT for 30 min. ABC reagent from the same kit was incubated for 30 min at RT. Slides were finally incubated with DAB (Vector DAB Substrate Kit, Peroxidase (HRP), with Nickel) for 5 min at RT and counterstained with Modified Harris Hematoxylin. For the mammary carcinoma cohort from Switzerland, IHC for ER was performed using a rabbit monoclonal ERα antibody at 1:50 dilution or 3 µg/mL (Agilent Cat# IR084, RRID:AB_2617140) on the automated Bond-III Immunostainer (Leica Biosystems, Buffalo Grove, USA) where slides were pretreated with Epitope Retrieval Buffer Type 2 (Tris-EDTA, pH 9; Leica Biosystems, Buffalo Grove, USA) for 40 min at 100 °C. For both cohorts, ER status was classified as: ER negative (<1% of cells with nuclear expression, ER low positive (1-9% of cells with nuclear expression), or ER positive (≥10% of cells with nuclear expression), according to human guidelines (*54*).

For the mammary carcinoma cohort from Switzerland, 2-3 µm FFPE sections were mounted on positively charged slides (Color Frosted Plus, Leica Biosystems, Muttenz, Switzerland) and processed using the automated Bond-III immunostainer (Leica Biosystems, Melbourne, Australia). Slides were pretreated with Epitope Retrieval Buffer Type 2 (Tris-EDTA, pH 9; Leica Biosystems) for 40 min at 100 °C for the rabbit monoclonal ERα antibody (1:50 dilution; Agilent Cat# IR084, RRID:AB_2617140) and for 30 min at 100 °C for the rabbit FBXW7 antibody (1:500 dilution; Thermo Fisher Scientific Cat# MA5-50723, RRID:AB_3093009). A protein block was applied for 10 min at room temperature to reduce non-specific binding, followed by primary antibody incubation for 15 min. Detection was performed using the Bond Polymer Refine Detection Kit (Leica Biosystems Cat# DS9800, RRID:AB_2891238), including peroxidase blocking (5 min), anti-rabbit secondary antibody (8 min), and polymer reagent (8 min); the latter two steps were supplemented with 2% dog serum (LabForce, Nunningen, Switzerland) to

minimize non-specific binding. Slides were developed with DAB/H_2O_2 (10 min), counterstained with hematoxylin, and mounted. Given the lack of validated reference criteria for FBXW7 IHC, interpretation remained descriptive focusing on reduced vs not reduced nuclear signal. Assessment was guided by sequencing data and contrasts in nuclear staining across different MAM samples with adjacent normal tissue (e.g. stromal cells with unaltered FBXW7 signal) serving as internal reference.

Sequencing, read alignment and quality control

To capture the feline oncogenomic landscape, we compiled a list of 1,039 human cancer-associated genes (including MSK-IMPACT™ and MSK-IMPACT Heme™ panels (*55*), COSMIC Cancer Gene Census Tier 1 genes (*56*), and cancer genome landscape genes (*57*) and obtained the feline orthologs; from this, a total of 986 feline genes were included in our bait library (detailed in Table S14). Specifically, the baits (6.3 Mb) were designed against the feline reference genome Felis_Catus_9.0 (FelCat9) (*49*) for exon target capture of 986 feline genes and up to 125 bp of their flanking sequences, as well as 500 bp upstream of the *TERT* transcription start codon, as previously detailed (*15*). Our SureSelect CD Feline Oncogenome bait set (Agilent Technologies, Cheshire, UK) achieved 99.1% coverage of the targeted exons. To capture the whole-exome of the feline mammary carcinoma cohort from Switzerland, our SureSelect CD Feline Exome bait set (Agilent Technologies), as previously detailed (*16*), was used.

Preparation of DNA sequencing libraries, capture using SureSelect Feline baits and paired-end sequencing using the NovaSeq6000 platform (Illumina) to generate 101 bp reads, were all performed as previously described (*15*). Sequencing reads from the samples were aligned to the FelCat9 reference genome using BWA-MEM (*58*) and PCR duplicates were marked using Biobambam2 bammarkduplicates2 (v2.0.146) (*59*). Tumor-normal sample concordance was assessed using bcftools gtcheck (v1.9) (*60*). For the ‘oncogenome’ bait cohort, samples were excluded if failing quality control (QC), which included <80% of the targeted regions covered with ≤ 71X coverage, or cross-individual contamination >5% in either the tumor or matched normal; the median sequence coverage of targeted regions was 234-fold for the samples that passed QC, when PCR duplicates were excluded. For the ‘exome’ bait cohort, samples were excluded if failing quality control (QC), which included <80% of the targeted regions covered with ≤ 21X coverage, or cross-individual contamination >5% in either the tumor or matched normal; the median sequence coverage of targeted regions was 78-fold for the samples that passed QC, when PCR duplicates were excluded.

Identification and annotation of somatic variants

Somatic mutations were identified using cgpCaVEMan (v1.15.2) (*61*) for single nucleotide variants (SNVs), and cgpPindel (v.3.10.0) (*62*) for insertions/deletions (indels). Details of the methods for each algorithm and the specific parameters, input files and flagging rules that were used are as follows (using FUR Caveman and Pindel Reference files (*63*)):

<u>cgpCaveman</u>

```
caveman.pl \
  -reference "Felis_catus.Felis_catus_9.0.dna.toplevel.fa.fai" \
  -outdir "/path/to/results" \
  -tumour-bam "/path/to/tumour.bam" \
  -normal-bam "/path/to/normal.bam" \
  -ignore-file "ignore_file.bed" \
  -tum-cn-default 5 \
```

```
  -norm-cn-default 2 \
  -species "Felis_catus" \
  -species-assembly "Felis_catus_9.0" \
  -unmatched-vcf "/path/to/unmatched_directory/" \
  -seqType "targeted" \
  -tumour-protocol "TARGETED" \
  -normal-protocol "TARGETED" \
  -normal-contamination 0.1 \
  -noflag
```

<u>cgpFlagCaVEMan</u>

```
cgpFlagCaVEMan.pl \
  -i /path/to/caveman.vcf.gz \
  -o /path/to/caveman.flagged.vcf.gz \
  -s CAT_WXS \
  -sa Felis_catus_9.0 \
  -m /path/to/tumour.bam \
  -n /path/to/normal.bam \
  -b /path/to/bedfiles_directory \
  -umv /path/to/unmatched_vcf_directory \
  -ab /path/to/annotation_directory \
  -ref /path/to/Felis_catus.Felis_catus_9.0.dna.toplevel.fa \
  -c /path/to/flag.vcf.config.ini \
  -v /path/to/flag.to.vcf.convert.ini \
  -t targeted
```

<u>cgpPindel</u>

```
pindel.pl \
  -reference "/path/to/Felis_catus.Felis_catus_9.0.dna.toplevel.fa.fai" \
  -outdir "/path/to/results" \
  -tumour "/path/to/tumour.bam" \
  -normal "/path/to/normal.bam" \
  -simrep "/path/to/simpleRepeats.bed.gz" \
  -seqtype "TGS" \
  -exclude "KZ%,AANG%" \
  -filter "/path/to/TARGETED_Rules.lst" \
  -genes "/path/to/codingexon_regions.indel.bed.gz" \
  -unmatched "/path/to/pindel_np.empty.gff3.gz" \
  -cpus 1 \
  -badloci "/path/to/HiDepth_v1.bed.gz" \
  -softfil "/path/to/softRulesFragment.lst"
```

<u>Ensembl VEP</u>

```
vep -i /path/to/input.vcf.gz \
  --output_file /path/to/input.vep.vcf.gz \
  --cache \
  --dir /path/to/cache/ \
  --fasta /path/to/Felis_catus.Felis_catus_9.0.dna.toplevel.fa.gz \
  --db_version 104 \
  --species felis_catus \
  --assembly Felis_catus_9.0 \
  --offline \
  --custom
/path/to/SRA_99_Lives.snp_indel.filt.54cats.short.alt.no_header.vcf.gz,99_Liv
es,vcf,exact,0,AF \
```

```
-t SO \
--format vcf \
--buffer_size 20000 \
--symbol \
--biotype \
--vcf \
--sift s \
--no_stats \
--flag_pick_allele_gene \
--canonical \
--hgvs \
--shift_hgvs 1 \
--compress_output bgzip \
--mane \
--protein \
--numbers \
--fork 4 \
--domains
```

Multi-nucleotide variants (MNVs) were identified from cgpCaVEMan SNVs using an in-house Nextflow pipeline, Justaphase (*64*), which uses WhatsHap (v2.3) (*65*) to phase variants, CASM-Smart-Phase (*66*), bcftools (v1.19) (*60*) and bedtools (2.31.1) (*67*) to find adjacent SNVs, and an in-house python package, FUR-phaser (*68*), to reconstruct MNVs from adjacent SNVs in the same phase group. The Ensembl (v104) Variant Effect Predictor (VEP) (*69*) was used to predict the consequences of base changes and indels on proteins. As a gene may have multiple transcripts, the canonical transcript, as defined in Ensembl, was used to determine the variant consequence.

After variant annotation we used a custom workflow, QC (*70*), to quality control the variant calls produced by cgpCaVEMan, FUR-phaser, and cgpPindel. Variants overlapping common SNPs in the 99 Lives Project VCF (*48*) were excluded as putative germline events. For this study, we obtained the updated 418-cat 99 Lives VCF, which was based on the Fca126 assembly (GCA_018350175.1), and lifted over the variant coordinates to the Felis_catus_9.0 assembly using Picard LiftoverVcf (v3.4.0) (*71*) prior to filtering. cgpPindel calls were then filtered to retain only those with a variant allele fraction (VAF) ≥ 0.10. We further selected MNVs of ≤ 3 bp with equal REF and ALT lengths and indels in which both the reference and alternate alleles were ≤ 25 bp in length (excluding any where both alleles exceeded 10 bp). Larger indels (> 25 bp) were kept only if they had a coverage depth > 20×, a VAF > 0.25 in both tumor and matched normal samples and were not classified as complex. Finally, all variants within 100 bp of the targeted regions that passed both the cgpCaVEMan and cgpPindel filter flags were advanced for further analysis.

After filtering, variants calls were converted into two Mutation Annotation Format (MAF) files – one containing the filtered variants (keep) and another containing only the protein-altering (keepPA) variants – using a custom script, MAF (*72*). We processed each MAF file with a custom workflow designed for the liftover of somatic variants from one assembly to another. Briefly, for every input MAF, we used maf2vcf (v1.6.22) (*73*) to generate per-matched-tumor-normal-pair VCFs against the source assembly (Felis_catus_9.0). Variants in each VCF were then lifted over to the target assembly (Fca126) using Picard LiftoverVcf with the UCSC Felis_catus_9.0 -> Fca126 chain file. Finally, sample-level VCFs were converted into cohort-level MAFs relative to the target assembly using a custom script, MAF (*72*). Oncoplots were created from the keepPA variants in a cohort using Maftools (*74*). Tumor mutational burden (TMB) was calculated by counting the number of mutations in each cohort's keepPA MAF file and plotted using TMB_plotter (*75*).

Given the known artefact profile in FFPE data and the possibility of erroneous and missed calls from cgpCaVEMan and cgpPindel, we inspected hotspot locations, defined as recurrent somatic sequence alterations occurring at the same genomic position in ≥ 3 independent samples, in greater detail. Using another purpose-built Python package MAF_Updater (*76*), we refined the initial variant call set at mutational hotspots by extracting variants that met the hotspot criteria from MAF files and generating a pileup at the locus across samples using bcftools (v1.19) (*60*). We computed mean alternate allele counts, total coverage, and variant allele frequencies (VAF)) from these pileups to assess the veracity of hotspot variant calls, categorising them into the following types: 1) true positive: variant supported by the pileup and was present in the MAF file; 2) false positive: variant is present in the MAF file with no support from the mpileup; 3) true negative: variant received no support from the pileup and was not present in the MAF file; 4) false negative: variant supported by the pileup but was not present in the MAF file. With these metrics, SNP and indels were filtered according to type–specific thresholds. Variants initially called by cgpCaVEMan/Pindel that exhibited a tumor VAF below 2% or more than three alternate allele reads in the matched normal sample were re-classified as false positive variants. In contrast, variants present in a pileup but originally missed by cgpCaVEMan/cgpPindel with a minimum of five tumor alternate reads and no more than three alternate reads in the normal sample were reclassified as true somatic events. If a true positive variant in a sample was flagged as false negative in more than three other tumor–normal pairs, it was deemed likely germline and removed from the call set. This approach yielded a curated MAF file enriched for high-confidence somatic hotspot mutations that was used in downstream analyses. To further support the variants called, JBrowse (*77*) was used to perform manual inspections of all protein-altering mutations in driver genes (with a significant q-global value >0.001) and all hotspot mutations. From this search, 8 variants were subsequently removed from the final call set. A summary of the reasons for variant removal and list of the variants removed is in Table S15.

An identical analytical workflow was applied to the feline mammary whole exome sequencing (WES) cohort. For this dataset cgpCaVEMan and cgpPindel were run in WES mode rather than targeted mode. cgpCaVEMan was run with the following arguments -tumour-protocol WES -normal-protocol WES -seqType exome. cgpPindel was run with the following arguments -seqtype WES. All subsequent steps were performed as described above including variant phasing and MNV reconstruction, VEP annotation using Ensembl canonical transcripts, liftover and SNP masking against 99 Lives, QC and flag filtering, and MAF generation and summary analyses.

Identification of somatic copy number alterations

Somatic copy number alterations (CNAs) were identified using a python workflow, fur_cnvkit (*78*), built on top of CNVkit (v0.9.10) (*79*). In this workflow, CNVKit leverages both on-and off-target reads from a targeted sequencing experiment to infer copy number across the entire genome; additional steps enable the quality control of copy number profiles and the removal of poor-quality samples from the dataset.

Within the workflow, the CNVkit access subcommand was first used to identify sequencing-accessible regions of the FelCat9 genome assembly. Sequencing-accessible regions, the genome were split into target regions (captured by our targeted sequencing panel) and anti-target regions (not captured). To ensure sufficient coverage in each region for reliable copy number estimation, regions were subdivided into bins using the CNVKit autobin subcommand. Median bin sizes determined for target and anti-target regions were 614 base pairs and 58,834 base pairs, respectively.

After bin determination, a male-specific and a female-specific pooled copy number reference was constructed for the entire dataset. For constructing these references, we used normal vs. normal copy number calling to identify poor quality normal samples, following protocols outlined previously (*80*). Sample quality was assessed by calculating, for each normal sample, the average of the median log2 fold-change values from gene-level copy-number ratios across all pairwise normal–normal comparisons within each study; larger deviations in these medians reflect more aberrant copy-number profiles that could yield spurious calls, and accordingly samples in the upper or lower 10th percentile were deemed poor quality and excluded from downstream reference construction. To categorize samples with an unknown sex, we used the sex subcommand in CNVkit to predict each sample's sex based on the relative read coverages observed on chromosomes X and Y. The filtered list of normal samples used in the copy-number references are listed in Table S16A.

Following construction of these sex-specific copy number references for the study, copy number alterations in the feline tumor samples were called using the CNVkit batch subcommand with the circular binary segmentation algorithm (-m cbs). To account for log2 differences in copy number neutral regions observed between samples, we used CNVKit's re-centering function (--center median) to adjust all segment-level log2 ratios using median centering.

Copy number gains (amplifications) in the centered data were defined as net increases with a log2 ratio > 0.32 (corresponding to an approximate 1.25-fold increase in DNA content), whereas copy number losses (deletions) were defined as net decreases with a log2 ratio < –0.4 (corresponding to an approximate 0.75-fold decrease in DNA content). With these thresholds, we utilized the CNVKit genemetrics subcommand to call gene-level gains/losses in each tumor sample, using the sample's log2 ratio and centred segmentation data as input.

To reduce false positive copy number calls caused by samples with over-segmented and highly variable copy number calls, a metric was devised for summarising copy number profile quality, which was used to identify and exclude outlier samples (listed in Table S16B):

To reduce false positive copy number calls caused by samples with over-segmented and highly variable profiles, we first denote the log2 copy-number ratio measurements for sample i as

$$\{x_{i,j}\}_{j=1}^{n_i}, \quad x_{i,j} = \log_2(\text{copy-number ratio}_{i,j})$$

and compute its median absolute deviation

$$\text{MAD}_i = \text{median}_j\left(\left|x_{i,j} - \text{median}_k(x_{i,k})\right|\right).$$

We then calculate the mean segment length

$$\bar{s}_i = \frac{1}{m_i}\sum_{l=1}^{m_i}(\text{end}_{i,l} - \text{start}_{i,l}),$$

and form an adjusted dispersion metric

$$M_i = \frac{\text{MAD}_i}{\log_2(\bar{s}_i + 2)},$$

such that larger segments reduce the effective dispersion metric. To identify outliers across all N samples, we compute

$$\widetilde{M} = \text{median}_i(M_i), \quad \text{MAD}_M = \text{median}_i\left(\left|M_i - \widetilde{M}\right|\right),$$

and the modified z-score for each sample

$$z_i = \frac{0.6745\,(M_i - \widetilde{M})}{\text{MAD}_M}.$$

Mutational signature analysis

Signature fitting was performed on tumor samples with ≥100 somatic mutations using SigFit (v2.2) (*81*). Mutational opportunities were calculated from the feline bait set and used to convert mutation catalogues relative to the human genome. The 'sig_fitting' function was used to

fit COSMIC signatures (v3.4) and identify significant exposures (contributing ≥10% of mutations). Re-fitting was performed, restricting reference signatures to significant exposures for more accurate estimates, except when only one significant exposure was detected. Reliability of the fitting results was assessed using a cosine similarity threshold of ≥ 0.850 between the original and reconstructed mutational spectra.

Identification of significantly mutated genes

To identify driver genes in each cohort we used dNdScv (v0.1.0, git commit 0633182) (*82*), which detects genes under positive selection in cancer. To run dNdScv on feline samples, we generated a dNdScv reference database using the FelCat9 genome and the FelCat9 Ensembl v104 canonical transcripts, according to the instructions (*83*). To prepare variants for analysis, the MAF file for each cohort (containing variants that passed upstream QC) was further filtered to remove any indels with VAF <0.1 and any variants present in the 99 Lives dataset (*49*). For this step, we used the updated 418-cat 99 Lives VCF, which was based on the Fca126 assembly, and lifted over the variant coordinates to the Felis_catus_9.0 assembly using Picard LiftoverVcf (v3.4.0). Finally, the filtered MAF files were reformatted to input into dNdScv. dNdScv was run using the 'without covariates' option with a max mutations per-gene-per-sample set at 2. The dNdScv outputs included q-values that were adjusted using the Benjamini-Hochberg procedure. A gene was identified as a 'driver' if the q-global value was <0.1 and the gene was mutated in more than one sample. All code, inputs and outputs required for reproducing this analysis are available (*84*).

Identification and annotation of germline variants

Germline variant calls were generated using an in-house Nextflow pipeline, dermatlas_germlinepost_nf (*85*), that performs variant calling and several pre- and post-processing steps. First, normal sample BAM files from each cohort were coordinate-sorted with sambamba (v0.6.5), marked for PCR duplicates using the Genome Analysis Toolkit (GATK v4.2.6.1), and re-sorted with sambamba (v0.6.5). The re-sorted BAM files were converted to CRAM format. Variants in each CRAM file were identified using several GATK tools: they were called using HaplotypeCaller, consolidated into GenomicsDB format, and then joint genotyped with GenotypeGVCFs.

Raw variant calls were separated by type (SNPs and INDELs) using GATK's SelectVariants tool and marked using GATK's VariantFiltration with variant type-specific filtering criteria. SNPs were marked using the following criteria: QD < 2.0, QUAL < 30.0, SOR > 3.0, FS > 60.0, MQ < 40.0, MQRankSum < -12.5, and ReadPosRankSum < -8.0. INDELs were marked using the following criteria: QD < 2.0, QUAL < 30.0, FS > 200.0, and ReadPosRankSum < -20.0. After marking, variants flagged as "PASS" and situated within target regions defined by the capture bait set were retained by filtering with bcftools (v1.9).

Variant consequences were predicted using Ensembl VEP (v104) and the resulting VCF files were converted to pseudo-MAF format files for post-processing and plotting. We selected variants with VAF ≥ 0.25 and > 6 total read depth, and curated a list of candidate cancer predisposition variants by identifying those predicted to result in a loss of function (LOF; frameshift indel, nonsense variant, splice site variant, transcriptional start and stop codon loss) and affecting a gene with a human ortholog that is a gene used in England's National Health Service (NHS) Cancer National Genomic Test panel (v7.2 June-2023). The candidates were further filtered by removing variants identified as SNPs in the updated 418-cat 99 Lives dataset (originally based on the Fca126 assembly and lifted over to Felis_catus_9.0 with Picard LiftoverVcf). Finally, visual

inspection of sequencing reads (using JBrowse) was performed to remove low-quality variant calls. A recurrent *MAP2K2* indel initially identified as a frameshift variant in exon 11 of the MAP2K2 transcript ENSFCAT00000014141.5 was removed, as the evidence for the presence of this exon is based on RefSeq computational prediction alone and not experimental evidence or homology with other species.

To test whether the feline germline variants were orthologous to any human cancer predisposition variants, the human ClinVar variants were lifted over from GRCh38 to Felis_catus_9.0 with Picard LiftoverVcf using the UCSC hg38ToFelCat9 chain file.

A two-sided Wilcoxon rank-sum test was used to compare the ages of cats with and without candidate cancer predisposition variants. Prior to performing this test, a Shapiro-Wilk test was used, and found that the data were not normally distributed, making the choice of a non-parametric statistical test suitable for this analysis.

Viral analysis

Pathogen identification in the tumor and normal samples was performed using another in-house workflow, pathogen_identification (*86*). Briefly, unaligned read pairs from the QC-passed BAM files were extracted and converted into fastq files using samtools (v1.19). These fastq files were provided as inputs to Kraken2, which was run against the PlusPFP reference database (version of March 2023) (*87*). To mitigate the risk of false-positive taxa discoveries that can occur when using Kraken2, we ran the tool with the confidence score threshold set at 0.1. From the Kraken2 results, we calculated the proportion of minimizers from the reference database found in each sample for each species as: *Proportion = Distinct minimizers / Total clade–level minimizers*. These proportions served as our primary metric for assessing the presence of specific taxa within each sample.

To assess the significance of a species discovery in the Kraken data we attempted to estimate the "background" rate at which minimizers might be discovered by chance using a normal approximation to the binomial distribution. This approach models the null hypothesis that all minimizers in the reference database have an equal probability of being found by random chance. The probability of finding a specific number of minimizers (m) for a species can be considered using a binomial distribution: $X \sim Bin(n, p)$, where X represents the number of minimizers detected and:

- n = Total number of reads analyzed in the sample
- C = Total number of minimizers representing a clade in the reference database
- T = Total number of minimizers in the reference database
- p = Probability of obtaining a minimizer matching the given clade = C/T

Given the large number of reads in each sample, we approximated this binomial distribution with a normal distribution defined by:

- Mean (μ) = $n \times p$
- Standard deviation (σ) = $\sqrt{(n \times p \times (1-p))}$

For each taxon in each sample, we calculated the probability (*p-value)* of observing at least the number of detected distinct minimizers under this random sampling model using the right-tailed probability of the normal distribution:

$$P(X \geq m) = 1 - \Phi((m - \mu)/\sigma)$$

where Φ represents the cumulative distribution function of the standard normal distribution. Next, we applied the Benjamini-Hochberg procedure to the resulting p-values to correct for multiple comparisons and species with an adjusted p-value <0.05 were considered statistically significant. After statistical testing, the dataset was filtered for plotting by removing any viral taxa with fewer than five distinct minimizers observed per-sample across the cohort. Note: infections by feline retroviruses associated with cancer (such as FeLV and FIV) could not be accurately detected using DNA sequencing alone because reads that might be derived from these infectious agents also map to the FelCat9 reference genome build. Any Kraken minimizers that would be assigned to these retrovirus species would therefore be ambiguous; they might represent a genuine retroviral infection or reflect the presence of endogenous retroviral remnant sequences.

Humanization of feline single nucleotide variation data

We used a custom Python script to humanize feline single nucleotide variation data (see 'human-comparison-plot/src/fur2cosmic/main.py' in (*88*)). Briefly, cDNA alignments for human and feline orthologs were retrieved via the Ensembl REST API (v113). Ungapped alignments were used to create a nucleotide position mapping from feline to human coordinates. Feline SNV mutations, extracted from MAF files as simple HGVSc substitutions, were mapped onto the human cDNA, translated into protein sequences using Biopython, and annotated with HGVS nomenclature. These "humanized" feline mutations were then compared to human COSMIC data (v101) (*89*) to assess mutation concordance. Functional annotations were assigned to these variants using the MutationMapper tool (*90*) in cBioPortal (*91*).

Cross-species comparative analysis of single nucleotide variation

The SNV mutational frequencies for each of the human pan-cancer datasets used in this study were accessed from cBioportal. The datasets were 'TCGA' (*34, 92*), 'MSK-IMPACT' (*55, 93*) and 'China Pan-cancer' (*94, 95*).

Actionability analysis

To assess 'chemical tractability', the Pharos (v3.19.10) web interface (*96*) was used to extract data from the Target Central Resource Database (TCRD, v6.13.5), which curates and aggregates information on the current ability to perturb proteins with small molecules, biologics, and/or other therapeutic modalities; information related to human orthologs of the feline driver genes were extracted (*97*).

To assess 'synthetic lethal tractability', the Schaffer (*31*) database of preclinically validated synthetic lethal pairs was downloaded and the human orthologs of the feline driver genes were extracted.

To assess 'clinical tractability' we leveraged the OncoKB (*32, 98*) actionable genes database (*99*) (last updated 28th March 2025). Briefly, the associated databases were downloaded and the human orthologs of the feline driver genes were extracted. If the drug was associated with a specific SNV, this mutation was searched for in our set of 'humanized' feline SNVs.

Hotspot validation using Sanger sequencing

The region of interest for each hotspot variant for the mammary carcinoma and cutaneous mast cell tumor cohorts was amplified using ThermoFisher Platinum HiFi *Taq* DNA polymerase (following the manufacturer's instructions) using the primers shown in Table S17. Cases were selected if sufficient DNA remained and the variant allele frequency of the mutation in the tumor was > 0.1. Amplified products were sequenced by Sanger Sequencing (Eurofins) using the same oligos. Sequence traces were analyzed by visual inspection.

Figure generation

The code for generating the figures based on analysis of the sequencing data is archived within Zenodo (*88*).

Tumoroid establishment and culture

Tumoroids were established using a protocol previously described for canine and mouse mammary tissue (*100, 101*) with small modifications. Briefly, fresh tissue or thawed DMSO-frozen fragments were digested in collagenase (2 mg/mL; Sigma-Aldrich, St. Louis, USA) at 37°C with agitation until a fragment-free suspension was obtained. The suspension was centrifuged, and the pellet was resuspended in Cultrex® PathClear Reduced Growth Factors Basement Membrane Extract Type 2 (BME; Amsbio, Abingdon, England; 50% v/v in complete tumoroid culture medium (Table S18).

Tumoroid embedded in BME were seeded as droplets of up to 40 µL in total per 24-well plate (Nunc non-treated multidish, Thermo Fisher Scientific, Waltham, USA) and incubated at 37°C to allow polymerization. The droplets were overlaid with 500 µL culture medium per well and refreshed every 3-4 days until tumoroids were ready to be passaged or used for downstream experiments including genomic DNA isolation (using a DNAeasy kit, Qiagen) or IHC.

For histological assessment, tumoroid cultures were collected once confluent and fixed in 4% paraformaldehyde (PFA) for 2 hrs, embedded in 4% agarose or Blockfast (Hospitex International, Florence, Italy) according to the manufacturer's instructions, and subsequently processed for standard paraffin embedding. Both formalin-fixed paraffin-embedded (FFPE) tissue samples and tumoroid pellets were sectioned at 3 µm thickness. Sections were stained with hematoxylin and eosin or used for IHC (FBXW7 staining).

Tumoroid drug testing and viability assay

Tumoroids were enzymatically dissociated into single cells, and a total of 5,000 single cells per well were seeded into 96-well plates in 30 µL of polymerized BME (50% v/v in complete tumoroid culture medium). Tumoroids were overlaid with complete growth medium supplemented with varying concentrations of vincristine or vinorelbine. Vincristine was dissolved in DMSO and diluted in culture medium, with vehicle controls receiving the corresponding final concentration of DMSO. Vinorelbine was reconstituted in water and diluted directly into culture medium. Tumoroids were exposed to drugs for 48 hrs, followed by 3-4 days of culture in drug-free medium until viability assessment.

Tumoroid viability was assessed using the resazurin-based CellTiter-Blue assay (Promega, Madison, USA), following the manufacturer's instructions. To avoid signal oversaturation under our culture conditions, 5% (v/v) of CellTiter-Blue reagent was added to the medium instead of the standard 20%. Experiments were performed using three independent tumoroid lines representing biological replicates. For each line, two independently cultured experiments were performed to

account for within-line variation, and each condition was assayed in technical triplicate. Results were normalized to untreated controls and growth inhibition data were fitted to a four-parameter logistic sigmoidal model to determine log(IC_{50}) values, with comparisons made using the extra sum-of-squares F-test, all performed in Prism (v10.3.1; GraphPad Software, San Diego, CA, USA). This test was chosen to assess whether the data are better fit by separate curves for each genotype or a single shared curve; with the significant result obtained suggesting differences in curve shapes between the genotypes and therefore distinct drug response profiles. IC_{50} values are reported as the back-transformed absolute concentrations.

Supplementary Text

Key oncogenomic findings in the mammary tumors

Mammary carcinomas (MAM) are common in cats; most being malignant at presentation and associated with aggressive behavior (recurrence and metastasis) and poor prognosis, despite treatment with surgery and chemotherapy (reviewed in (*102*)). There were seven driver genes identified in our MAM cohort, specifically *FBXW7, PIK3CA, TP53, PTEN, PIK3R1, GTF21* and *RPL22* (Fig. 1C). The most mutated driver gene was the TSG *FBXW7*; 25/47 (53%) tumors had *FBXW7* mutations, with 14/25 (56%) of these having two or more mutations (Fig. S4). Recurrent CN losses on chr B1, encompassing *FBXW7,* were seen in 14/47 (30%) tumors. It is worth noting that *FBXW7* mutant cases in human breast cancer are associated with a worse prognosis, which shows strong clinical parallels with feline mammary tumors; most cat mammary tumors are malignant at presentation and associated with aggressive behavior and poor prognosis (*102*).

The second most frequently mutated driver gene was the oncogene *PIK3CA*; 22/47 (47%) tumors had *PIK3CA* mutations (predominantly a missense mutation at hotspot p.H1047R), with 18/22 (82%) of the *PIK3CA*-mutated tumors having a concomitant *FBXW7* mutation). Recurrent CN gains on chr C2, encompassing *PIK3CA,* were seen in 3/47 (6%) tumors (Fig. S4). Recurrent CN losses on chr C1, encompassing the driver gene *RPL22*, were seen in 12/47 (26%) tumors. Alterations in the driver gene *TP53* have been previously investigated in feline MAM, with *TP53* mutations identified by Sanger sequencing in 1/7 cases (*103*) and 0/5 cases (*104*). By RT-PCR, an over-expression of *TP53* relative to disease-free mammary tissue was found in 33% of MAM cases (n = 24 cats) (*105*).

The most recurrent CN alteration (CNA) was loss of regions on chr D2 encompassing *PTEN* (22/47, 47% tumors) and *FAS* (19/47, 40% tumors; Table S9). Recurrent CN gains on chr F2, encompassing *MYC*, were seen in 9/47 (19%) tumors (Fig. S4 and Table S9). Some tumors showed concomitant mutation and CN loss of driver genes, suggesting bi-allelic inactivation of these tumor suppressor genes, including *FBXW7* (3/47), *PTEN* (2/47) and *RPL22* (1/47).

The mutational profiles of feline mammary carcinoma show strong similarities to that of both canine mammary carcinoma and human breast cancer. For example, canine mammary carcinomas are characterized by frequent CN gain of *MYC* and loss of *PTEN* (*106*). In human breast cancer, *TP53* is the most mutated gene (~30% cases) and generally correlates with worse survival (*107*), with *PIK3CA* mutations found in 7-35% of cases (*108*), and *PTEN* inactivation (mutations or CN loss) occurring in patients with advanced disease and poor prognosis (*109*).

We performed immunohistochemistry on the samples to assess ER status and determine if there was a correlation with the mutational profile. Most of the samples (51%) were ER negative (17% ER positive, 30% weakly ER positive, 2% undetermined; Table S1), and we did not find any differences in the mutational landscape between the different ER statuses (Fig. S4).

Key oncogenomic findings in the lymphomas

Lymphoma (LYM) is the most frequently occurring malignancy in cats. Feline LYM can be classified by anatomical location, with the most common form being alimentary (with variable extraintestinal involvement), and immunophenotype predominantly T-cell or B-cell (with NK-cell LYM being extremely rare) (*110*). The most common type of lymphoma in cats is alimentary/gastrointestinal lymphoma (since the vaccine against feline leukemia virus reduced the incidence of viral-induced non-alimentary lymphomas); the majority are T-cell in origin and

typically involve the small intestine, whereas those of B-cell origin are typically found in the stomach/ileo-cecal-colic junction (*111-114*). Non-alimentary lymphoma also accounts for a large proportion of lymphoma in cats, although reports vary regarding the relative prevalence of anatomic forms across studies. Common types include mediastinal lymphoma (typically T-cell in origin), multicentric (nodal) (typically B-cell in origin), and various extranodal forms involving internal organs such as kidney or liver, central or peripheral nervous system, skin, or eyes (which can all be either B- or T-cell in origin) (*113-116*). In addition, lymphoma is the most common neoplasia of the upper respiratory tract (nasal cavity, nasopharynx), and these are typically B-cell in origin (*113, 114, 117*). In contrast to cats, ~80% of canine lymphoma cases are multicentric and typically B-cell in origin (~60%), with diffuse large B-cell lymphoma being the most common subtype, while alimentary as well as cutaneous, mediastinal, and other site-specific lymphomas are far less common in comparison (*113, 114, 118*).

Our feline LYM cohort was immunophenotyped after sequencing was performed, and comprised of 29 B-cell, 21 T-cell and one NK-cell subtype.

In the B-cell cases (14 alimentary, 15 non-alimentary), ten driver genes were identified, specifically, *P2RY8, B2M, TRAF3, TP53, IKZF3, MAPK21, CASP8, GNA13, TNFAIP3*, and *FAS* (Fig. 1C). The most recurrently mutated driver genes were *P2RY8* and *B2M*. *P2Y8* was mutated in 11/29 (38%) tumors, of which 7/11 (64%) had ≥ 2 mutations. *B2M* was mutated in 8/29 (28%) tumors, and five tumors had a CN loss on chromosome B3 encompassing *B2M* (three of the CN loss tumors also had a *B2M* mutation, suggesting biallelic inactivation; Fig. S5). The most recurrent CN loss was on chromosome D2, encompassing *PTEN* in 7/29 (24%) tumors, of which one also had a *PTEN* mutation, and *FAS* in 6/29 (21%) tumors, of which two also had a *FAS* mutation (Fig. S5 and Table S9). The most recurrent CN gain was on chromosome A2, encompassing *IKZF1* in 9/29 (31%) tumors (Fig. S5 and Table S9). There was overlap in the recurrently mutated genes between human diffuse large B-cell lymphoma (DLBCL) and our feline B-cell LYM cohort, including *TP53, B2M*, and *GNA13* (which were identified as driver genes of feline B-cell LYM). Considering CNA, further genetic similarities were observed between the species, including CN loss of *B2M, PTEN* and *FAS* (*119-121*). Human DLBCL can show differences in the mutational profile of some genes based on the site of origin, and we found that *P2RY8* mutations occurred more in the non-alimentary (8/11, 73%) than alimentary cases (n=3/11, 27%; Fig. S5), consistent with the incidence of *P2RY8* mutations being reduced in human gastro-intestinal (GI) DLBCL compared with non-GI DLBCL (*122*). Within the B-cell LYM cohort, it is worth noting that tumor CATD735a had a high TMB (41 mutations/megabase). Given the tumor had an elevated proportion of indels (~15%) and a *MLH1* mutation, it is likely mismatch repair deficiency (dMMR) is accounting for the elevated mutation rate. There is also a *POLE* p.V627A mutation, however, this is outside of the exonuclease domain, and the mutation rate is < 100 mutations/Mb, so it is unlikely that defective POLE proofreading is causing the elevated mutation rate. Although infrequent, dMMR has been reported in human DLBCL, though it does not have a prognostic impact (*123*).

In the T-cell cases (20 alimentary, 1 non-alimentary), no driver genes were identified. The most recurrently mutated gene was *JAK1* (n=3/21, 14%), with *STAT5B* mutated in 2/21 (9%) tumors (Fig. S6). Mutations in the JAK/STAT pathway are common in human intestinal T-cell lymphoma (which comprise mainly the enteropathy-associated T-cell lymphomas (EATL)), with mutations in *JAK1* and *STAT5B* occurring at frequencies of 14.7% and 26.5%, respectively (*124*). The most common alteration in feline T-cell LYM was a CN gain on chromosome F2 that encompassed *MYC* in 12/21 (57%) cases (Fig. S6 and Table S9). This is consistent with human

type II EATL (also known as monomorphic epitheliotropic intestinal T-cell lymphoma (MEITL), in which extra copies of *MYC* are commonly reported (*125*).

Key oncogenomic findings in the osteosarcomas

Osteosarcoma (OSA) is the most common primary bone tumor in cats and is broadly categorized by anatomical location, specifically appendicular, axial or extra-skeletal. Feline OSAs are considered aggressive tumors, however less so compared to dogs, with 30-40% cases showing distant metastasis (*126, 127*) and a post-surgical 2-year survival rate of only 55% (*127*). In our OSA cohort, we identified *TP53* as a driver gene (Fig. 1C); *TP53* was mutated in 8/25 (32%) cases, with an additional case having CN loss of a region on chr E1 spanning *TP53* (Fig. S7A).

The most recurrent CN gain was on chr F2, encompassing *MYC* in 8/25 (32%) tumors. In human OSA, *MYC* amplification is a clinical biomarker of poor outcome (*128*). The most recurrent CN loss was the proximal portion of chr D2, encompassing *PTEN*, *FAS*, and *ARID4B* (9/25, 36% tumors; Fig. S7 and Table S9). In human OSA, loss of *TP53* is considered an early tumorigenic event and loss of *PTEN* is involved in the later stages of tumor progression and metastasis (*129*). CN loss of *PTEN* is a driver event in canine OSA, observed in 25% cases, and CN loss of *FAS* has also been observed (*130*). In human OSA, *FAS* expression levels are inversely associated with metastatic potential (*131*).

We did not observe any differences in the oncogenomic landscape between tumors from appendicular or axial sites (Fig. S7A).

Key oncogenomic findings in lung tumors

Pulmonary neoplasia occurs infrequently in domestic animals, except for dogs and cats (*132*). The predominant histologic type of lung tumor in cats is adenocarcinoma (60-70% of cases), and feline pulmonary tumors have a more rapid progression and rate of metastasis than canine ones (metastasis seen in 75-80% of feline cases; reviewed in (*132*). In our lung carcinoma (LUCA) cohort, we identified *TP53* and *FGFR2* as driver genes (Fig. 1C), mutated in 22/57 (39%) and 5/57 (9%) cases, respectively, with biallelic loss of *TP53* seen in four cases due to somatic mutation of one allele and CN loss of the other (Fig. S8). In humans, loss of *TP53* (through mutation and/or CN loss) is one of the most common events in human lung cancer (~50% of non-small cell lung cancer (NSCLC) cases) and occurs early on in the development of the disease (*133*), whilst mutations in *FGFR3* are found in a subset of NSCLC cases (~3%) (*134*).

The most recurrent CN loss was on chr D4, encompassing *CDKN2A* and *TGFBR1* in 20/57 (35%) tumors and *NOTCH1* in 19/57 (33%) tumors (Fig. S8 and Table S9). Recurrent CN loss of the proximal portion of chr D2, encompassing *PTEN* and *FAS*, was observed in 9/57 (16%) tumors. CN loss of *CDKN2A* or *NOTCH1* are common events in human NSCLC (20-30% cases) (*135*). The most recurrent CN gain was on chr B2, encompassing a cluster of histone genes (*H1-2, 3, 4, 5, H2BC5/6*) in 18/57 (32%) tumors (Fig. S8 and Table S9). Recurrent CN gain on chr F2, encompassing *MYC*, was observed in 14/57 (25%) tumors. In humans, *H1.2* is highly expressed in NSCLC and promotes cancer cell growth (*136*), and *MYC* is overexpressed in >50% of NSCLC and is associated with therapeutic resistance (*137*).

An important difference between human NSCLC and feline LUCA is the mutational frequency of *EGFR*; frequently mutated in the former, yet not in the latter. However, *EGFR* mutations predominate in 'smoker' NSCLC, whereas *TP53* mutations predominate in 'non-smoker' NSCLC (*TP53* was a driver gene in feline LUCA). Thus, as only ~10% of human lung

cancer patients are 'non-smokers' (*138*), such mutations would not be strongly represented in human datasets.

Key oncogenomic findings in the skin tumors

Of the many different types of skin cancer that occur in cats, the most common are cutaneous squamous cell carcinoma (cSCC), basal cell carcinoma (BCC) and cutaneous mast cell tumor (cMCT).

Feline cSCC is a slow-growing invasive tumor that metastasizes late in disease, and its development has been linked to chronic UV radiation exposure and papillomavirus infection (*139*). The feline cSCC cohort showed a high TMB (Fig. 1B) which could be attributed to UV light exposure, as samples in which a COSMIC SBS7 mutational signature was identified (34/62, 52% of the cohort; Table S6 and Fig. S9) showed a higher TMB relative to those in which the signature was not identified (median TMB = 62.3 versus 1.25 mutations/Mb, respectively). Most tumors with an SBS7 signature were located on sun-exposed areas and where the hair is sparse or absent in DSH cats; on the pinna, 26/34 (76%), the nasal planum, 5/34 (14%), and the eyelid, 1/34 (3%; Table S2 and Fig. S9). The pinnae, nasal planum and eyelids of cats often show obvious actinic changes as the disease progresses from actinic keratosis to carcinoma *in situ* to cSCC (*140*), and together with the identification of signature SBS7 in the cSCC lesions, supports a role for UV sunlight in the etiology of this tumor type in cats. This shows parallels with human HNSCC, which is UV-associated (*141*). The high TMB of cSCC makes driver genes difficult to differentiate. However, we identified mutations in *TP53* (n=48/62, 77% tumors)*, NOTCH1* (n=21/62, 34% tumors) and *CDKN2A* (n=5/62, 8% tumors) as being under positive selection (Fig. S9), consistent with that seen in human cSCC (*142*). In contrast to the somatic mutational profile, the CNA landscape was relatively quiet, with the predominant events being a recurrent CN gain on chr F2, encompassing *MYC*, in 14/62 (22%) tumors and a recurrent CN loss of the proximal portion of chr D2, encompassing *PTEN* in 11/62 tumors and *FAS* in 10/62 tumors (~17%; Fig. S9 and Table S9). In human cSCC, *PTEN* inactivation (*142*) and *MYC* amplification (*143*) are common events.

BCC in cats typically presents on the head and neck area, and shows epidermal ulceration and local invasion, with only rare cases of metastasis reported (*68*). In our feline BCC cohort, *TP53* and *TGFBR1* were identified as driver genes (Fig. 1C), with biallelic inactivation seen in one case each (Fig. S10 and Table S9). *TP53* is one of the commonly mutated in human BCC (*144*). The most recurrent CNAs were a region of chr F2, encompassing *MYC*, in 8/40 (20%) tumors (Table S7), loss of the proximal portion of chr D2 encompassing *PTEN* in 7/40 (18%) tumors and *FAS* in 5/40 (12%) tumors (Fig. S10 and Table S9). These CNAs are not typically seen in human BCC. Indeed, whereas BCC in humans typically has a high TMB, is driven by UV exposure and >90% patients have mutations in the sonic hedgehog (SHH) pathway (*145*), in contrast, the SHH pathway is not a driver in feline BCC, it is not associated with UV exposure (no SBS7 mutational signatures detected and a relatively low TMB), and a potential viral etiology was observed which warrants further investigations.

cMCT in cats typically arises on the head and neck, followed by the trunk, and can occur as a solitary lesion/cluster of lesions or be widespread (*146*). The majority of feline cMCTs are benign, with excision or radiation generally being curative, however, ~10% show aggressive behavior (characterized by recurrence or lymph node metastasis) and are associated with a poorer prognosis (*146*). In our feline cMCT cohort, we identified *KIT* and *RARA* as driver genes. *KIT* mutations were found in 17/41 (41%) cases, and most occurred in exons 9 (12/17 tumors) and exon 8 (6/17 tumors), consistent with previous reports (*147, 148*). Two tumors had two mutations in

KIT, three tumors had a CN gain of a region of chr B1, encompassing *KIT*, and two tumors had a *KIT* mutation and CN gain of *KIT* (Fig. S11). The exon 8 and 9 KIT mutations seen in feline cMCT have also been reported in human pediatric patients with mastocytosis (*149*) and cutaneous mastocytosis (*150*). Importantly, there are case reports of the TKI imatinib showing clinical benefits in a child with KIT-mutated cutaneous mastocytosis (*150*), and a cat with exon 8 KIT-mutated MCT (*151*), highlighting the virtues of the One Medicine approach. *RARA* mutations were found in 2/41 (5%) cases. One tumor had two mutations in *RARA*, and two tumors had CN losses encompassing *RARA* (Table S9). The most recurrent CN gain was a region on chr B2, encompassing *MYB* (7/41 tumors, 17%), which is a gene important for mast cell differentiation (*152*), and the most recurrent CN loss was a region on chr D2, encompassing *ARID5B*, in 7/41 (17%) tumors (Fig. S11 and Table S9).

Key oncogenomic findings in the alimentary tract tumors

Oral squamous cell carcinoma (oSCC) is the most common oral neoplasm of cats. Feline oSCC is highly aggressive (>50% show invasion into the underlying bone), often causing necrosis of the surrounding tissues, and is generally associated with short survival times. Affected cats are typically euthanized due to local disease and its difficulty to resect, resulting in dysphagia, dyspnea or anorexia, rather than the development of clinically relevant metastases (reviewed in (*153*)). In our feline oSCC cohort, we identified *TP53* as a driver gene, with 12/42 (29%) cases having mutations in *TP53* and an additional two cases having a CN deletion spanning *TP53* (Fig. S12A). A key role for TP53 in feline oSCC has been suggested by previous studies, with one performing deep sequencing of exons 5-8 of *TP53* and finding mutations in 18/26 cases (*154*), and another performing whole-exome sequencing and finding *TP53* mutations in 4/6 cases (*17*). The most recurrent CNA was gain of a region of chr F2, encompassing numerous genes including *MYC* in 13/42 (31%) tumors, and loss of the proximal portion of chr D2 encompassing *PTEN* (10/42, 24% tumors) and *FAS* (9/42, 21% tumors; Fig. S12 and Table S9). It is worth noting that tumor CATD292a had the highest TMB of the sOCC cohort (52 muations/megabase), however, there were no mutations in any dMMR genes or *POLD1*/*POLE*, and no MNVs or indels. The tumor had a mutation in *ERCC2* (p.I251L), which functions in nucleotide excision repair (NER) DNA repair pathway, and in humans, *ERCC2* expression may be a risk factor for oSCC recurrence (*155*) and *ERCC2* germline polymorphisms have been associated with oSCC risk (*156*).

Intestinal neoplasia is common in cats, representing 5-9% of all feline neoplasms (*153*). Although lymphoma is by far the most common, adenocarcinomas have been reported to represent 28% of intestinal tumors (*153*). These tumors are most frequently reported in the small intestine (*157*). The most frequent histologic subtype in the large colon are adenocarcinomas (reviewed in (*153*)). Most feline intestinal adenocarcinomas (72-84%) show peritoneal or lymph node metastases at time of diagnosis, and even after surgical excision, the disease is associated with a poor prognosis (reviewed in (*153*)). In our feline adenocarcinoma cohort, we identified six driver genes, specifically *CTNNB1, APC, FBXW7, ACVR1B, MSH2* and *TRAF3*. The most frequently mutated driver gene was *CTNNB1* (13/34, 38% tumors), with all mutations occurring in exon 2 (Fig. S13). There was a hotspot at p.G34E/R (7/34, 21% tumors), consistent with reports from a study sequencing exon 2 of *CTNNB1* in feline intestinal carcinomas (mutations in 4/11, 36% cases) (*158*)). By comparison, although *CTNNB1* is recurrently mutated in human colon cancer (~7% cases), it is not considered a driver gene (*159*). Indeed, although APC was identified as a driver gene in feline CRC, it was only mutated in 9% cases, where >80% of human sporadic CRC have somatic *APC* mutations (*160*). Similarly, *TP53* and *KRAS* are driver genes of CRC in humans, but

in feline CRC (mutated in 5% of feline cases). The most recurrent CNA in the feline CRC cohort was a gain on chr F1, encompassing *PARP1*, in 3/34 (9%) tumors (Fig. S13 and Table S9). *PARP-1* overexpression is observed in human CRC and correlates with disease progression (*161*). It is worth noting that two tumors from the CRC cohort, CATD0463a and CATD0465a, had an elevated mutation rate (13 and 12 mutations/megabase, respectively), a large proportion of indels (~30% in each sample), and *MSH2* mutations in both samples. Together, these suggest that the elevated mutation rate is due to dMMR. Approximately 15% of human sporadic CRCs have dMMR (*162*).

Cholangiocarcioma (CCA) is uncommon in cats (<1% of all feline neoplasms) yet is the most common primary hepatic neoplasm. It is typically of intrahepatic origin and not generally associated with fluke infestations. It is generally associated with poor prognosis, as it is an aggressive tumor that tends to be scattered throughout the liver and is associated with high rates of metastasis (78% cases; reviewed in (*163*)). In our feline CCA cohort, we identified three driver genes, specifically *TP53, CTNNB1,* and *PTEN*. The most frequently altered driver gene was *TP53*, mutated in 12/30 (40%) tumors, and CN loss of *TP53* was found in four tumors (with one having both a mutation and CN deletion, suggesting biallelic inactivation; Fig. S14A). *TP53* is frequently mutated in human CCA and is a predictor of poor prognosis (*164*). Recurrent CNA seen in this cohort include gain of a region on chr F2, encompassing *MYC*, in 6/30 (20%) tumors, and loss of a region on chr, encompassing *ARID1A*, in 7/30 (23%) tumors, and the proximal portion of chr D2, encompassing *PTEN* and *FAS*, in 6/30 (20%) tumors (Fig. S14 and Table S9). In human CCA, *MYC* expression has been shown to mediate loss of contact inhibition (*165*), and loss of *ARID1A* expression has been reported in ~20% cases (*166*).

Pancreatic adenocarcinoma (PANC) is rare in cats and is an aggressive tumor with frequent extension into the small intestine. Distant metastasis (typically to the liver or lung) is seen in up to 80% of cases at diagnosis, and in general this tumor is associated with a poor prognosis (*167*). In our feline PANC cohort, we identified two driver genes, specifically *CTNNB1* (mutated in 15/29, 52% of tumors) and *TP53* (mutated in 9/29, 31% of tumors and in a region of CN loss in 2 tumors, one which also had a *TP53* mutation, suggesting biallelic inactivation; Fig. S15 and Table S9). In humans *TP53* is a driver gene of ductal pancreatic carcinoma (PDAC), with mutations in 50–90% of cases which have been shown to impact both prognosis and treatment response (*168*). Recurrent CNAs included loss of the proximal portion of chr D2, encompassing *PTEN* and *FAS* (6/29, 21% tumors), with one tumor also having a mutation in *PTEN* (suggesting biallelic inactivation), and gain of a region on chr F2, encompassing *MYC*, in 4/29 (14%) tumors (Fig. S15 and Table S9). Copy number loss of *PTEN* and gain of *MYC* are frequently observed in human PDAC (*169*). It is important to note that human PDAC is typically characterized by *KRAS* mutations (up to 95% of cases), with *KRAS* signaling essential for PDAC progression (*170*). Thus since PDAC represents ~90% of cases of human pancreatic cancer (*170*), it dominates the composition of pancreatic neoplasms in pan-cancer studies and *KRAS* mutations predominate human pancreatic cancer datasets (*171*). In contrast, pancreatic acinar cell carcinoma is rarer in humans (0.2-4.3% of pancreatic neoplasms), and thus not typically well-represented in pan-cancer studies. Yet the majority of PANCs in cats are of acinar cell origin (*172*), it is thus not surprising that *CTNNB1* was more frequently mutated in the feline PANC cohort, consistent with that seen in human pancreatic acinar cell carcinoma cohorts (*173*).

Key oncogenomic findings in the CNS tumors

Primary brain tumors have an incidence of ~2% in cats, with MEN being the most frequently reported. Meningioma (MEN) in cats are considered benign, slow-growing and typically well-circumscribed tumors. However, they have a 2-year survival rate of only 50%, despite surgical excision of the tumor (reviewed in (*174*)). In our feline MEN cohort, we identified *CTNNB1* as a driver gene, which was mutated in 4/28 (14%) tumors (Fig. S16). The most recurrent CNA was a gain on chr B1 encompassing *ADGRA2* in 6/28 (21%) tumors (Fig. S16 and Table S9). *CTNNB1* is rarely mutated in human MEN (*175*), rather the most common genetic aberration being functional loss of *NF2*, which is typically associated with poor prognosis (*176*). In *NF2*-wildtype MEN patients, which have better prognosis, ~25% of the cases have mutations in *TRAF7* (*176*). The feline MEN cohort was *NF2* wildtype (no mutations or CN loss of *NF2*), and 2/28 (7%) of the tumors had a mutation in *TRAF2*. Thus, feline MEN could potentially be a model of *NF2*-wildtype MEN in humans, although there is differing underlying biology in terms of signaling via the β-catenin pathway (which is rarely seen in human MEN).

GLIO (astrocytoma, oligodendroglioma, glioblastoma or ependymoma) are exceedingly rare in cats, with one study reporting only 13 cases between two veterinary diagnostic institutions in the USA over a 16-year period and it is difficult to draw conclusions from the clinical data as most cats are euthanized (*177*). In our feline GLIO cohort, we identified four driver genes, specifically *PTPN11, PDGFR1, PTPRC,* and *NF1*. The most recurrently mutated driver genes were *PDGFRA* (mutated in 4/7, 57% of tumors) and *PTPN11* (mutated in 3/7, 43% of tumors; Fig. S17). *PTPN11* mutations are rare in human gliomas (*178*), however, *PDGFRA* mutations and/or amplifications have been reported in 15% of high-grade gliomas (*179*) and a high rate of somatic *NF1* mutation has been observed in glioblastomas (*180*). The most recurrent CNAs gains were on chr A2, encompassing *PTPRS*, and chr D2, encompassing *PGBD5*, in 3/7 (42%) tumors each. Loss of a proximal region on chr D2 encompassing *PTEN, FAS* and *ARID4B*, occurred in 2/7 (29%) tumors; one tumor had a concomitant mutation in *PTEN,* and one had a concomitant mutation in *FAS*, suggesting biallelic inactivation; Fig. S17 and Table S9). CN loss of *PTEN* is commonly observed in human gliomas (*36*).

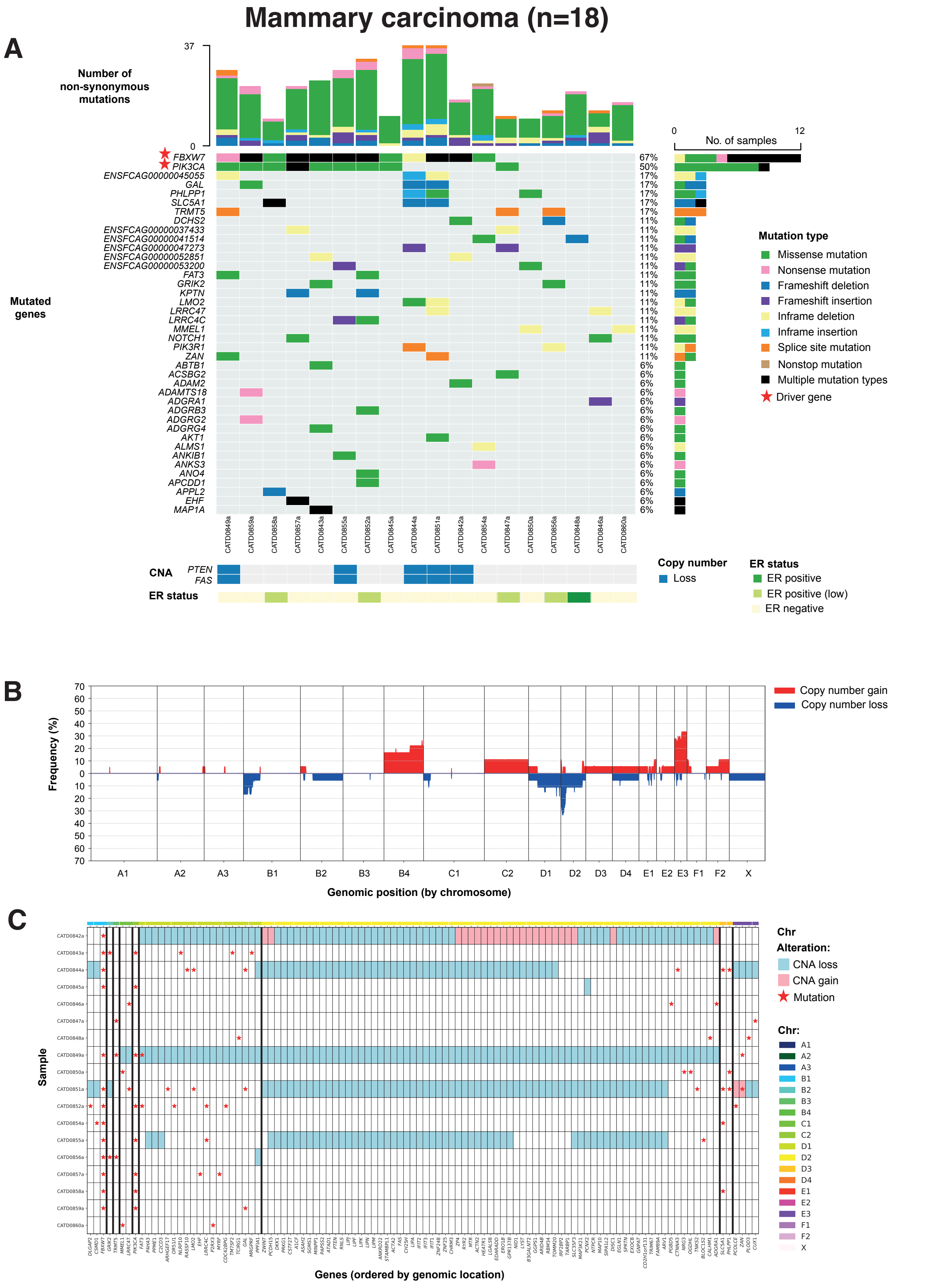
Mammary carcinoma (n=18)
A
Number of non-synonymous mutations
37
0
Mutated genes
FBXW7
PIK3CA
ENSFCAG00000045055
GAL
PHLPP1
SLC5A1
TRMT5
DCHS2
ENSFCAG00000037433
ENSFCAG00000041514
ENSFCAG00000047273
ENSFCAG00000052851
ENSFCAG00000053200
FAT3
GRIK2
KPTN
LMO2
LRRC47
LRRC4C
MMEL1
NOTCH1
PIK3R1
ZAN
ABTB1
ACSBG2
ADAM2
ADAMTS18
ADGRA1
ADGRB3
ADGRG2
ADGRG4
AKT1
ALMS1
ANKIB1
ANKS3
ANO4
APCDD1
APPL2
EHF
MAP1A
67%
50%
17%
11%
6%
CATD0849a
CATD0859a
CATD0858a
CATD0857a
CATD0843a
CATD0855a
CATD0852a
CATD0845a
CATD0844a
CATD0851a
CATD0842a
CATD0854a
CATD0847a
CATD0850a
CATD0856a
CATD0848a
CATD0846a
CATD0860a
0
12
No. of samples
Mutation type
Missense mutation
Nonsense mutation
Frameshift deletion
Frameshift insertion
Inframe deletion
Inframe insertion
Splice site mutation
Nonstop mutation
Multiple mutation types
Driver gene
CNA
PTEN
FAS
ER status
Copy number
Loss
ER status
ER positive
ER positive (low)
ER negative
B
Frequency (%)
70
60
50
40
30
20
10
0
A1
A2
A3
B1
B2
B3
B4
C1
C2
D1
D2
D3
D4
E1
E2
E3
F1
F2
X
Genomic position (by chromosome)
Copy number gain
Copy number loss
C
Sample
Genes (ordered by genomic location)
Chr
Alteration:
CNA loss
CNA gain
Mutation
Chr:

Fig. S1. The mutational landscape of cancer-associated genes in feline mammary carcinoma. (**A**) Oncoplot showing mutations present in the top 40 mutated genes (defined as recurrently mutated genes, listed in alphabetical order, followed by genes mutated in a single sample, listed in alphabetical order, if the maximum of 40 genes was not reached). The full list of protein-altering mutations identified within each sample is in Table S3. Driver genes were *FBXW7* (q-global: $<1x10^{-16}$) and *PIK3CA* (q-global: $2.6x10^{-7}$). (**B**) Frequency plot showing the copy number alterations (CNA) across the genome (at a chromosome level). Copy number (CN) gains (amplifications; log2 $\geq$ +0.32) are shown in red, while CN losses (deletions; log2 $\leq$ -0.40) are shown in blue. (**C**) Oncoplot showing the CNA landscape, overlaid with the somatic mutations. A star indicates at least one protein-altering SNV/MNV/indel in that gene. The plot is filtered to only show genes with $\geq$ 3 alterations (either mutations or copy number alterations).

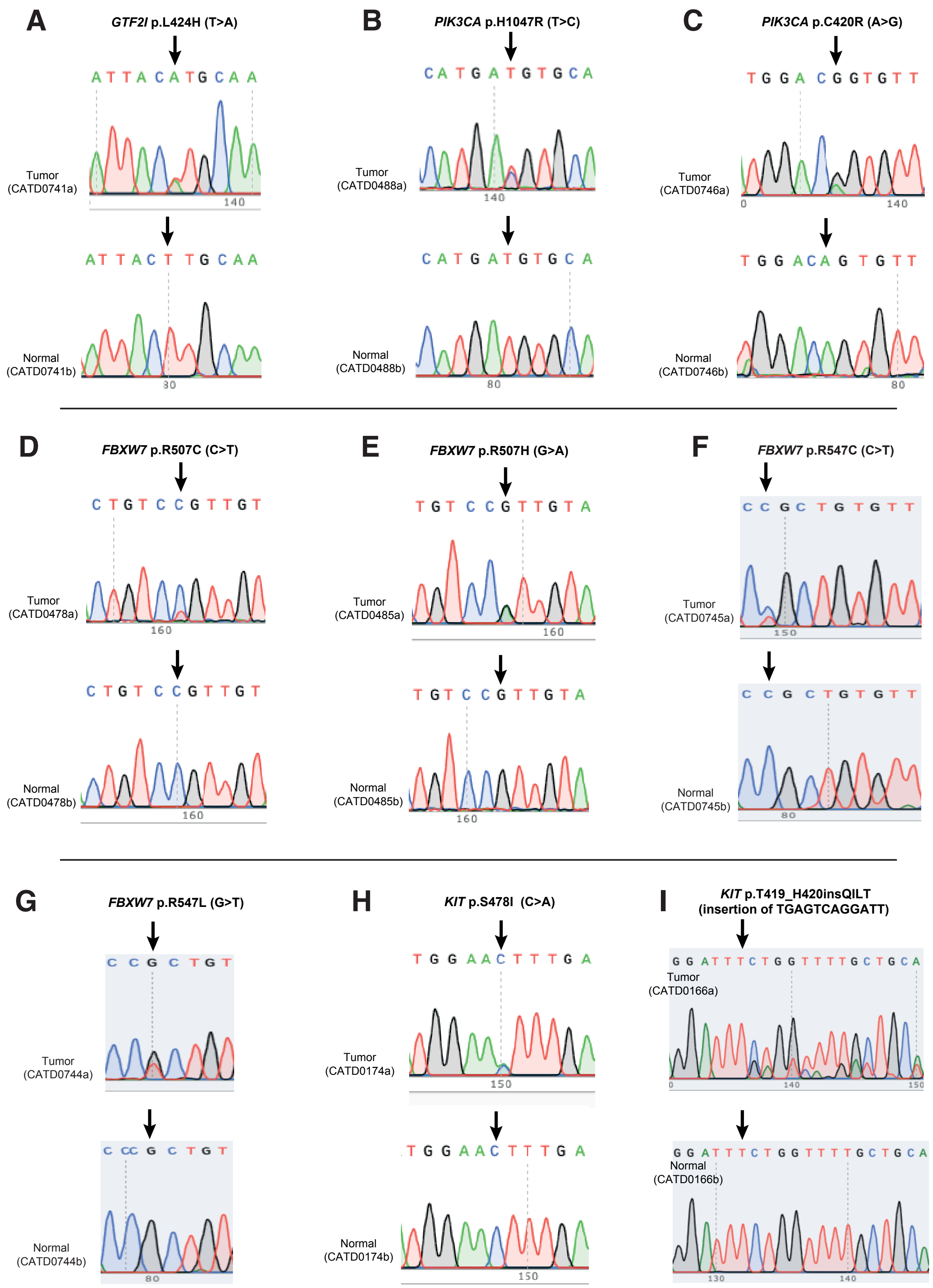
A
GTF2I p.L424H (T>A)
Tumor (CATD0741a)
Normal (CATD0741b)
B
PIK3CA p.H1047R (T>C)
Tumor (CATD0488a)
Normal (CATD0488b)
C
PIK3CA p.C420R (A>G)
Tumor (CATD0746a)
Normal (CATD0746b)
D
FBXW7 p.R507C (C>T)
Tumor (CATD0478a)
Normal (CATD0478b)
E
FBXW7 p.R507H (G>A)
Tumor (CATD0485a)
Normal (CATD0485b)
F
FBXW7 p.R547C (C>T)
Tumor (CATD0745a)
Normal (CATD0745b)
G
FBXW7 p.R547L (G>T)
Tumor (CATD0744a)
Normal (CATD0744b)
H
KIT p.S478I (C>A)
Tumor (CATD0174a)
Normal (CATD0174b)
I
KIT p.T419_H420insQILT (insertion of TGAGTCAGGATT)
Tumor (CATD0166a)
Normal (CATD0166b)

Fig. S2. Orthogonal validation of the somatic hotspots in the feline mammary carcinoma and cutaneous mast cell tumor cohorts. For each somatic hotspot that was validated by Sanger sequencing, a representative sequence trace is shown for both the tumor and its matched normal. For mammary carcinoma, the five hotspots were: (**A**) *GTF2I* p.L424H (ENSFCAG00000002617, E3:11080806, T>A), validated in 2/2 cases; (**B**) *PIK3CA* p.H1047R (ENSFCAG00000018737, C2: 89915434, T>C) validated in 7/7 cases; (**C**) *PIK3CA* p.C420R (ENSFCAG00000018737, C2: 89935963, A>G) validated in 2/2 cases; (**D-E**) *FBXW7* p.R507C/H (ENSFCAG00000000620, B1: 77324728/9, C>T or G>A) validated in 4/4 cases; (**F-G**) *FBXW7* p.R547C/L (ENSFCAG00000000620, B1: 77326876/7, C>T or G>T), validated in 2/2 cases. For cutaneous mast cell tumor, the two hotspots were: (**H**) *KIT* p.S478I (ENSFCAG00000003112, B1: 163967276, C>A) and (**I**) *KIT* p.T419_H420insQILT (ENSFCAG00000003112, B1: 163968990, insertion of TGAGTCAGGATT), validated in 4/4 cases each.

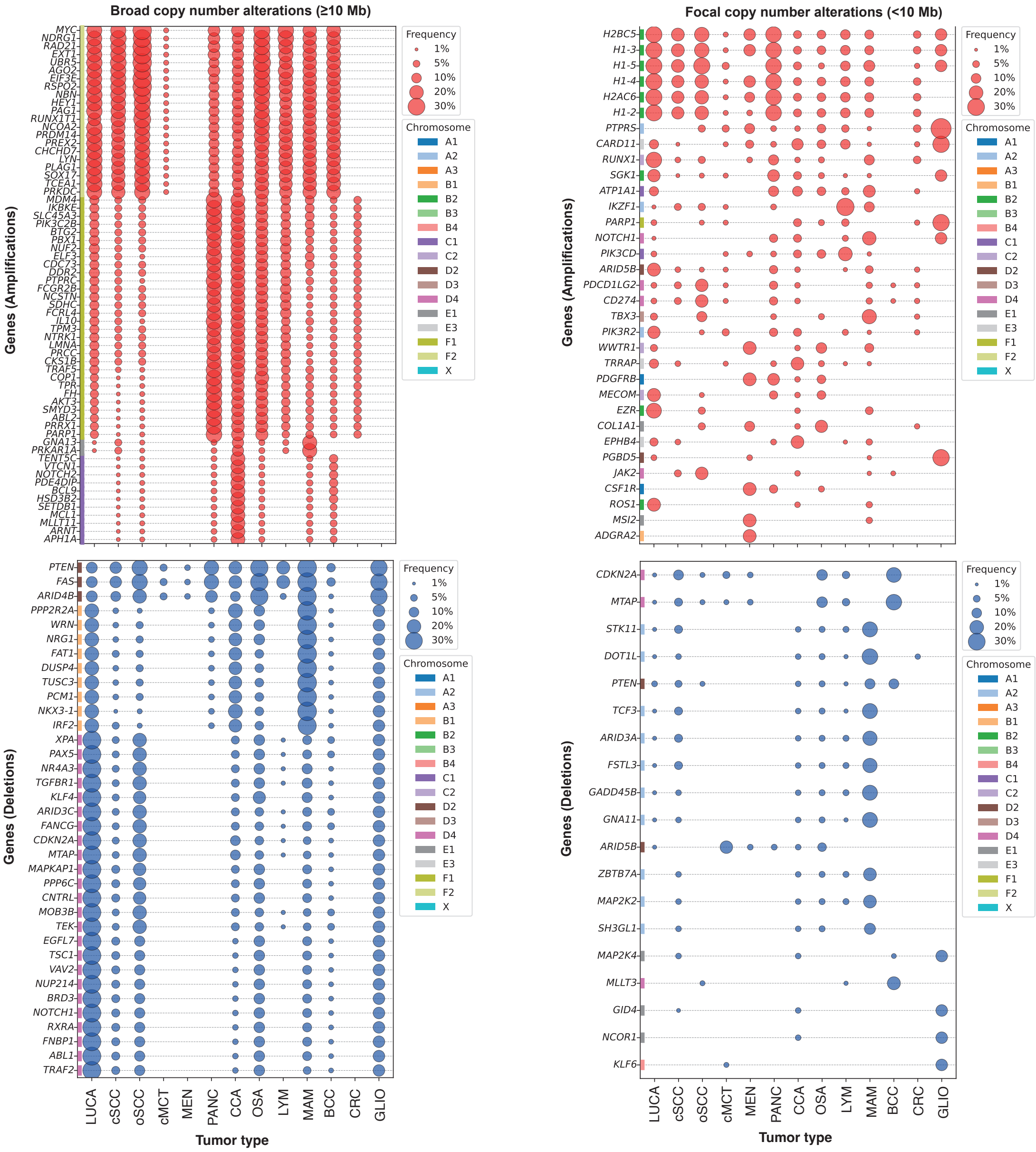
Broad copy number alterations (≥10 Mb)
Focal copy number alterations (<10 Mb)
Genes (Amplifications)
Genes (Deletions)
Frequency
1%
5%
10%
20%
30%
Chromosome
A1
A2
A3
B1
B2
B3
B4
C1
C2
D2
D3
D4
E1
E3
F1
F2
X
MYC
NDRG1
RAD21
EXT1
UBR5
AGO2
EIF3E
RSPO2
NBN
HEY1
PAG1
RUNX1T1
NCOA2
PRDM14
PREX2
CHCHD7
LYN
PLAG1
SOX17
TCEA1
PRKDC
MDM4
IKBKE
SLC45A3
PIK3C2B
BTG2
PBX1
NUF2
ELF3
CDC73
DDR2
PTPRC
FCGR2B
NCSTN
SDHC
FCRL4
IL10
TPM3
NTRK1
LMNA
PRCC
CKS1B
TRAF5
COP1
TPR
FH
AKT3
SMYD3
ABL2
PRRX1
PARP1
GNA13
PRKAR1A
TENT5C
VTCN1
NOTCH2
PDE4DIP
BCL9
HSD3B2
SETDB1
MCL1
MLLT11
ARNT
APH1A
PTEN
FAS
ARID4B
PPP2R2A
WRN
NRG1
FAT1
DUSP4
TUSC3
PCM1
NKX3-1
IRF2
XPA
PAX5
NR4A3
TGFBR1
KLF4
ARID3C
FANCG
CDKN2A
MTAP
MAPKAP1
PPP6C
CNTRL
MOB3B
TEK
EGFL7
TSC1
VAV2
NUP214
BRD3
NOTCH1
RXRA
FNBP1
ABL1
TRAF2
H2BC5
H1-3
H1-5
H1-4
H2AC6
H1-2
PTPRS
CARD11
RUNX1
SGK1
ATP1A1
IKZF1
PARP1
NOTCH1
PIK3CD
ARID5B
PDCD1LG2
CD274
TBX3
PIK3R2
WWTR1
TRRAP
PDGFRB
MECOM
EZR
COL1A1
EPHB4
PGBD5
JAK2
CSF1R
ROS1
MSI2
ADGRA2
CDKN2A
MTAP
STK11
DOT1L
PTEN
TCF3
ARID3A
FSTL3
GADD45B
GNA11
ARID5B
ZBTB7A
MAP2K2
SH3GL1
MAP2K4
MLLT3
GID4
NCOR1
KLF6
LUCA
cSCC
oSCC
cMCT
MEN
PANC
CCA
OSA
LYM
MAM
BCC
CRC
GLIO
Tumor type

Fig. S3. Copy number alterations in feline tumors. Bubble plots illustrating the frequency of both broad (≥10Mb) and focal (<10Mb) copy number (CN) alterations across multiple feline tumor types. CN gains (amplifications; log2 ≥ 0.32) are shown in red, while CN losses (deletions; log2 ≤ -0.40) are shown in blue. Shown on the plots are broad amplifications with a frequency ≥ 20%, broad deletions with a frequency ≥ 33%, focal amplifications with a frequency ≥ 15%, and focal deletions with a frequency ≥ 10%. Genes associated with each CN alteration are shown on the y-axis, and the tumor types are displayed across the x-axis. The relative size of each bubble indicates the frequency of the CN alteration in each tumor type. The chromosomal location of the affected genes is indicated by the color bars on the y-axis. Tumor type abbreviations: BCC, basal cell carcinoma; CCA, cholangiocarcinoma; CRC, colorectal adenocarcinoma; GLIO, glioma; LUCA, lung adenocarcinoma; LYM, lymphoma; MAM, mammary carcinoma; cMCT, cutaneous mast cell tumor; MEN, meningioma; OSA, osteosarcoma; PANC, pancreatic adenocarcinoma; cSCC, cutaneous squamous cell carcinoma; oSCC, oral squamous cell carcinoma.

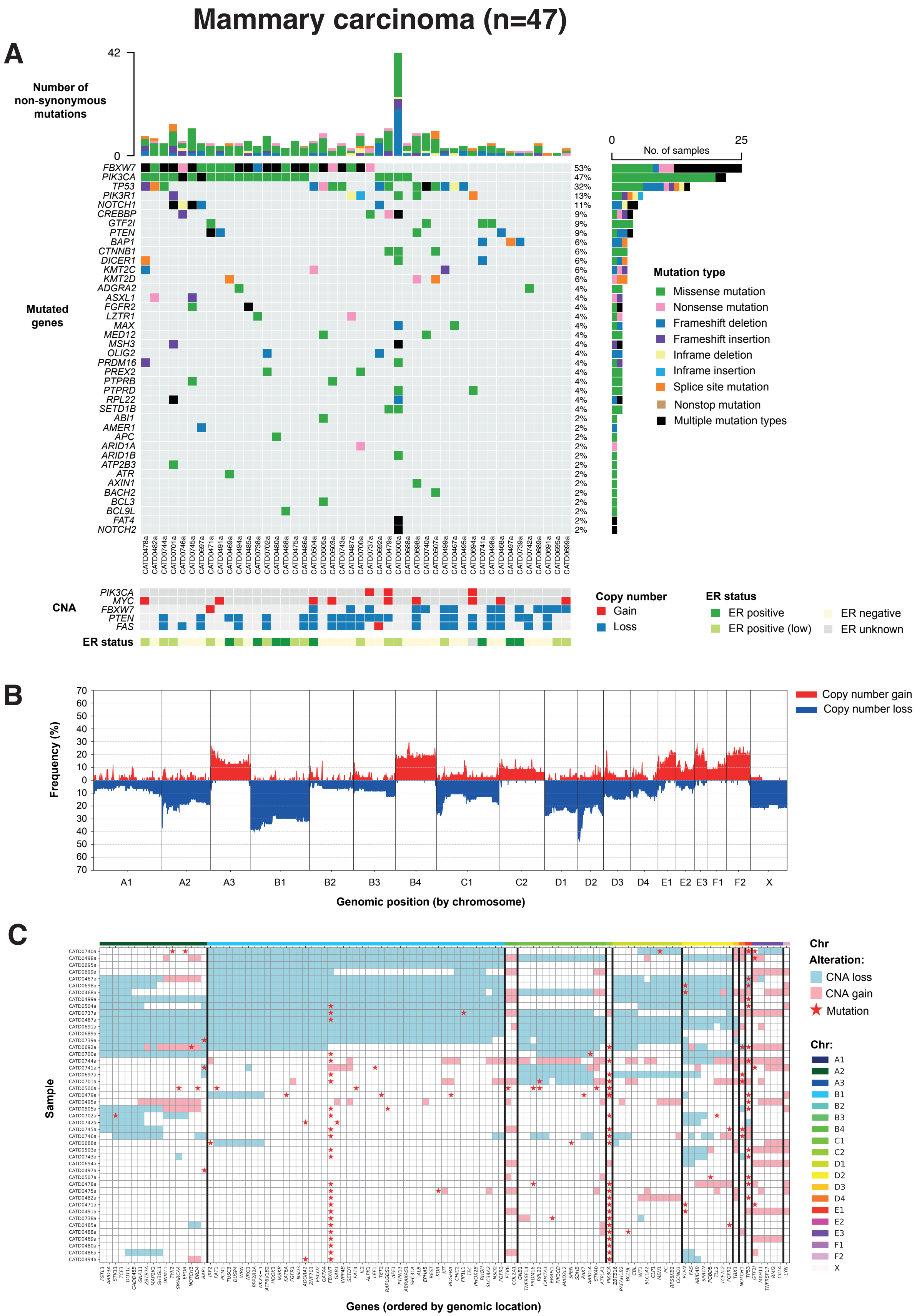
Mammary carcinoma (n=47)
A
Number of non-synonymous mutations
42
0
25
No. of samples
FBXW7
PIK3CA
TP53
PIK3R1
NOTCH1
CREBBP
GTF2I
PTEN
BAP1
CTNNB1
DICER1
KMT2C
KMT2D
ADGRA2
ASXL1
FGFR2
LZTR1
MAX
MED12
MSH3
OLIG2
PRDM16
PREX2
PTPRB
PTPRD
RPL22
SETD1B
ABI1
AMER1
APC
ARID1A
ARID1B
ATP2B3
ATR
AXIN1
BACH2
BCL3
BCL9L
FAT4
NOTCH2
53%
47%
32%
13%
11%
9%
9%
9%
6%
6%
6%
6%
6%
4%
4%
4%
4%
4%
4%
4%
4%
4%
4%
4%
4%
4%
4%
2%
2%
2%
2%
2%
2%
2%
2%
2%
2%
2%
2%
2%
Mutated genes
Mutation type
Missense mutation
Nonsense mutation
Frameshift deletion
Frameshift insertion
Inframe deletion
Inframe insertion
Splice site mutation
Nonstop mutation
Multiple mutation types
CNA
PIK3CA
MYC
FBXW7
PTEN
FAS
ER status
Copy number
Gain
Loss
ER status
ER positive
ER positive (low)
ER negative
ER unknown
B
Frequency (%)
Copy number gain
Copy number loss
Genomic position (by chromosome)
C
Sample
Chr
Alteration:
CNA loss
CNA gain
Mutation
Chr:
A1
A2
A3
B1
B2
B3
B4
C1
C2
D1
D2
D3
D4
E1
E2
E3
F1
F2
X
Genes (ordered by genomic location)

Fig. S4. The mutational landscape of cancer-associated genes in feline mammary carcinoma. (**A**) Oncoplot showing mutations present in the top 40 mutated genes (defined as recurrently mutated genes, listed in alphabetical order, followed by genes mutated in a single sample, listed in alphabetical order, if the maximum of 40 genes was not reached). The full list of protein-altering mutations identified within each sample is in Table S3. Tumors without any protein-altering mutations are not shown in the plot (but are accounted for in the frequency percentages shown). (**B**) Frequency plot showing the copy number alterations (CNA) across the genome (at a chromosome level). Copy number (CN) gains (amplifications; log2 ≥ +0.32) are shown in red, while CN losses (deletions; log2 ≤ -0.40) are shown in blue. (**C**) Oncoplot showing the CNA landscape (at a gene level), overlaid with the somatic mutations. A star indicates at least one protein-altering SNV/MNV/indel in that gene. The plot is filtered to only show genes with ≥ 12 alterations (either mutations or copy number alterations). Tumors are included only if they have at least one alteration in this filtered gene list.

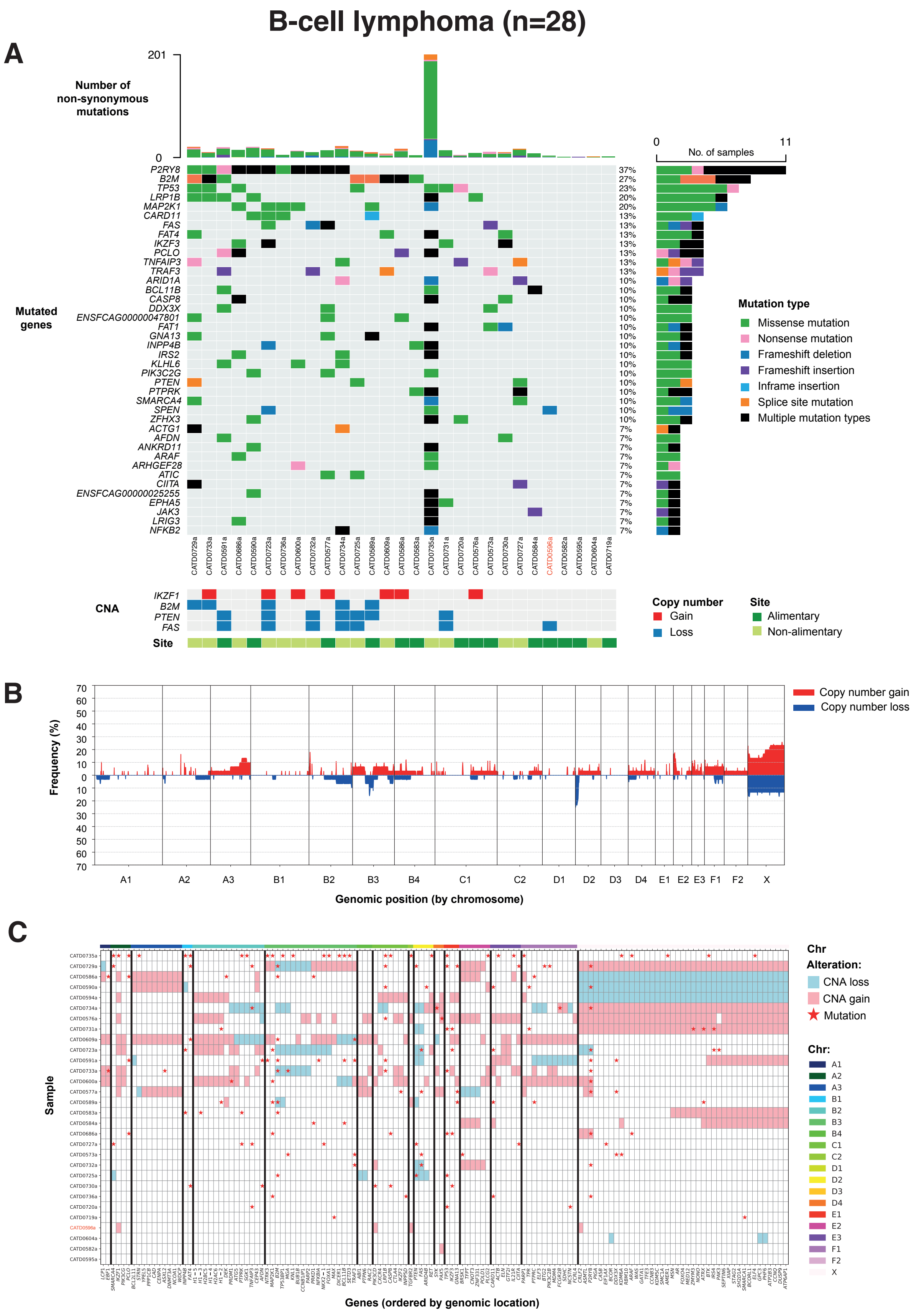
B-cell lymphoma (n=28)
A
Number of non-synonymous mutations
Mutated genes
No. of samples
Mutation type
Missense mutation
Nonsense mutation
Frameshift deletion
Frameshift insertion
Inframe insertion
Splice site mutation
Multiple mutation types
CNA
Site
Copy number
Gain
Loss
Alimentary
Non-alimentary
B
Frequency (%)
Copy number gain
Copy number loss
Genomic position (by chromosome)
C
Sample
Chr
Alteration:
CNA loss
CNA gain
Mutation
Genes (ordered by genomic location)

Fig. S5. The mutational landscape of cancer-associated genes in feline B-cell lymphoma. (**A**) Oncoplot showing mutations present in the top 40 mutated genes (defined as recurrently mutated genes, listed in alphabetical order, followed by genes mutated in a single sample, listed in alphabetical order, if the maximum of 40 genes was not reached). The full list of protein-altering mutations identified within each sample is in Table S3. Tumors without any protein-altering mutations are not shown in the plot (but are accounted for in the frequency percentages shown). The sample in red font is the NK-cell lymphoma case (shown for comparative purposes). (**B**) Frequency plot showing the copy number alterations (CNA) across the genome (at a chromosome level). Copy number (CN) gains (amplifications; log2 $\geq$ +0.32) are shown in red, while CN losses (deletions; log2 $\leq$ -0.40) are shown in blue. (**C**) Oncoplot showing the CNA landscape, overlaid with the somatic mutations. A star indicates at least one protein-altering SNV/MNV/indel in that gene. The plot is filtered to only show genes with $\geq$ 4 alterations (either mutations or copy number alterations). Tumors are included only if they have at least one alteration in this filtered gene list. The sample in red font is the NK-cell lymphoma case (shown for comparative purposes).

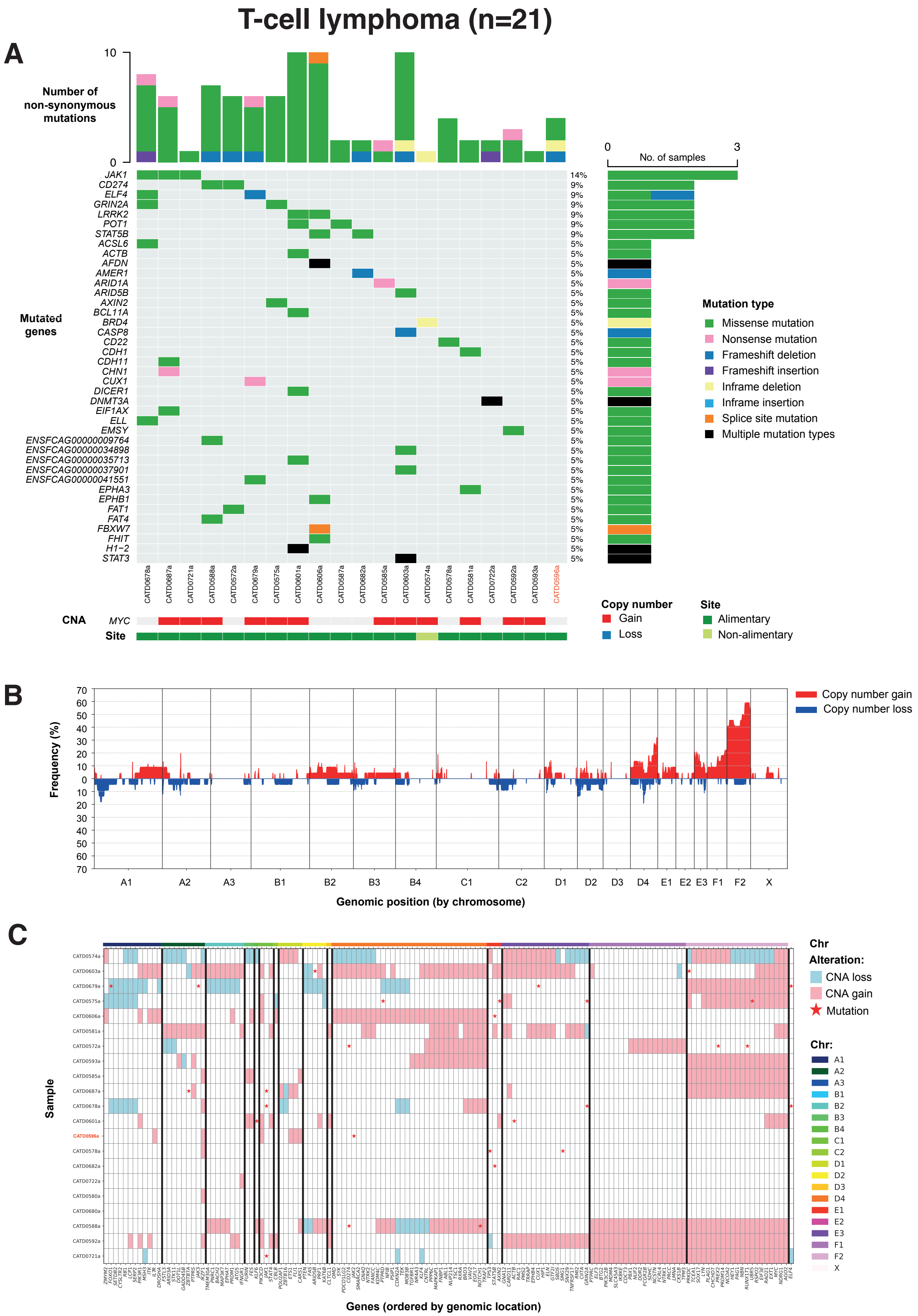
T-cell lymphoma (n=21)
A
Number of non-synonymous mutations
10
0
No. of samples
3
Mutated genes
JAK1
CD274
ELF4
GRIN2A
LRRK2
POT1
STAT5B
ACSL6
ACTB
AFDN
AMER1
ARID1A
ARID5B
AXIN2
BCL11A
BRD4
CASP8
CD22
CDH1
CDH11
CHN1
CUX1
DICER1
DNMT3A
EIF1AX
ELL
EMSY
ENSFCAG00000009764
ENSFCAG00000034898
ENSFCAG00000035713
ENSFCAG00000037901
ENSFCAG00000041551
EPHA3
EPHB1
FAT1
FAT4
FBXW7
FHIT
H1−2
STAT3
14%
9%
5%
Mutation type
Missense mutation
Nonsense mutation
Frameshift deletion
Frameshift insertion
Inframe deletion
Inframe insertion
Splice site mutation
Multiple mutation types
CATD0678a
CATD0687a
CATD0721a
CATD0588a
CATD0572a
CATD0679a
CATD0575a
CATD0601a
CATD0606a
CATD0587a
CATD0682a
CATD0585a
CATD0603a
CATD0574a
CATD0578a
CATD0581a
CATD0722a
CATD0592a
CATD0593a
CATD0596a
CNA
MYC
Site
Copy number
Gain
Loss
Site
Alimentary
Non-alimentary
B
Frequency (%)
Copy number gain
Copy number loss
A1 A2 A3 B1 B2 B3 B4 C1 C2 D1 D2 D3 D4 E1 E2 E3 F1 F2 X
Genomic position (by chromosome)
C
Sample
Chr
Alteration:
CNA loss
CNA gain
Mutation
Chr:
A1 A2 A3 B1 B2 B3 B4 C1 C2 D1 D2 D3 D4 E1 E2 E3 F1 F2 X
Genes (ordered by genomic location)

Fig. S6. The mutational landscape of cancer-associated genes in feline T-cell lymphoma. (**A**) Oncoplot showing mutations present in the top 40 mutated genes (defined as recurrently mutated genes, listed in alphabetical order, followed by genes mutated in a single sample, listed in alphabetical order, if the maximum of 40 genes was not reached). The full list of protein-altering mutations identified within each sample is in Table S3. Tumors without any protein-altering mutations are not shown in the plot (but are accounted for in the frequency percentages shown). The sample in red font is the NK-cell lymphoma case (shown for comparative purposes). (**B**) Frequency plot showing the copy number alterations (CNA) across the genome (at a chromosome level). Copy number (CN) gains (amplifications; $\log2 \geq +0.32$) are shown in red, while CN losses (deletions; $\log2 \leq -0.40$) are shown in blue. (**C**) Oncoplot showing the CNA landscape, overlaid with the somatic mutations. A star indicates at least one protein-altering SNV/MNV/indel in that gene. The plot is filtered to only show genes with ≥ 4 alterations (either mutations or copy number alterations). Tumors are included only if they have at least one alteration in this filtered gene list. The sample in red font is the NK-cell lymphoma case (shown for comparative purposes).

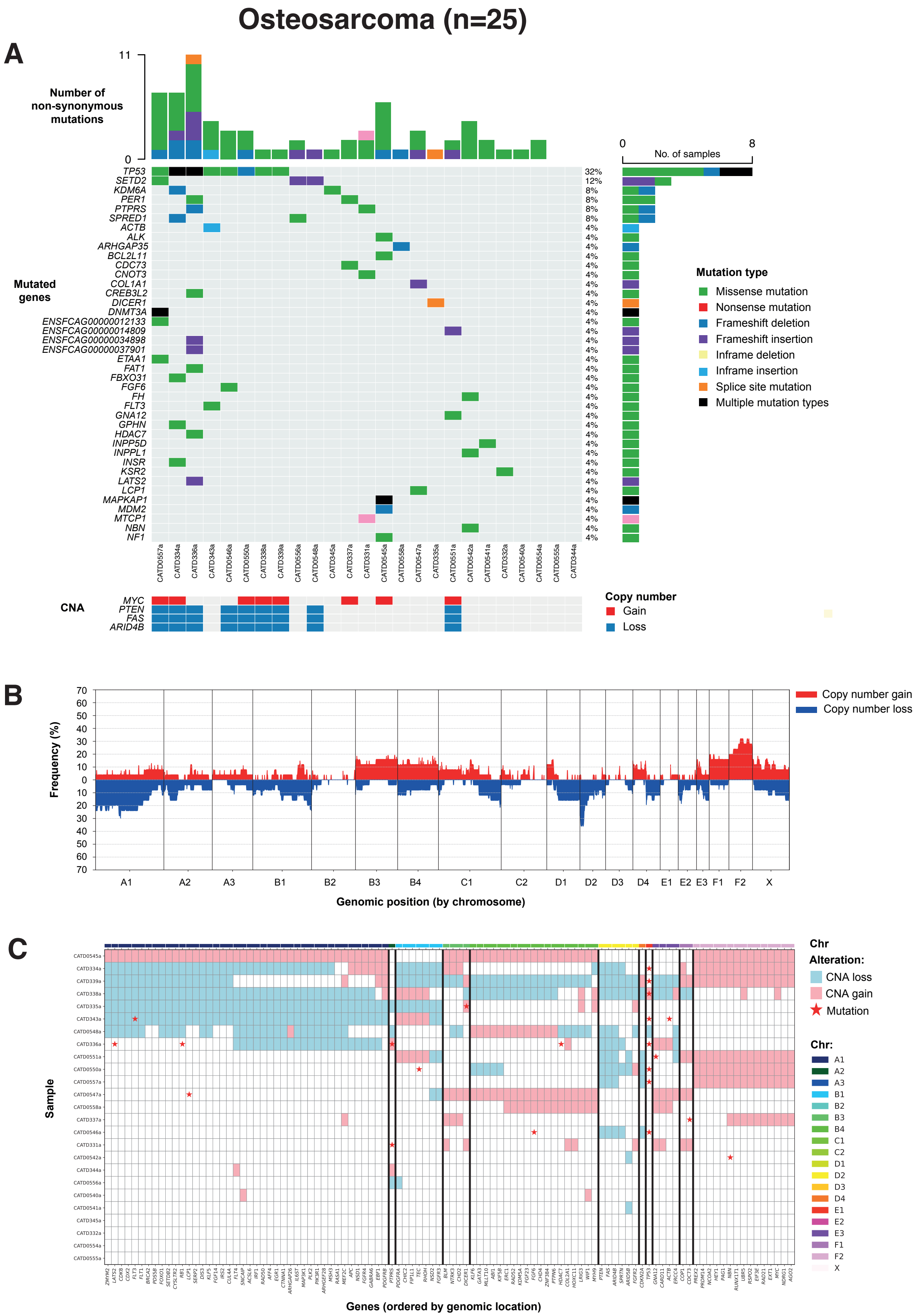
Osteosarcoma (n=25)
A
Number of non-synonymous mutations
Mutated genes
No. of samples
Mutation type
Missense mutation
Nonsense mutation
Frameshift deletion
Frameshift insertion
Inframe deletion
Inframe insertion
Splice site mutation
Multiple mutation types
CNA
Copy number
Gain
Loss
B
Frequency (%)
Copy number gain
Copy number loss
Genomic position (by chromosome)
C
Sample
Chr
Alteration:
CNA loss
CNA gain
Mutation
Genes (ordered by genomic location)

Fig. S7. The mutational landscape of cancer-associated genes in feline osteosarcoma. (**A**) Oncoplot showing mutations present in the top 40 mutated genes (defined as recurrently mutated genes, listed in alphabetical order, followed by genes mutated in a single sample, listed in alphabetical order, if the maximum of 40 genes was not reached). The full list of protein-altering mutations identified within each sample is in Table S3. Tumors without any protein-altering mutations are not shown in the plot (but are accounted for in the frequency percentages shown). (**B**) Frequency plot showing the copy number alterations (CNA) across the genome (at a chromosome level). Copy number (CN) gains (amplifications; log2 $\geq$ +0.32) are shown in red, while CN losses (deletions; log2 $\leq$ -0.40) are shown in blue. (**C**) Oncoplot showing the CNA landscape, overlaid with the somatic mutations. A star indicates at least one protein-altering SNV/MNV/indel in that gene. The plot is filtered to only show genes with $\geq$ 5 alterations (either mutations or copy number alterations). Tumors are included only if they have at least one alteration in this filtered gene list.

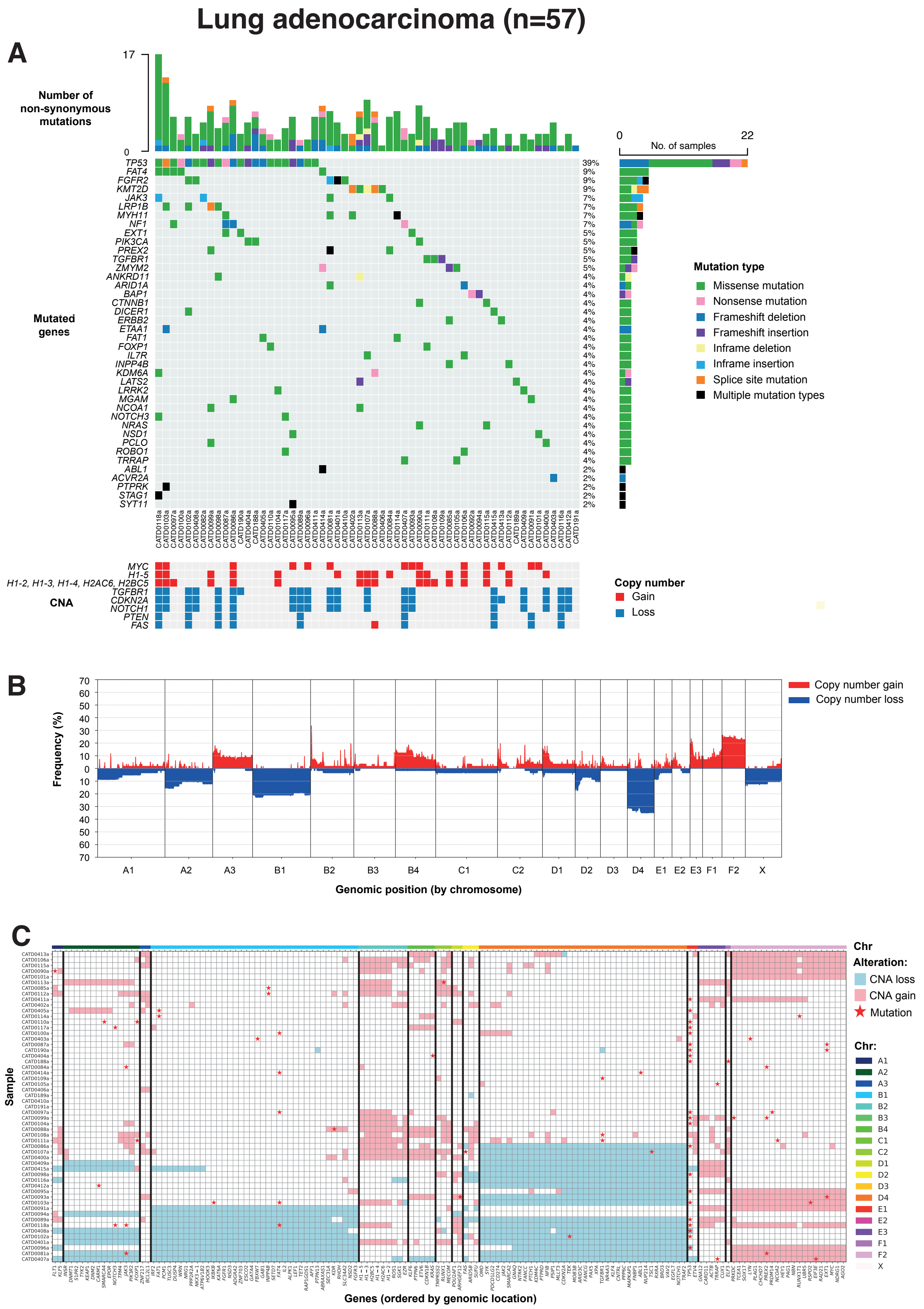
Lung adenocarcinoma (n=57)
A
Number of non-synonymous mutations
17
0
Mutated genes
TP53
FAT4
FGFR2
KMT2D
JAK3
LRP1B
MYH11
NF1
EXT1
PIK3CA
PREX2
TGFBR1
ZMYM2
ANKRD11
ARID1A
BAP1
CTNNB1
DICER1
ERBB2
ETAA1
FAT1
FOXP1
IL7R
INPP4B
KDM6A
LATS2
LRRK2
MGAM
NCOA1
NOTCH3
NRAS
NSD1
PCLO
ROBO1
TRRAP
ABL1
ACVR2A
PTPRK
STAG1
SYT11
39%
9%
7%
5%
4%
2%
No. of samples
0
22
Mutation type
Missense mutation
Nonsense mutation
Frameshift deletion
Frameshift insertion
Inframe deletion
Inframe insertion
Splice site mutation
Multiple mutation types
CNA
MYC
H1-5
H1-2, H1-3, H1-4, H2AC6, H2BC5
TGFBR1
CDKN2A
NOTCH1
PTEN
FAS
Copy number
Gain
Loss
B
Frequency (%)
Copy number gain
Copy number loss
Genomic position (by chromosome)
A1 A2 A3 B1 B2 B3 B4 C1 C2 D1 D2 D3 D4 E1 E2 E3 F1 F2 X
C
Sample
Genes (ordered by genomic location)
Chr
Alteration:
CNA loss
CNA gain
Mutation
Chr:
A1
A2
A3
B1
B2
B3
B4
C1
C2
D1
D2
D3
D4
E1
E2
E3
F1
F2
X

Fig. S8. The mutational landscape of cancer-associated genes in feline lung adenocarcinoma. (**A**) Oncoplot showing mutations present in the top 40 mutated genes (defined as recurrently mutated genes, listed in alphabetical order, followed by genes mutated in a single sample, listed in alphabetical order, if the maximum of 40 genes was not reached). The full list of protein-altering mutations identified within each sample is in Table S3. Tumors without any protein-altering mutations are not shown in the plot (but are accounted for in the frequency percentages shown). (**B**) Frequency plot showing the copy number alterations (CNA) across the genome (at a chromosome level). Copy number (CN) gains (amplifications; $\log2 \geq +0.32$) are shown in red, while CN losses (deletions; $\log2 \leq -0.40$) are shown in blue. (**C**) Oncoplot showing the CNA landscape, overlaid with the somatic mutations. A star indicates at least one protein-altering SNV/MNV/indel in that gene. The plot is filtered to only show genes with ≥ 10 alterations (either mutations or copy number alterations). Tumors are included only if they have at least one alteration in this filtered gene list.

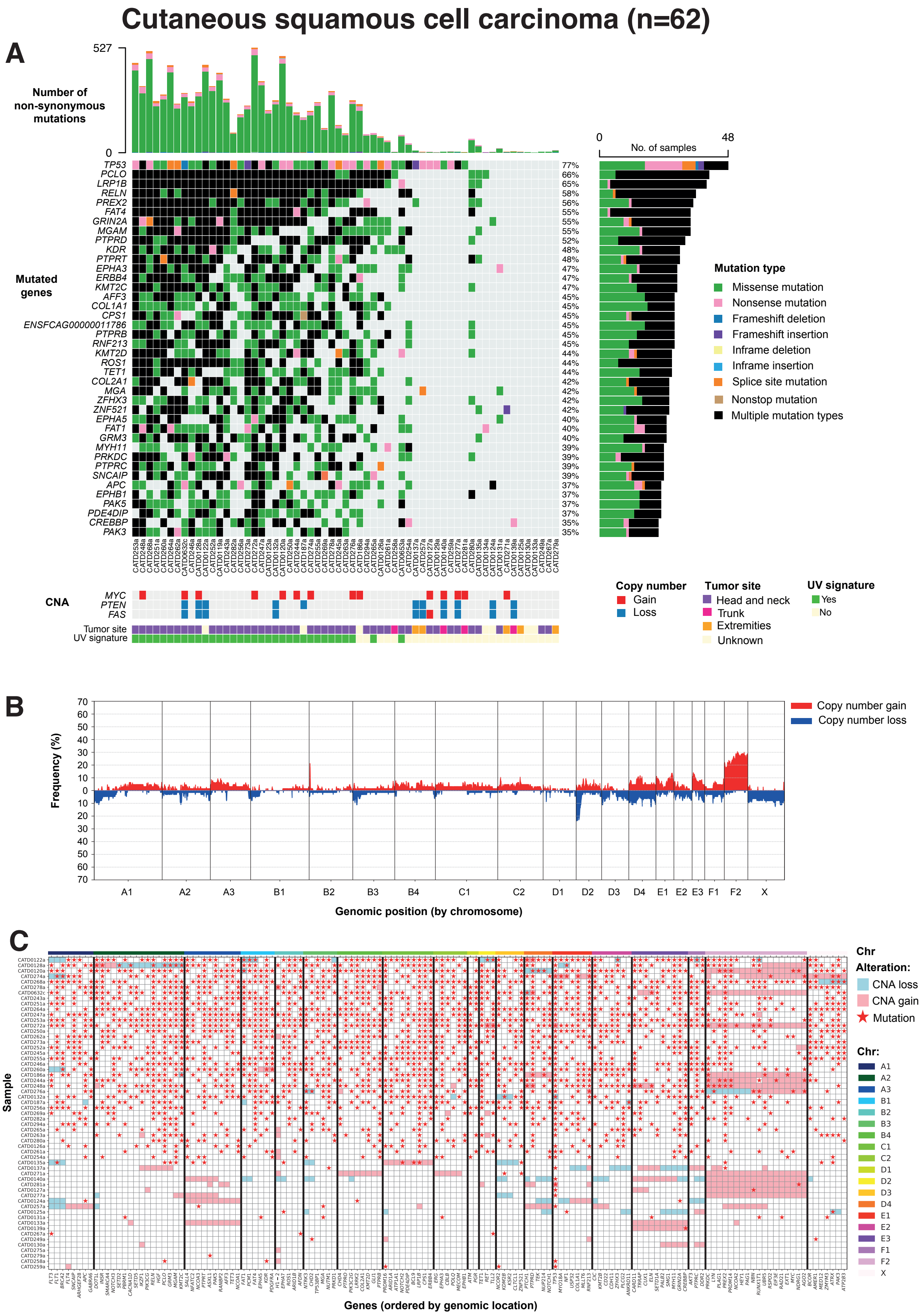
Cutaneous squamous cell carcinoma (n=62)
A
Number of non-synonymous mutations
527
0
No. of samples
0
48
Mutated genes
TP53 77%
PCLO 66%
LRP1B 65%
RELN 58%
PREX2 56%
FAT4 55%
GRIN2A 55%
MGAM 55%
PTPRD 52%
KDR 48%
PTPRT 48%
EPHA3 47%
ERBB4 47%
KMT2C 47%
AFF3 45%
COL1A1 45%
CPS1 45%
ENSFCAG00000011786 45%
PTPRB 45%
RNF213 45%
KMT2D 44%
ROS1 44%
TET1 44%
COL2A1 42%
MGA 42%
ZFHX3 42%
ZNF521 42%
EPHA5 40%
FAT1 40%
GRM3 40%
MYH11 39%
PRKDC 39%
PTPRC 39%
SNCAIP 39%
APC 37%
EPHB1 37%
PAK5 37%
PDE4DIP 37%
CREBBP 35%
PAK3 35%
Mutation type
Missense mutation
Nonsense mutation
Frameshift deletion
Frameshift insertion
Inframe deletion
Inframe insertion
Splice site mutation
Nonstop mutation
Multiple mutation types
CNA
MYC
PTEN
FAS
Tumor site
UV signature
Copy number
Gain
Loss
Tumor site
Head and neck
Trunk
Extremities
Unknown
UV signature
Yes
No
B
Frequency (%)
Copy number gain
Copy number loss
A1 A2 A3 B1 B2 B3 B4 C1 C2 D1 D2 D3 D4 E1 E2 E3 F1 F2 X
Genomic position (by chromosome)
C
Sample
Genes (ordered by genomic location)
Chr
Alteration:
CNA loss
CNA gain
Mutation
Chr:
A1 A2 A3 B1 B2 B3 B4 C1 C2 D1 D2 D3 D4 E1 E2 E3 F1 F2 X

Fig. S9. The mutational landscape of cancer-associated genes in feline cutaneous squamous cell carcinoma. (**A**) Oncoplot showing mutations present in the top 40 mutated genes (defined as recurrently mutated genes, listed in alphabetical order, followed by genes mutated in a single sample, listed in alphabetical order, if the maximum of 40 genes was not reached). The full list of protein-altering mutations identified within each sample is in Table S3. Tumors without any protein-altering mutations are not shown in the plot (but are accounted for in the frequency percentages shown). (**B**) Frequency plot showing the copy number alterations (CNA) across the genome (at a chromosome level). Copy number (CN) gains (amplifications; log2 $\geq$ +0.32) are shown in red, while CN losses (deletions; log2 $\leq$ -0.40) are shown in blue. (**C**) Oncoplot showing the CNA, overlaid with the somatic mutations. A star indicates at least one protein-altering SNV/MNV/indel in that gene. The plot is filtered to only show genes with $\geq$ 15 alterations (either mutations or copy number alterations). Tumors are included only if they have at least one alteration in this filtered gene list.

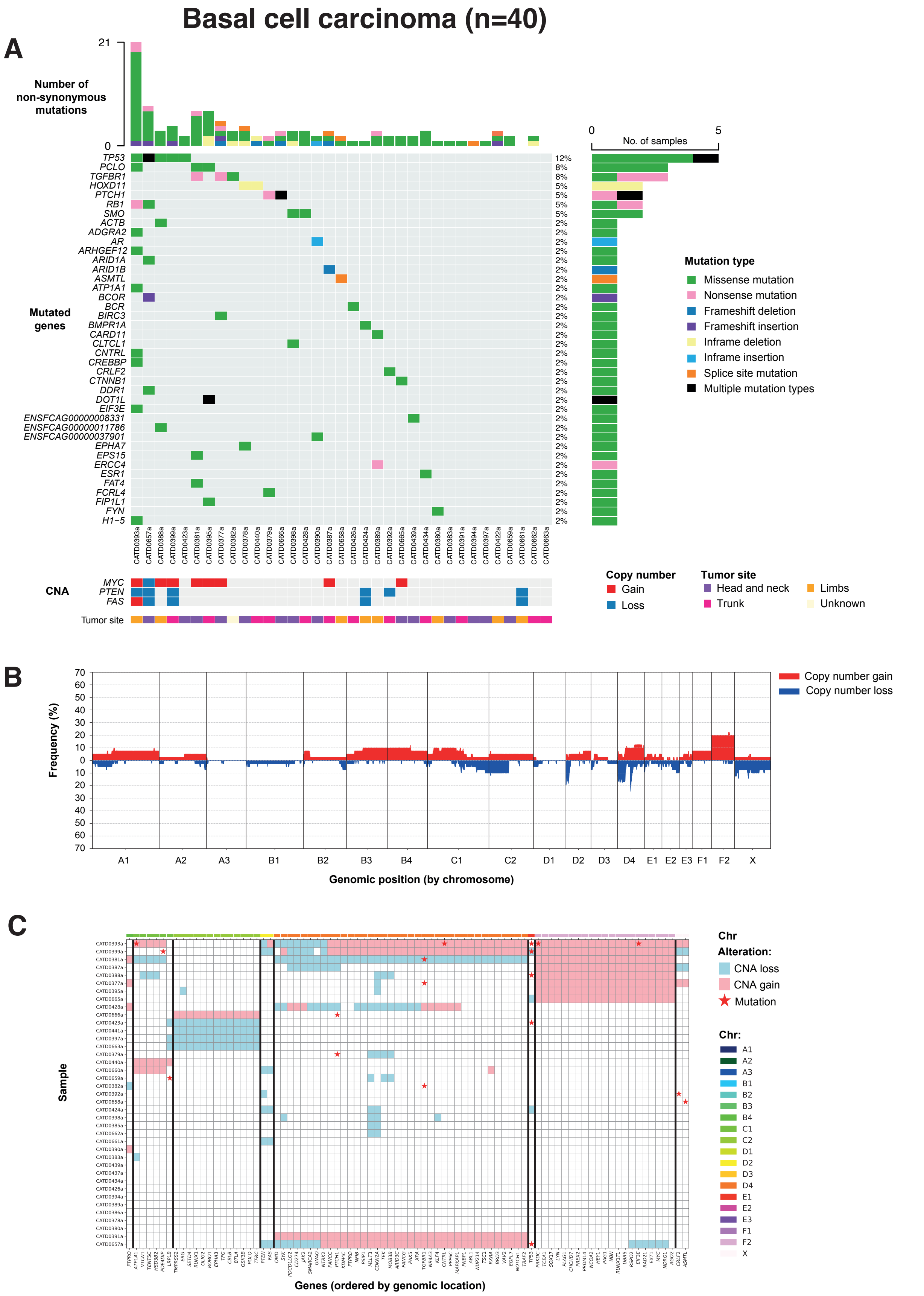
Basal cell carcinoma (n=40)
A
Number of non-synonymous mutations
21
0
No. of samples
5
TP53
PCLO
TGFBR1
HOXD11
PTCH1
RB1
SMO
ACTB
ADGRA2
AR
ARHGEF12
ARID1A
ARID1B
ASMTL
ATP1A1
BCOR
BCR
BIRC3
BMPR1A
CARD11
CLTCL1
CNTRL
CREBBP
CRLF2
CTNNB1
DDR1
DOT1L
EIF3E
ENSFCAG00000008331
ENSFCAG00000011786
ENSFCAG00000037901
EPHA7
EPS15
ERCC4
ESR1
FAT4
FCRL4
FIP1L1
FYN
H1−5
Mutated genes
12%
8%
8%
5%
5%
5%
5%
2%
Mutation type
Missense mutation
Nonsense mutation
Frameshift deletion
Frameshift insertion
Inframe deletion
Inframe insertion
Splice site mutation
Multiple mutation types
CNA
MYC
PTEN
FAS
Tumor site
Copy number
Gain
Loss
Tumor site
Head and neck
Trunk
Limbs
Unknown
B
Frequency (%)
Copy number gain
Copy number loss
A1
A2
A3
B1
B2
B3
B4
C1
C2
D1
D2
D3
D4
E1
E2
E3
F1
F2
X
Genomic position (by chromosome)
C
Sample
Genes (ordered by genomic location)
Chr
Alteration:
CNA loss
CNA gain
Mutation
Chr:
A1
A2
A3
B1
B2
B3
B4
C1
C2
D1
D2
D3
D4
E1
E2
E3
F1
F2
X

Fig. S10. The mutational landscape of cancer-associated genes in feline basal cell carcinoma. (**A**) Oncoplot showing mutations present in the top 40 mutated genes (defined as recurrently mutated genes, listed in alphabetical order, followed by genes mutated in a single sample, listed in alphabetical order, if the maximum of 40 genes was not reached). The full list of protein-altering mutations identified within each sample is in Table S3. Tumors without any protein-altering mutations are not shown in the plot (but are accounted for in the frequency percentages shown). (**B**) Frequency plot showing the copy number alterations (CNA) across the genome (at a chromosome level). Copy number (CN) gains (amplifications; log2 $\geq$ +0.32) are shown in red, while CN losses (deletions; log2 $\leq$ -0.40) are shown in blue. (**C**) Oncoplot showing the CNA landscape, overlaid with the somatic mutations. A star indicates at least one protein-altering SNV/MNV/indel in that gene. The plot is filtered to only show genes with $\geq$ 3 alterations (either mutations or copy number alterations). Tumors are included only if they have at least one alteration in this filtered gene list.

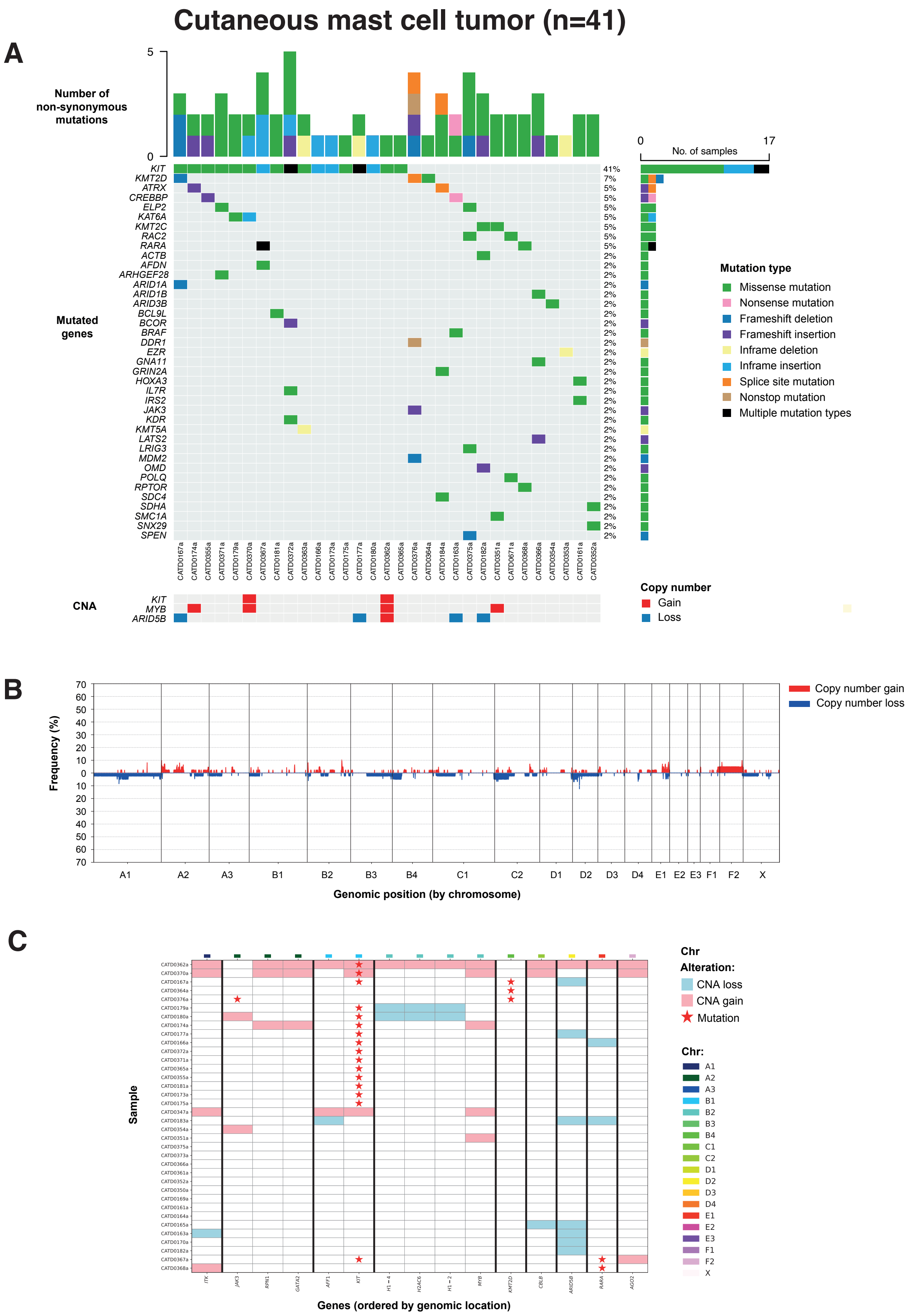
Cutaneous mast cell tumor (n=41)
A
Number of non-synonymous mutations
Mutated genes
KIT
KMT2D
ATRX
CREBBP
ELP2
KAT6A
KMT2C
RAC2
RARA
ACTB
AFDN
ARHGEF28
ARID1A
ARID1B
ARID3B
BCL9L
BCOR
BRAF
DDR1
EZR
GNA11
GRIN2A
HOXA3
IL7R
IRS2
JAK3
KDR
KMT5A
LATS2
LRIG3
MDM2
OMD
POLQ
RPTOR
SDC4
SDHA
SMC1A
SNX29
SPEN
No. of samples
Mutation type
Missense mutation
Nonsense mutation
Frameshift deletion
Frameshift insertion
Inframe deletion
Inframe insertion
Splice site mutation
Nonstop mutation
Multiple mutation types
CNA
MYB
ARID5B
Copy number
Gain
Loss
B
Frequency (%)
Copy number gain
Copy number loss
Genomic position (by chromosome)
C
Chr
Alteration:
CNA loss
CNA gain
Mutation
Chr:
Sample
Genes (ordered by genomic location)

Fig. S11. The mutational landscape of cancer-associated genes in feline cutaneous mast cell tumor. (**A**) Oncoplot showing mutations present in the top 40 mutated genes (defined as recurrently mutated genes, listed in alphabetical order, followed by genes mutated in a single sample, listed in alphabetical order, if the maximum of 40 genes was not reached). The full list of protein-altering mutations identified within each sample is in Table S3. Tumors without any protein-altering mutations are not shown in the plot (but are accounted for in the frequency percentages shown). (**B**) Frequency plot showing the copy number alterations across the genome (at a chromosome level). Copy number (CN) gains (amplifications; $\log2 \geq +0.32$) are shown in red, while CN losses (deletions; $\log2 \leq -0.40$) are shown in blue. (**C**) Oncoplot showing the CNA landscape, overlaid with the somatic mutations. A star indicates at least one protein-altering SNV/MNV/indel in that gene. The plot is filtered to only show genes with ≥ 2 alterations (either mutations or copy number alterations). Tumors are included only if they have at least one alteration in this filtered gene list.

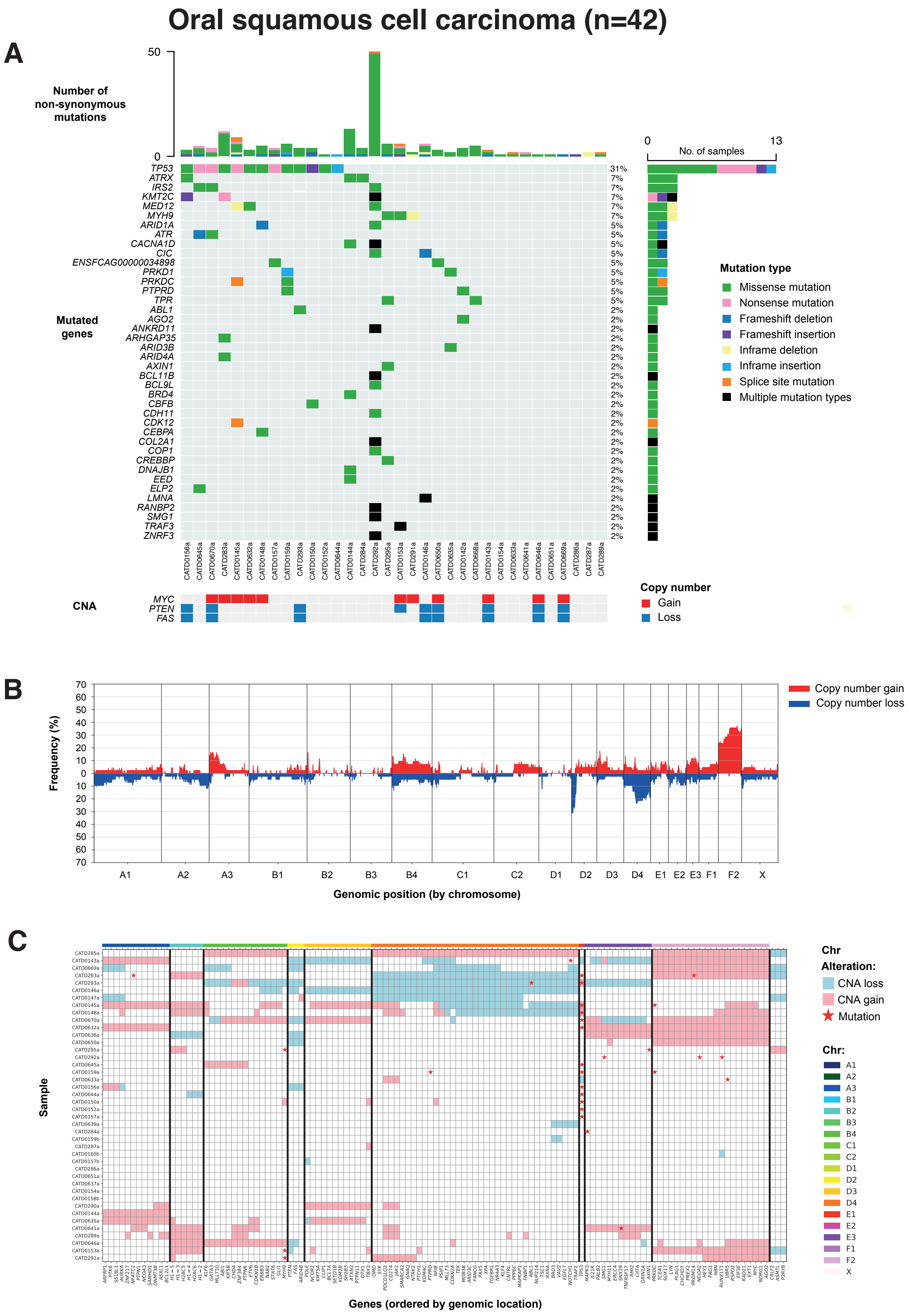
Oral squamous cell carcinoma (n=42)
A
Number of non-synonymous mutations
50
0
Mutated genes
TP53
ATRX
IRS2
KMT2C
MED12
MYH9
ARID1A
ATR
CACNA1D
CIC
ENSFCAG00000034898
PRKD1
PRKDC
PTPRD
TPR
ABL1
AGO2
ANKRD11
ARHGAP35
ARID3B
ARID4A
AXIN1
BCL11B
BCL9L
BRD4
CBFB
CDH11
CDK12
CEBPA
COL2A1
COP1
CREBBP
DNAJB1
EED
ELP2
LMNA
RANBP2
SMG1
TRAF3
ZNRF3
31%
7%
5%
2%
No. of samples
0
13
Mutation type
Missense mutation
Nonsense mutation
Frameshift deletion
Frameshift insertion
Inframe deletion
Inframe insertion
Splice site mutation
Multiple mutation types
CATD0156a
CATD0645a
CATD0670a
CATD283a
CATD0145a
CATD0632a
CATD0148a
CATD0157a
CATD0159a
CATD293a
CATD0150a
CATD0152a
CATD0644a
CATD0144a
CATD284a
CATD292a
CATD295a
CATD0153a
CATD291a
CATD0146a
CATD0650a
CATD0635a
CATD0142a
CATD0668a
CATD0143a
CATD0154a
CATD0633a
CATD0641a
CATD0646a
CATD0651a
CATD0669a
CATD286a
CATD287a
CATD289a
CNA
MYC
PTEN
FAS
Copy number
Gain
Loss
B
Frequency (%)
70
60
50
40
30
20
10
0
Copy number gain
Copy number loss
A1
A2
A3
B1
B2
B3
B4
C1
C2
D1
D2
D3
D4
E1
E2
E3
F1
F2
X
Genomic position (by chromosome)
C
Sample
Chr
Alteration:
CNA loss
CNA gain
Mutation
Chr:
Genes (ordered by genomic location)

Fig. S12. The mutational landscape of cancer-associated genes in feline oral squamous cell carcinoma. (**A**) Oncoplot showing mutations present in the top 40 mutated genes (defined as recurrently mutated genes, listed in alphabetical order, followed by genes mutated in a single sample, listed in alphabetical order, if the maximum of 40 genes was not reached). The full list of protein-altering mutations identified within each sample is in Table S3. Tumors without any protein-altering mutations are not shown in the plot (but are accounted for in the frequency percentages shown). (**B**) Frequency plot showing the copy number alterations (CNA) across the genome (at a chromosome level). Copy number (CN) gains (amplifications; log2 $\geq$ +0.32) are shown in red, while CN losses (deletions; log2 $\leq$ -0.40) are shown in blue. (**C**) Oncoplot showing the CNA landscape, overlaid with the somatic mutations. A star indicates at least one protein-altering SNV/MNV/indel in that gene. The plot is filtered to only show genes with $\geq$ 4 alterations (either mutations or copy number alterations). Tumors are included only if they have at least one alteration in this filtered gene list.

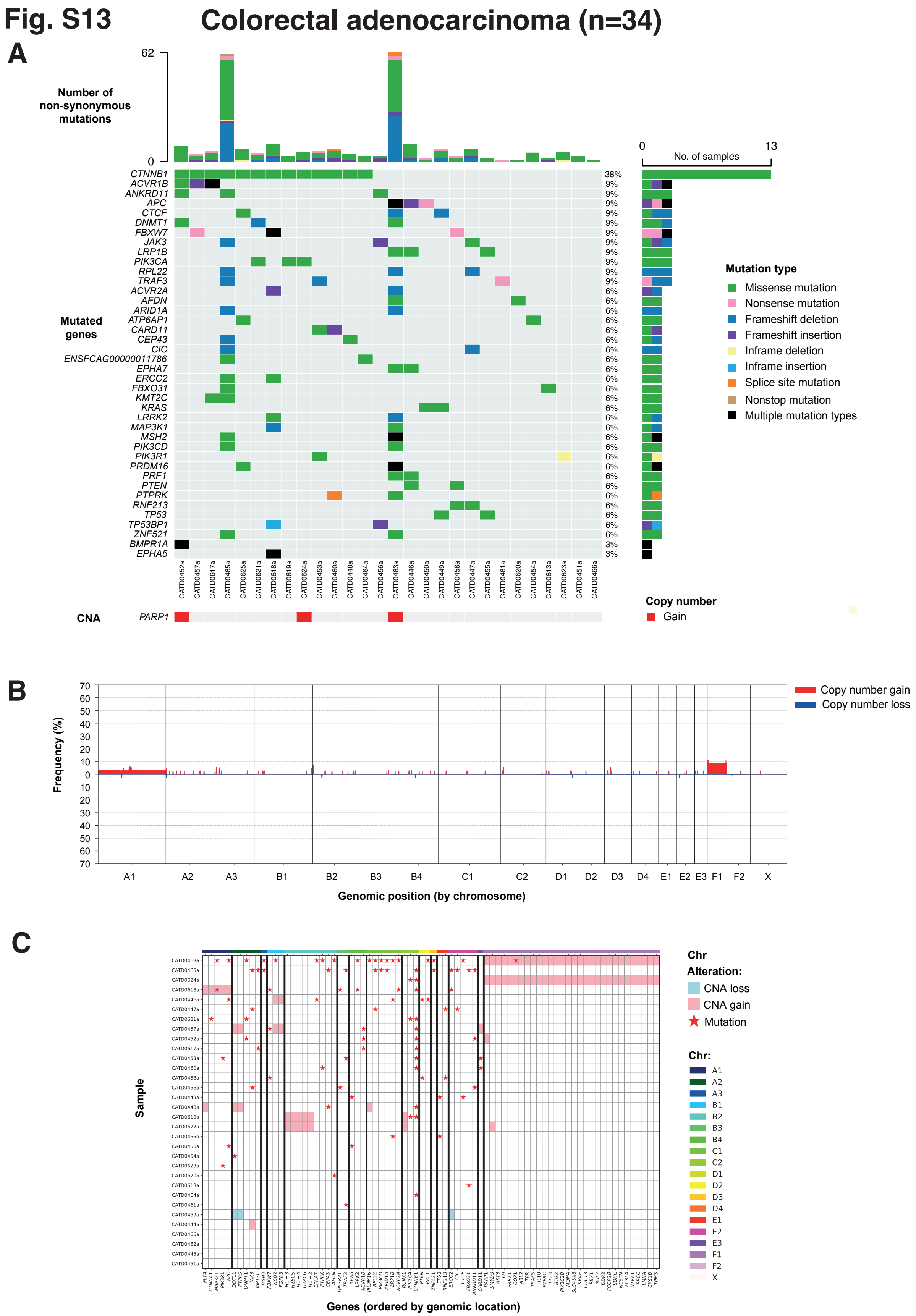
Fig. S13
Colorectal adenocarcinoma (n=34)
A
Number of non-synonymous mutations
Mutated genes
No. of samples
Mutation type
Missense mutation
Nonsense mutation
Frameshift deletion
Frameshift insertion
Inframe deletion
Inframe insertion
Splice site mutation
Nonstop mutation
Multiple mutation types
CNA
PARP1
Copy number
Gain
B
Frequency (%)
Copy number gain
Copy number loss
Genomic position (by chromosome)
C
Sample
Chr
Alteration:
CNA loss
CNA gain
Mutation
Genes (ordered by genomic location)

Fig. S13. The mutational landscape of cancer-associated genes in feline colorectal adenocarcinoma. (**A**) Oncoplot showing mutations present in the top 40 mutated genes (defined as recurrently mutated genes, listed in alphabetical order, followed by genes mutated in a single sample, listed in alphabetical order, if the maximum of 40 genes was not reached). The full list of protein-altering mutations identified within each sample is in Table S3. Tumors without any protein-altering mutations are not shown in the plot (but are accounted for in the frequency percentages shown). (**B**) Frequency plot showing the copy number alterations (CNA) across the genome (at a chromosome level). Copy number (CN) gains (amplifications; log2 $\geq$ +0.32) are shown in red, while CN losses (deletions; log2 $\leq$ -0.40) are shown in blue. (**C**) Oncoplot showing the CNA landscape, overlaid with the somatic mutations. A star indicates at least one protein-altering SNV/MNV/indel in that gene. The plot is filtered to only show genes with $\geq$ 2 alterations (either mutations or copy number alterations). Tumors are included only if they have at least one alteration in this filtered gene list.

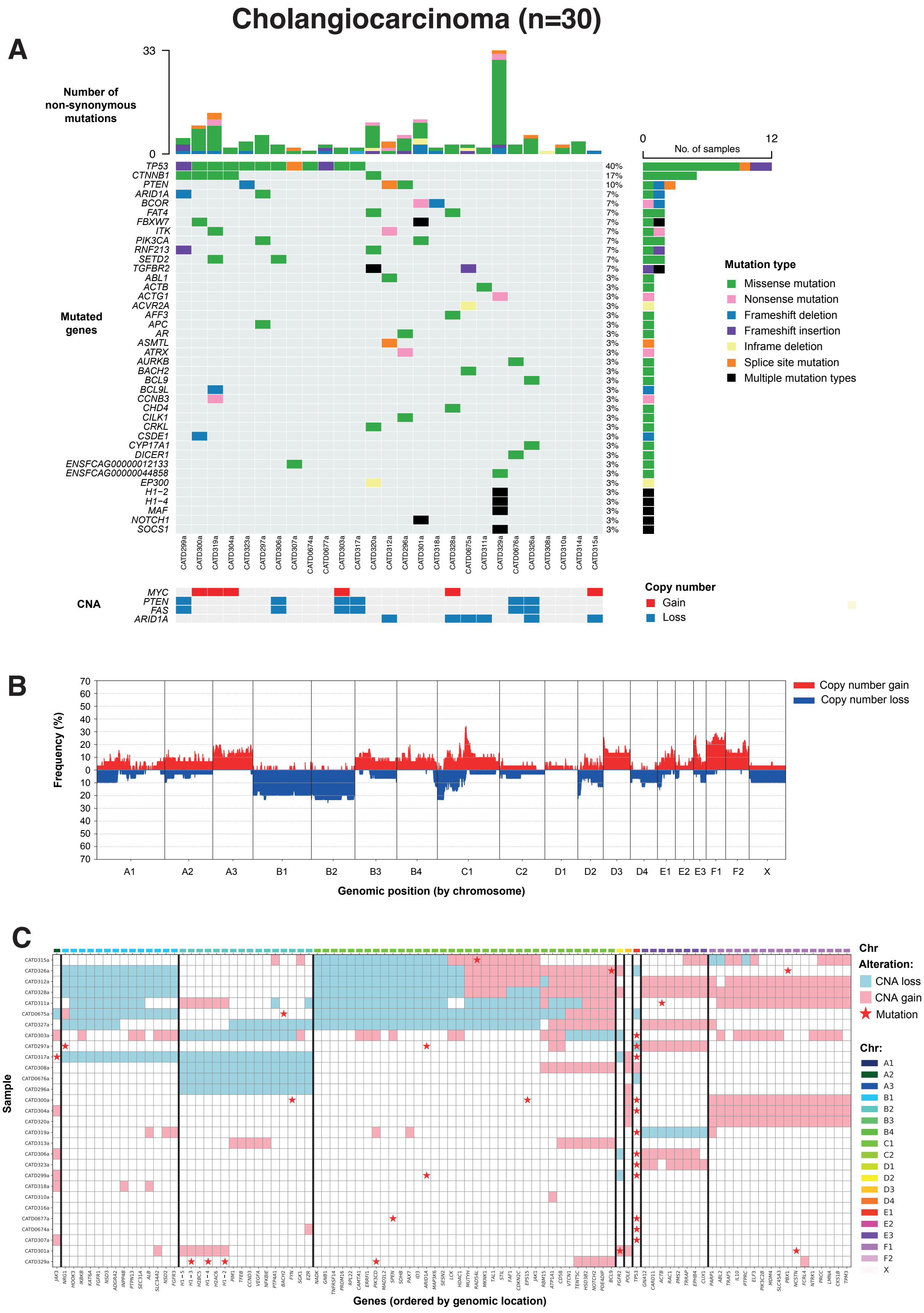
Cholangiocarcinoma (n=30)
A
Number of non-synonymous mutations
Mutated genes
No. of samples
Mutation type
Missense mutation
Nonsense mutation
Frameshift deletion
Frameshift insertion
Inframe deletion
Splice site mutation
Multiple mutation types
CNA
Copy number
Gain
Loss
B
Frequency (%)
Copy number gain
Copy number loss
Genomic position (by chromosome)
C
Sample
Genes (ordered by genomic location)
Chr
Alteration:
CNA loss
CNA gain
Mutation
Chr:

Fig. S14. The mutational landscape of cancer-associated genes in feline cholangiocarcinoma. (**A**) Oncoplot showing mutations present in the top 40 mutated genes (defined as recurrently mutated genes, listed in alphabetical order, followed by genes mutated in a single sample, listed in alphabetical order, if the maximum of 40 genes was not reached). The full list of protein-altering mutations identified within each sample is in Table S3. Tumors without any protein-altering mutations are not shown in the plot (but are accounted for in the frequency percentages shown). (**B**) Frequency plot showing the copy number alterations (CNA) across the genome (at a chromosome level). Copy number (CN) gains (amplifications; log2 $\geq$ +0.32) are shown in red, while CN losses (deletions; log2 $\leq$ -0.40) are shown in blue. (**C**) Oncoplot showing the CNA landscape, overlaid with the somatic mutations. A star indicates at least one protein-altering SNV/MNV/indel in that gene. The plot is filtered to only show genes with $\geq$ 5 alterations (either mutations or copy number alterations). Tumors are included only if they have at least one alteration in this filtered gene list.

Pancreatic adenocarcinoma (n=29)

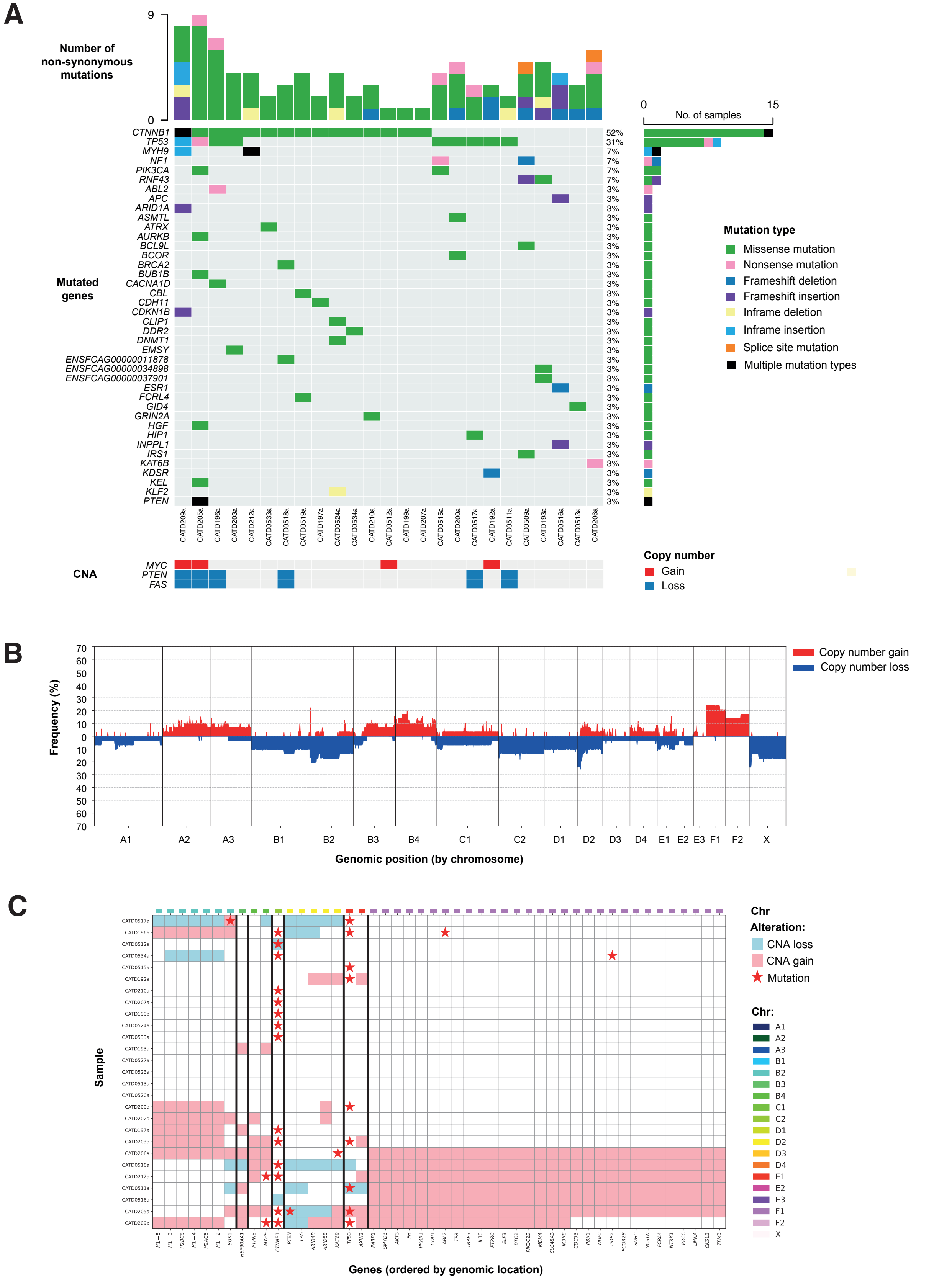

Fig. S15. The mutational landscape of cancer-associated genes in feline pancreatic adenocarcinoma. (**A**) Oncoplot showing mutations present in the top 40 mutated genes (defined as recurrently mutated genes, listed in alphabetical order, followed by genes mutated in a single sample, listed in alphabetical order, if the maximum of 40 genes was not reached). The full list of protein-altering mutations identified within each sample is in Table S3. Tumors without any protein-altering mutations are not shown in the plot (but are accounted for in the frequency percentages shown). (**B**) Frequency plot showing the copy number alterations (CNA) across the genome (at a chromosome level). Copy number (CN) gains (amplifications; log2 $\geq +0.32$) are shown in red, while CN losses (deletions; log2 ≤ -0.40) are shown in blue. (**C**) Oncoplot showing the CNA landscape, overlaid with the somatic mutations. A star indicates at least one protein-altering SNV/MNV/indel in that gene. The plot is filtered to only show genes with ≥ 3 alterations (either mutations or copy number alterations). Tumors are included only if they have at least one alteration in this filtered gene list.

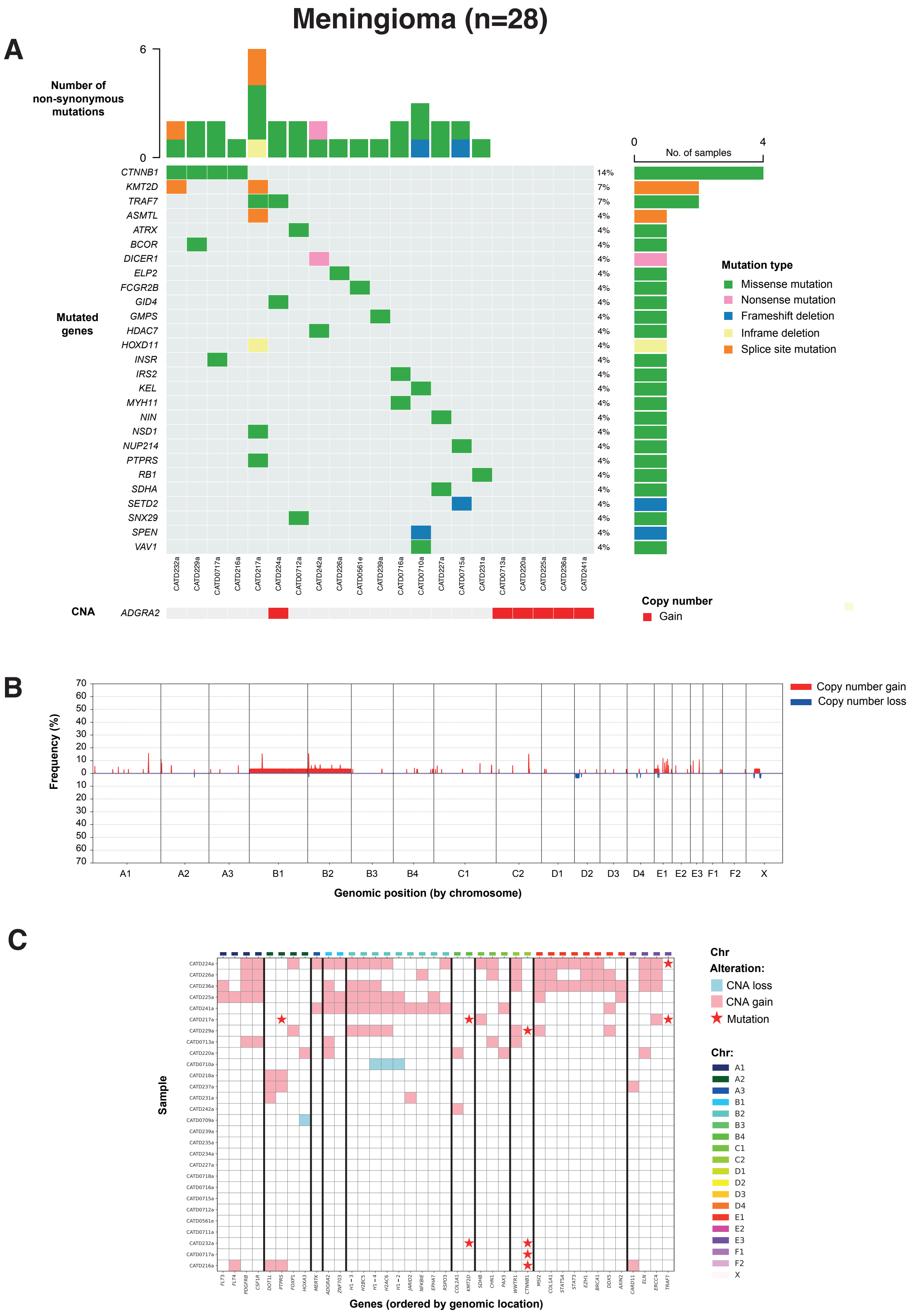

Meningioma (n=28)
A
Number of non-synonymous mutations
Mutated genes
No. of samples
Mutation type
Missense mutation
Nonsense mutation
Frameshift deletion
Inframe deletion
Splice site mutation
CTNNB1
KMT2D
TRAF7
ASMTL
ATRX
BCOR
DICER1
ELP2
FCGR2B
GID4
GMPS
HDAC7
HOXD11
INSR
IRS2
KEL
MYH11
NIN
NSD1
NUP214
PTPRS
RB1
SDHA
SETD2
SNX29
SPEN
VAV1
CNA
ADGRA2
Copy number
Gain
B
Frequency (%)
Copy number gain
Copy number loss
Genomic position (by chromosome)
C
Sample
Genes (ordered by genomic location)
Chr
Alteration:
CNA loss
CNA gain
Mutation

Fig. S16. The mutational landscape of cancer-associated genes in feline meningioma. (**A**) Oncoplot showing mutations present in the top 40 mutated genes (defined as recurrently mutated genes, listed in alphabetical order, followed by genes mutated in a single sample, listed in alphabetical order, if the maximum of 40 genes was not reached). The full list of protein-altering mutations identified within each sample is in Table S3. Tumors without any protein-altering mutations are not shown in the plot (but are accounted for in the frequency percentages shown). (**B**) Frequency plot showing the copy number alterations (CNA) across the genome (at a chromosome level). Copy number (CN) gains (amplifications; $\log 2 \geq +0.32$) are shown in red, while CN losses (deletions; $\log 2 \leq -0.40$) are shown in blue. (**C**) Oncoplot showing the CNA landscape, overlaid with the somatic mutations. A star indicates at least one protein-altering SNV/MNV/indel in that gene. The plot is filtered to only show genes with ≥ 2 alterations (either mutations or copy number alterations). Tumors are included only if they have at least one alteration in this filtered gene list.

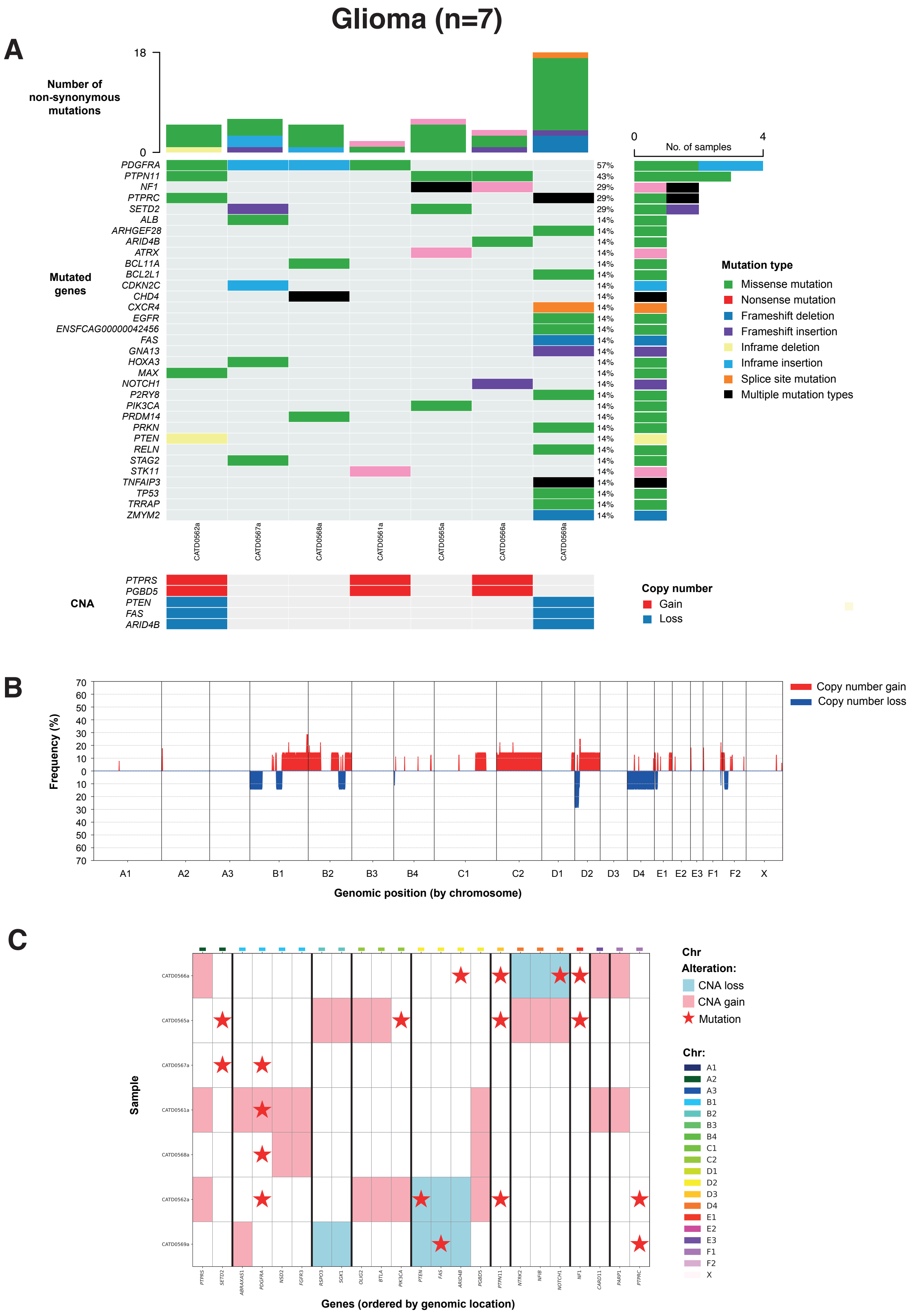

Glioma (n=7)
A
Number of non-synonymous mutations
18
0
Mutated genes
PDGFRA
PTPN11
NF1
PTPRC
SETD2
ALB
ARHGEF28
ARID4B
ATRX
BCL11A
BCL2L1
CDKN2C
CHD4
CXCR4
EGFR
ENSFCAG00000042456
FAS
GNA13
HOXA3
MAX
NOTCH1
P2RY8
PIK3CA
PRDM14
PRKN
PTEN
RELN
STAG2
STK11
TNFAIP3
TP53
TRRAP
ZMYM2
57%
43%
29%
29%
29%
14%
CATD0562a
CATD0567a
CATD0568a
CATD0561a
CATD0565a
CATD0566a
CATD0569a
No. of samples
Mutation type
Missense mutation
Nonsense mutation
Frameshift deletion
Frameshift insertion
Inframe deletion
Inframe insertion
Splice site mutation
Multiple mutation types
CNA
PTPRS
PGBD5
PTEN
FAS
ARID4B
Copy number
Gain
Loss
B
Frequency (%)
Copy number gain
Copy number loss
A1 A2 A3 B1 B2 B3 B4 C1 C2 D1 D2 D3 D4 E1 E2 E3 F1 F2 X
Genomic position (by chromosome)
C
Sample
Genes (ordered by genomic location)
Chr
Alteration:
CNA loss
CNA gain
Mutation
Chr:

Fig. S17. The mutational landscape of cancer-associated genes in feline glioma. (**A**) Oncoplot showing mutations present in the top 40 mutated genes (defined as recurrently mutated genes, listed in alphabetical order, followed by genes mutated in a single sample, listed in alphabetical order, if the maximum of 40 genes was not reached). The full list of protein-altering mutations identified within each sample is in Table S3. Tumors without any protein-altering mutations are not shown in the plot (but are accounted for in the frequency percentages shown). (**B**) Frequency plot showing the copy number alterations (CNA) across the genome (at a chromosome level). Copy number (CN) gains (amplifications; log2 $\geq$ +0.32) are shown in red, while CN losses (deletions; log2 $\leq$ -0.40) are shown in blue. (**C**) Oncoplot showing the CNA landscape, overlaid with the somatic mutations. A star indicates at least one protein-altering SNV/MNV/indel in that gene. The plot is filtered to only show genes with $\geq$ 2 alterations (either mutations or copy number alterations). Tumors are included only if they have at least one alteration in this filtered gene list.

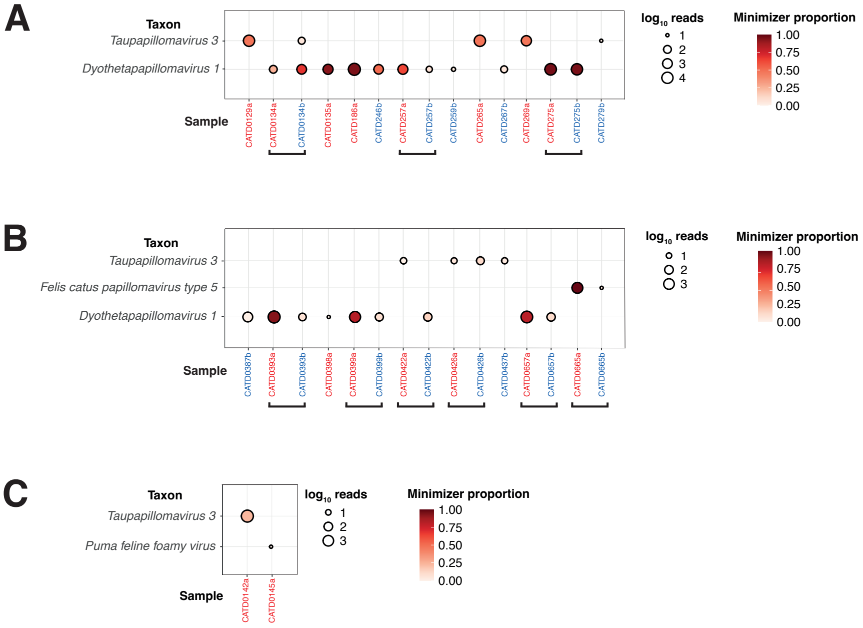
A
Taxon
Taupapillomavirus 3
Dyothetapapillomavirus 1
Sample
CATD0129a
CATD0134a
CATD0134b
CATD0135a
CATD186a
CATD246b
CATD257a
CATD257b
CATD259b
CATD265a
CATD267b
CATD269a
CATD275a
CATD275b
CATD279b
log10 reads
1
2
3
4
Minimizer proportion
1.00
0.75
0.50
0.25
0.00
B
Taxon
Taupapillomavirus 3
Felis catus papillomavirus type 5
Dyothetapapillomavirus 1
Sample
CATD0387b
CATD0393a
CATD0393b
CATD0398a
CATD0399a
CATD0399b
CATD0422a
CATD0422b
CATD0426a
CATD0426b
CATD0437b
CATD0657a
CATD0657b
CATD0665a
CATD0665b
log10 reads
1
2
3
Minimizer proportion
1.00
0.75
0.50
0.25
0.00
C
Taxon
Taupapillomavirus 3
Puma feline foamy virus
Sample
CATD0142a
CATD0145a
log10 reads
1
2
3
Minimizer proportion
1.00
0.75
0.50
0.25
0.00

Fig. S18. Presence of viral DNA in feline tumor and matched normal tissue. (**A**) Tissue samples from cats with cutaneous squamous cell carcinoma. (**B**) Tissue samples from cats with basal cell carcinoma. (**C**) Tissue samples from cats with oral squamous cell carcinoma. The suffix 'a' indicates a tumor sample and the suffix 'b' indicates a normal sample. Log_{10} reads represent the number of clade-level reads observed. Minimizer proportion represents the number of clade-level minimizers observed in the sample relative to the total clade-level minimizers present in the Kraken database. Only taxa with significant minimizer discoveries (BH adjusted *P-value* < 0.05) and more than five distinct minimizers observed across the cohort are shown. Tumor samples are shown in red and normal samples are shown in blue, with tumor/normal samples from the same cat indicated by a bracket.

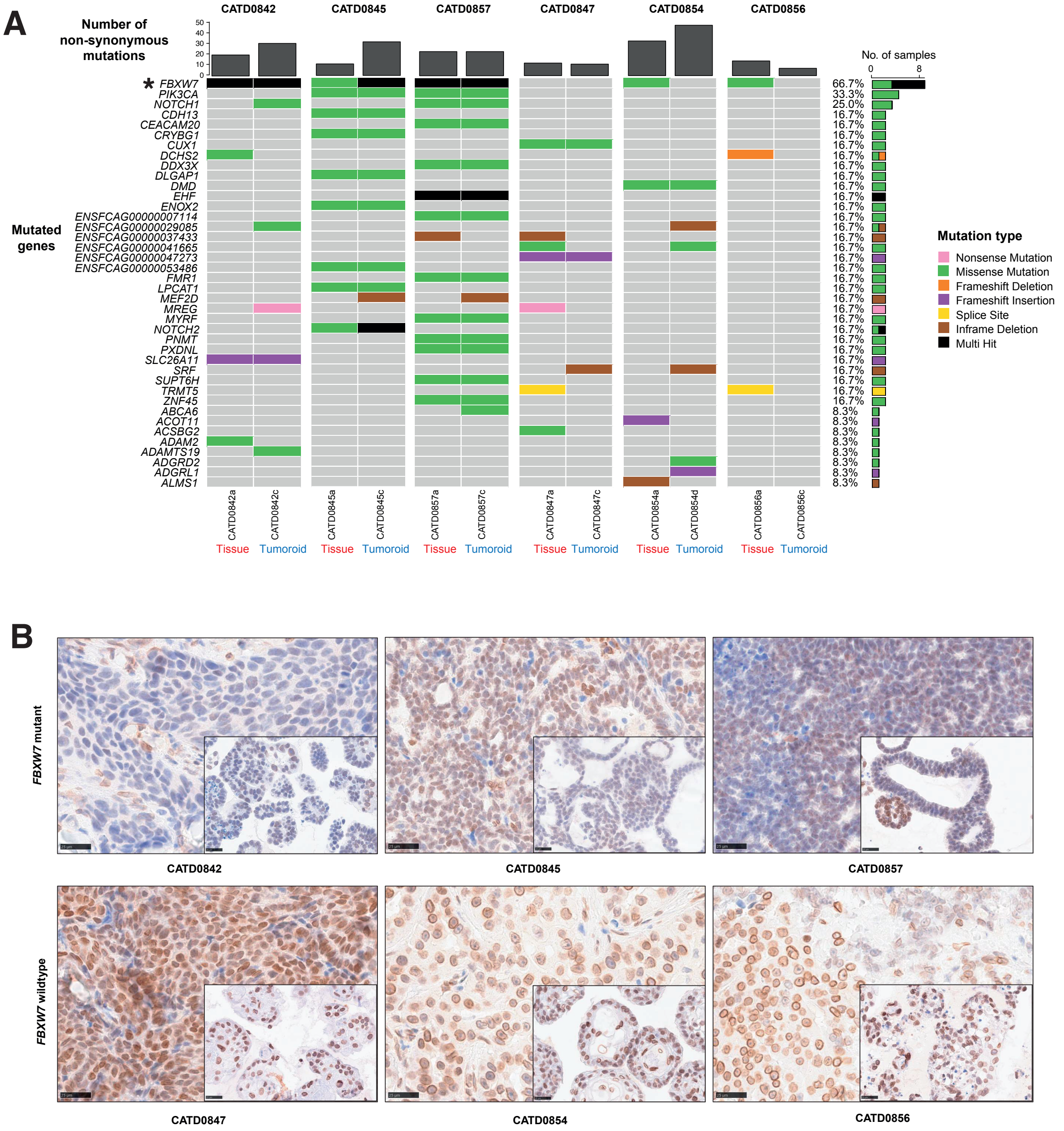
A
B
Number of non-synonymous mutations
CATD0842
CATD0845
CATD0857
CATD0847
CATD0854
CATD0856
No. of samples
Mutated genes
FBXW7
PIK3CA
NOTCH1
CDH13
CEACAM20
CRYBG1
CUX1
DCHS2
DDX3X
DLGAP1
DMD
EHF
ENOX2
ENSFCAG00000007114
ENSFCAG00000029085
ENSFCAG00000037433
ENSFCAG00000041665
ENSFCAG00000047273
ENSFCAG00000053486
FMR1
LPCAT1
MEF2D
MREG
MYRF
NOTCH2
PNMT
PXDNL
SLC26A11
SRF
SUPT6H
TRMT5
ZNF45
ABCA6
ACOT11
ACSBG2
ADAM2
ADAMTS19
ADGRD2
ADGRL1
ALMS1
66.7%
33.3%
25.0%
16.7%
8.3%
CATD0842a
CATD0842c
CATD0845a
CATD0845c
CATD0857a
CATD0857c
CATD0847a
CATD0847c
CATD0854a
CATD0854d
CATD0856a
CATD0856c
Tissue
Tumoroid
Mutation type
Nonsense Mutation
Missense Mutation
Frameshift Deletion
Frameshift Insertion
Splice Site
Inframe Deletion
Multi Hit
FBXW7 mutant
FBXW7 wildtype

Fig. S19. ***FBXW7*** **mutational status in feline mammary carcinoma tumoroids**. (**A**) Oncoplot showing only the top 40 mutated genes identified in the feline mammary carcinoma tumoroids and the tissue samples from which they were derived. All non-synonymous and splice site mutations identified within each sample are listed in Table S3. The asterisk is highlighting their *FBXW7* status. (**B**) Immunohistochemical staining for FBXW7 primary tumor tissue (larger left panels) and their corresponding tumoroids (smaller right panels), with their *FBXW7* status on the vertical axis. Note: A clear differential expression of FBXW7 is observed, with strong staining in wildtype samples and reduced signal in mutant ones. In cases CADT0854 and CTD0856, intratumoral heterogeneity is evident in the primary tissue, showing a distinct transition from high to low FBXW7-expressing cells. Notably, tumoroids from these tumors retained a wildtype FBXW7 status, despite mutations detected in the corresponding primary tissue by sequencing, suggesting clonal selection during tumoroid derivation. Scale bar = 25 μm.

Legends for Supplementary Tables S1-S18

Table S1. A summary of the signalment data on a per-cohort basis. Sex abbreviations: F, female; M, male; U, unknown. Breed abbreviations: BSH, British Shorthair; DLH, Domestic Longhair; DMH, Domestic Mediumhair; DSH, Domestic Shorthair; MC, Maine Coon; Siam, Siamese; U, Unknown. The breed was supplied by the owner. Subtype abbreviations: ERneg, estrogen receptor negative; ERpos, estrogen receptor positive; ERpos_low, estrogen receptor low positive; ERunk, estrogen receptor status unknown; NK, natural killer.

Table S2. Details of the samples used in the study. Sample information and associated signalment data, separated by tumor type. T/N indicates whether the sample was tumor (T) or normal (N) tissue. Patient ID indicates which tumor and normal samples are from the same cat. Sex is male (M), female (F), or unknown (U). Age is age at diagnosis in years or unknown (U). Breed abbreviations: British Shorthair (BSH), Domestic Longhair (DLH), Domestic Mediumhair (DMH), Domestic Shorthair (DSH), Maine Coon (MC), Siamese (Siam), Unknown (U). The breed was supplied by the owner. The country is the location of the Veterinary Pathology Laboratory from where the tissue sample was obtained. Tumor type abbreviations (used on the tabs): BCC, basal cell carcinoma; CCA, cholangiocarcinoma; CRC, colorectal adenocarcinoma; GLIO, glioma; LUCA, lung adenocarcinoma; LYM, lymphoma; MAM, mammary carcinoma; cMCT, cutaneous mast cell tumor; MEN, meningioma; OSA, osteosarcoma; PANC, pancreatic adenocarcinoma; cSCC, cutaneous squamous cell carcinoma; oSCC, oral squamous cell carcinoma; MAM_WES, whole-exome sequencing of mammary carcinoma (and tumoroids).

Table S3. Somatic non-synonymous and splice site mutations identified in each of the tumors. Each of the different tumor types are in individual tabs (either mapped to the FelCat9 or Fca126 reference genomes). Tumor type abbreviations (used on each tab): BCC, basal cell carcinoma; CCA, cholangiocarcinoma; CRC, colorectal adenocarcinoma; GLIO, glioma; LUCA, lung adenocarcinoma; LYM, lymphoma (B-cell and T-cell; the NK-cell case, CATD0596a, is present in both cohorts); MAM, mammary carcinoma; cMCT, cutaneous mast cell tumor; MEN, meningioma; OSA, osteosarcoma; PANC, pancreatic adenocarcinoma; cSCC, cutaneous squamous cell carcinoma; oSCC, oral squamous cell carcinoma; MAM_WES, mammary carcinoma (whole-exome sequencing cohort). Mutations include SNV, MNVs and short indels. The Table columns are derived from the Mutation Annotation Format (MAF) v1.0.0 and Variant Call Format (VCF) v4.1, with additional information from Variant Effect Predictor (VEP) annotations. For feline genes not assigned a gene symbol, only the Ensembl gene ID is listed. POS_VCF and FILTER are the position and variant filter from the original VCF file. VAF_tum and VAF_norm are the variant allele frequencies in the tumor and normal sample, respectively. The ref_count, alt_count and depth columns indicate the number of supporting reference alleles, alternate alleles and total depth. Consequence is the predicted variant consequence using VEP terminology and Main_consequence_VEP shows the single most deleterious consequence, if there are multiple consequences. REF_VEP and ALT_REF are the reference and alternative alleles, represented in VEP format. Tumor_ID/Normal_ID indicate the IDs of the matched tumor/normal samples. dbSNP indicates whether the variant was found in the single nucleotide polymorphism database (dbSNP). Canonical indicates whether the annotated consequence corresponds to the canonical transcript, as defined in Ensembl v104. The SIFT column provides the SIFT score and

category. The 99LivesV9 column indicates whether the variant was found in the updated 99 Lives Cat Genome Consortium database (418 cats; originally based on the Fca126 assembly and lifted over to Felis_catus_9.0 with Picard LiftoverVcf) and 99LivesV9_AF gives the variant allele frequency within the cohort. The Added_By_MAF_Updater column is TRUE/FALSE for whether the variant was added to the MAF by MAF_Updater as explained in the materials and methods).

Table S4. Tumor samples in the cutaneous squamous cell carcinoma cohort showing the presence of COSMIC Signature SBS7. Samples included in this Table passed the significance cut off values (exposure value ≥0.1 and reconstructed cosine value of ≥ 0.850). The reconstructed cosine value shown is from the single sample re-fitting. For samples where only one signature was found with an exposure level of ≥0.1 (CATD187a, CATD269a, CAT276a), re-fitting analysis was unable to be performed, so the values shown are the estimated exposure values from the original fitting (which assess the relative exposure value of all 96 signatures per sample); no re-fitting reconstructed cosine value was then included in the Table, marked as "NA" (although the fitting reconstructed cosine value was ≥0.850). SBS2 is attributed to activity of the AID/APOBEC family of cytidine deaminases, however it is usually found in the same samples as SBS13. Given we did not find SBS13 in our samples, it is likely the identification of SBS2 in a few samples is a false positive.

Table S5. Driver gene analysis results. Table S5 tab is the summary of the driver genes per tumor type (global q value >0.1). All other tabs are on a per tumor type basis and are the raw output from dNdSCv (containing both significant and non-significant results). Abbreviations: cv, value estimated using the covariates matrix for that tumor type; ind, indel; n, number; non, non-synonymous; p, p value; q, q value; spl, splice; syn, synonymous; w, effect size. Tumor type abbreviations (used on the tabs): BCC, basal cell carcinoma; CCA, cholangiocarcinoma; CRC, colorectal adenocarcinoma; cSCC, cutaneous squamous cell carcinoma; oSCC, oral squamous cell carcinoma; GLIO, glioma; LUCA, lung adenocarcinoma; LYM, lymphoma (B- and T-cell); MAM, mammary carcinoma; cMCT, cutaneous mast cell tumor; MEN, meningioma; OSA, osteosarcoma; PANC, pancreatic adenocarcinoma; MAM_WES, mammary carcinoma (whole-exome sequencing cohort).

Table S6. Hotspot mutations identified in the study. Hotspot mutations were defined as recurrent mutations found in ≥ 3 tumors. The cSCC cohort was excluded from this analysis due to the high mutational burden caused by DNA damage from exposure to UV light. Tumor type abbreviations: BCC, basal cell carcinoma; CCA, cholangiocarcinoma; CRC, colorectal adenocarcinoma; GLIO, glioma; LUCA, lung adenocarcinoma; LYM, lymphoma (B-cell); MAM, mammary carcinoma; cMCT, cutaneous mast cell tumor; MEN, meningioma; OSA, osteosarcoma; PANC, pancreatic adenocarcinoma; oSCC, oral squamous cell carcinoma.

Table S7. Whole chromosome copy number alterations. (A) Whole chromosome gains or losses shared across tumor types. **(B)** Whole chromosome gains or losses by tumor type. Note: the single NK cell case was included in the T-LYM cohort. Whole chromosome event = classify segments with 'Gain' if $\log2 \geq +0.32$ or 'Loss' if $\log2 \leq -0.40$ across 90% of the chromosome. Abbreviations: CNA, copy number alterations. Tumor type abbreviations: BCC, basal cell carcinoma; CCA, cholangiocarcinoma; CRC, colorectal adenocarcinoma; GLIO, glioma; LUCA, lung adenocarcinoma; LYM, lymphoma (T-cell or B-cell); MAM, mammary carcinoma; cMCT,

cutaneous mast cell tumor; MEN, meningioma; OSA, osteosarcoma; PANC, pancreatic adenocarcinoma; cSCC, cutaneous squamous cell carcinoma; oSCC, oral squamous cell carcinoma.

Table S8. Recurrent mutation and copy number alteration events on a per tumor basis. For 'recurrent mutations' only mutations n≥2 were included. For the hypermutated samples from the cutaneous squamous cell carcinoma cohort, only truncating mutations and missense mutations at recurrent hotspots were included. For 'copy number alterations', only copy number gains and losses ≤10Mb were included.

Table S9. Details of the number of samples showing a somatic mutation in a gene or copy number alteration involving the gene. Samples were only included in the Table if they had either a mutation or a copy number alteration. Each of the tumor types are in their own tab. Abbreviation: CNA, copy number alteration; Mut, mutation. Tumor type abbreviations (used on the tabs): BCC, basal cell carcinoma; CCA, cholangiocarcinoma; CRC, colorectal adenocarcinoma; GLIO, glioma; LUCA, lung adenocarcinoma; LYM_Bcell, lymphoma (B-cell subtype + one case of NK-cell subtype); LYM_Tcell, lymphoma (T-cell subtype + one case of NK-cell subtype); MAM, mammary carcinoma; cMCT, cutaneous mast cell tumor; MEN, meningioma; OSA, osteosarcoma; PANC, pancreatic adenocarcinoma; cSCC, cutaneous squamous cell carcinoma; oSCC, oral squamous cell carcinoma; MAM_WES, whole-exome sequencing of mammary carcinoma.

Table S10. Putative pathogenic germline variants. **(A)** Nonsense variants, frameshift variants and splice site insertions/deletions identified in the normal samples of each cat. The Table columns are derived from the Mutation Annotation Format (MAF) v1.0.0 and Variant Call Format (VCF) v4.1, with additional information from Variant Effect Predictor (VEP) annotations. 'Consequence' is the predicted variant effect from VEP. 'ENSP' is the Ensembl protein ID for the gene and 'Domains' are the protein domains in which the variant is located. For full MAF and VEP column definitions, see: https://docs.gdc.cancer.gov/Data/File_Formats/MAF_Format/ and https://www.ensembl.org/info/docs/tools/vep/vep_formats.html, respectively. 'Age' is in years and 'breed' abbreviations are: Domestic Longhair (DLH) and Domestic Shorthair (DSH). The breed was supplied by the owner. **(B)** Feline germline variants with orthologous human germline variants present in ClinVar. Human ClinVar variants were lifted over from GRCh38 to Felis_catus_9 and then intersected with the feline germline variants. The Table columns are derived from the Mutation Annotation Format (MAF) v1.0.0 and Variant Call Format (VCF) v4.1, with additional information from Variant Effect Predictor (VEP) annotations. 'Consequence' is the predicted variant effect from VEP, 'ENSP' is the Ensembl protein ID for the gene and 'Domains' are the protein domains in which the variant is located. For full MAF and VEP column definitions, see: https://docs.gdc.cancer.gov/Data/File_Formats/MAF_Format/ and https://www.ensembl.org/info/docs/tools/vep/vep_formats.html, respectively.

Table S11. Details of the human datasets used for cross-species comparison.

Table S12. Feline driver genes orthologs present in human actionability databases. (**A**) Driver genes present in the Target Central Resource Database (TCRD), and their Pharos target development level (TDL) rankings. (**B**) Driver genes present in the database of genes with druggable synthetic lethal (SL) partners. (**C**) Driver genes present in the OncoKB database, and

their therapeutic biomarker level. See materials and methods for details of each human actionability database.

Table S13. Feline mammary carcinoma tumoroid vinca alkaloid dose–response data. (**A**) Vincristine. (**B**) Vinorelbine.

Table S14. Bait set genes. (A) Initial list of human cancer-associated genes used as a basis for designing the feline cancer-associated bait set (n=1,039). Asterisk indicates that ALPK1 was manually added to the list. **(B)** Resources for generating the list of human cancer-associated genes. **(C)** Human genes excluded from the feline bait set. Shown are the gene symbol from OncoKB, and the reason for exclusion from the feline targeted gene bait design. Genes were excluded if the given gene represented a large loci, pseudogene or non-coding RNA (Excluded_locus), if there was no ortholog identified in the cat genome (No_ortholog), if the cat ortholog was annotated on an unplace or unlocalised scaffold (Unplaced/unlocalised_cat_scaffold) or if there was no bait coverage across the entire gene (No_bait_coverage; n=61). **(D)** Final list of feline orthologs included in the bait set (n=978). Note: there are 978 Ensembl IDs but 986 lines in the file because in some cases there are one-to-many and many-to-many orthologous relationships between the human gene and cat genes.

Table S15. Somatic variants removed after manual inspection. A summary of the reasons for variant removal and list of the variants removed. Tumor type abbreviations: cSCC, cutaneous squamous cell carcinoma; LUCA, lung adenocarcinoma; LYM, lymphoma; MAM_WES, whole exome sequencing of mammary carcinoma; MEN, meningioma.

Table S16. Samples used for copy number calling. (**A**) Normal samples used as a reference panel for copy number calling. (**B**) Tumor samples excluded from copy number calling (see materials and methods for details); all other tumor samples were included in the analysis.

Table S17. Reagents for validation of hotspots by Sanger sequencing. (A) Primer sequences. **(B)** PCR reagents and conditions.

Table S18. Tumoroid culture medium.
